# Structure-Based Design of α-Substituted Mercaptoacetamides as Inhibitors of the Virulence Factor LasB from *Pseudomonas aeruginosa*

**DOI:** 10.1021/acsinfecdis.1c00628

**Published:** 2022-04-22

**Authors:** Cansu Kaya, Isabell Walter, Alaa Alhayek, Roya Shafiei, Gwenaëlle Jézéquel, Anastasia Andreas, Jelena Konstantinović, Esther Schönauer, Asfandyar Sikandar, Jörg Haupenthal, Rolf Müller, Hans Brandstetter, Rolf W. Hartmann, Anna K.H. Hirsch

**Affiliations:** † Helmholtz Institute for Pharmaceutical Research Saarland (HIPS)—Helmholtz Centre for Infection Research (HZI), Campus E8.1, 66123 Saarbrücken, Germany; ‡ Department of Pharmacy, Saarland University, Campus E8.1, 66123 Saarbrücken, Germany; § Department of Biosciences and Medical Biology, University of Salzburg, Hellbrunner Straße, 34, 5020 Salzburg, Austria; ∥ Helmholtz International Lab for Anti-Infectives, Campus E 8.1, 66123 Saarbrücken, Germany

**Keywords:** antibiotic resistance, structure-based design, virulence factors, LasB, heterocycles, mercaptoacetamides

## Abstract

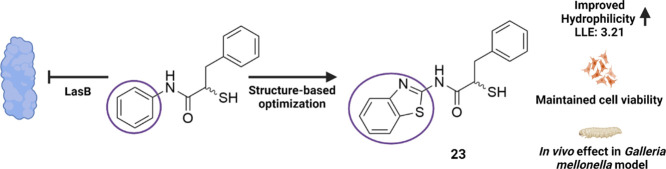

Antivirulence therapy has become a widely applicable method for fighting infections caused by multidrug-resistant bacteria. Among the many virulence factors produced by the Gram-negative bacterium *Pseudomonas aeruginosa*, elastase (LasB) stands out as an important target as it plays a pivotal role in the invasion of the host tissue and evasion of the immune response. In this work, we explored the recently reported LasB inhibitor class of α-benzyl-*N*-aryl mercaptoacetamides by exploiting the crystal structure of one of the compounds. Our exploration yielded inhibitors that maintained inhibitory activity, selectivity, and increased hydrophilicity. These inhibitors were found to reduce the pathogenicity of the bacteria and to maintain the integrity of lung and skin cells in the diseased state. Furthermore, two most promising compounds increased the survival rate of *Galleria mellonella* larvae treated with *P. aeruginosa* culture supernatant.

The lack of efficient therapeutics on the market for targeting resistant bacteria calls for the development of novel pathoblockers, agents capable of disarming bacteria by inhibiting their pathogenicity traits rather than killing them directly.^1,2^*P. aeruginosa* is a Gram-negative bacterium that causes around 10% of hospital-acquired infections and shows a high incidence in immunocompromised patients and in patients with cystic fibrosis.^3−6^ This opportunistic bacterium features several important mechanisms contributing to resistance development. Its efflux pumps can efficiently transport undesired antimicrobials out of the cell, while the secretion of β-lactamases eliminates the effect of β-lactam antibiotics by hydrolyzing their β-lactam ring.^7−10^ Furthermore, its low outer-membrane permeability prevents antibiotics from entering the cell and represents a challenge for the development of effective antibiotics.^11−13^ All these factors underline the urgent need to develop novel therapeutic options for the treatment of infections caused by these bacteria.

Rather than focusing on bacterial viability, combating resistant bacteria by targeting their virulence factors has recently gained more attention.^14,15^ These extracellular proteins are secreted by pathogenic bacteria and play important roles in various mechanisms, such as biofilm formation, invasion of host cells, and evasion of the immune response, thus contributing to the establishment and the progression of the disease.^16^ The development of inhibitors of such targets can facilitate the clearance of the pathogen either by the host immune system or by antibiotics.^17,18^ The main advantages of this method are the reduced selection pressure on the bacteria, which reduces the risk of resistance development by blocking the colonization and infiltration of the host, and the fact that the commensal bacteria remain unaffected.^14^ Although only a few small-molecule inhibitors have approached clinical application, numerous *in vitro* and *in vivo* studies support the efficacy of this strategy.^14,19^ One recent successful example is the antibody drug bezlotoxumab, which is market-approved and used as a toxin B neutralizer in the treatment of *Clostridium difficile* infections.^20^

LasB is considered as the key virulence factor secreted by *Pseudomonas aeruginosa*.^21^ It is a zinc-metalloprotease responsible for the pathogenic invasion of tissues and development of acute infections.^16,22^ It can degrade elastin, fibrin, and collagen, which are the vital components of lung tissue, blood vessels, and skin.^23^ It is also involved in the inactivation of human immunoglobulins A and G as well as the cytokines gamma-interferon and tumor necrosis factor alpha.^24−29^ All these collective mechanisms of LasB make it an attractive target for an antivirulence-based therapy.

Over the past few years, various inhibitor classes such as natural products,^29,30^ phosphoramidon (Pam),^31^ and several nonpeptidic compounds^32^ have been reported as inhibitors of LasB. Virtual-screening campaigns also reported fragment-like inhibitors with submicromolar activity (Figure 1, compound **1**).^33^ Small synthetic molecules such as thiols, hydroxamates, or mercaptoacetamides^34−39^ are commonly reported because of their metal-chelating motifs (Figure 1, compound **2**). By successfully applying fragment-merging we recently identified α-benzyl-*N*-aryl mercaptoacetamides as potent LasB inhibitors that are highly selective over a range of human metalloenzymes (Figure 1, compound **3**).^40^

**Figure 1:**
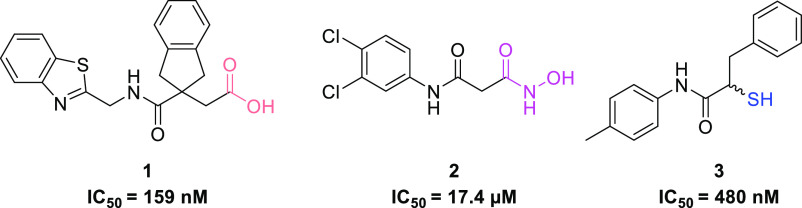
Structures of reported LasB inhibitors.^33,38,40^ Zinc-binding moieties are colored.

The major bottleneck in the development of potent LasB inhibitors is the problem of selectivity with respect to mammalian metalloenzymes, which play a prominent role in metabolism.^41^ Matrix metalloproteases (MMPs) are a family of zinc-dependent endopeptidases bearing a Zn^2+^ ion in their catalytical domain, posing a potential selectivity issue for inhibitors with zinc-chelating motifs.^42^ Based on the depth of their S1′ binding pocket, MMPs are divided into three classes: deep, intermediate, and shallow. Considering these differences in structure, pre-assessment of selectivity for designed inhibitors is important to obtain potent inhibitors with an acceptable selectivity profile against these off-targets.

We recently reported a successful fragment-merging strategy leading to the discovery of a highly selective and potent class of α-benzyl-*N*-aryl mercaptoacetamides as LasB inhibitors.^40^ Identification of compound **5** in Figure 2 in combination with its X-ray crystal structure with LasB allowed us to rationalize the binding mode of this class. A 12-fold boost in potency (IC_50_**=** 0.48 ± 0.04 μM) observed for inhibitor **3** compared to compound **4** also resulted in an improved *in vivo* effect in a *Galleria mellonella* model, demonstrating the success of this class in reducing bacterial pathogenicity.

**Figure 2:**
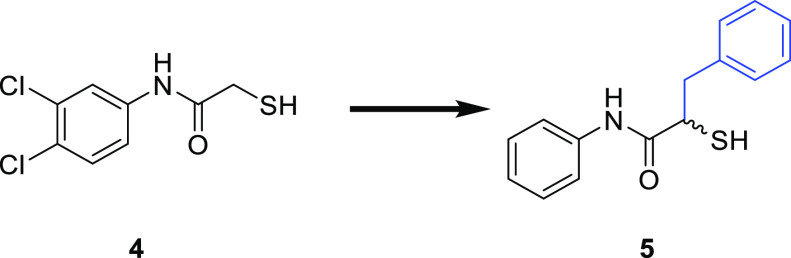
Structure of previously reported LasB inhibitors *N*-aryl mercaptoacetamide **4**(43) and α-benzyl-*N*-aryl mercaptoacetamide derivative **5**.^40^

In this work, we embarked on the multiparameter optimization of compound **5** aided by structure-based design. We synthesized seven derivatives by varying the substituents on both aryl rings and nine derivatives in which the *N*-aryl ring was replaced with various heterocycles and evaluated their inhibitory activity against LasB. To demonstrate the potential of the optimized inhibitors, we profiled them in terms of their activity, selectivity, and performance in whole-cell and *in vivo* models. We identified promising inhibitors that maintained efficacy and selectivity compared to compounds **3** and **5**. These inhibitors also reduced the pathogenicity of *P. aeruginosaLasB* during the diseased state in lung and skin cell lines. Demonstration of *in vivo* efficacy in a *G. mellonella* model highlights the potential of this class of inhibitors as effective antivirulence agents.

## Results and Discussion

### Synthesis and Evaluation of α-Benzyl-*N*-aryl Mercaptoacetamide Derivatives

**Scheme 1:**
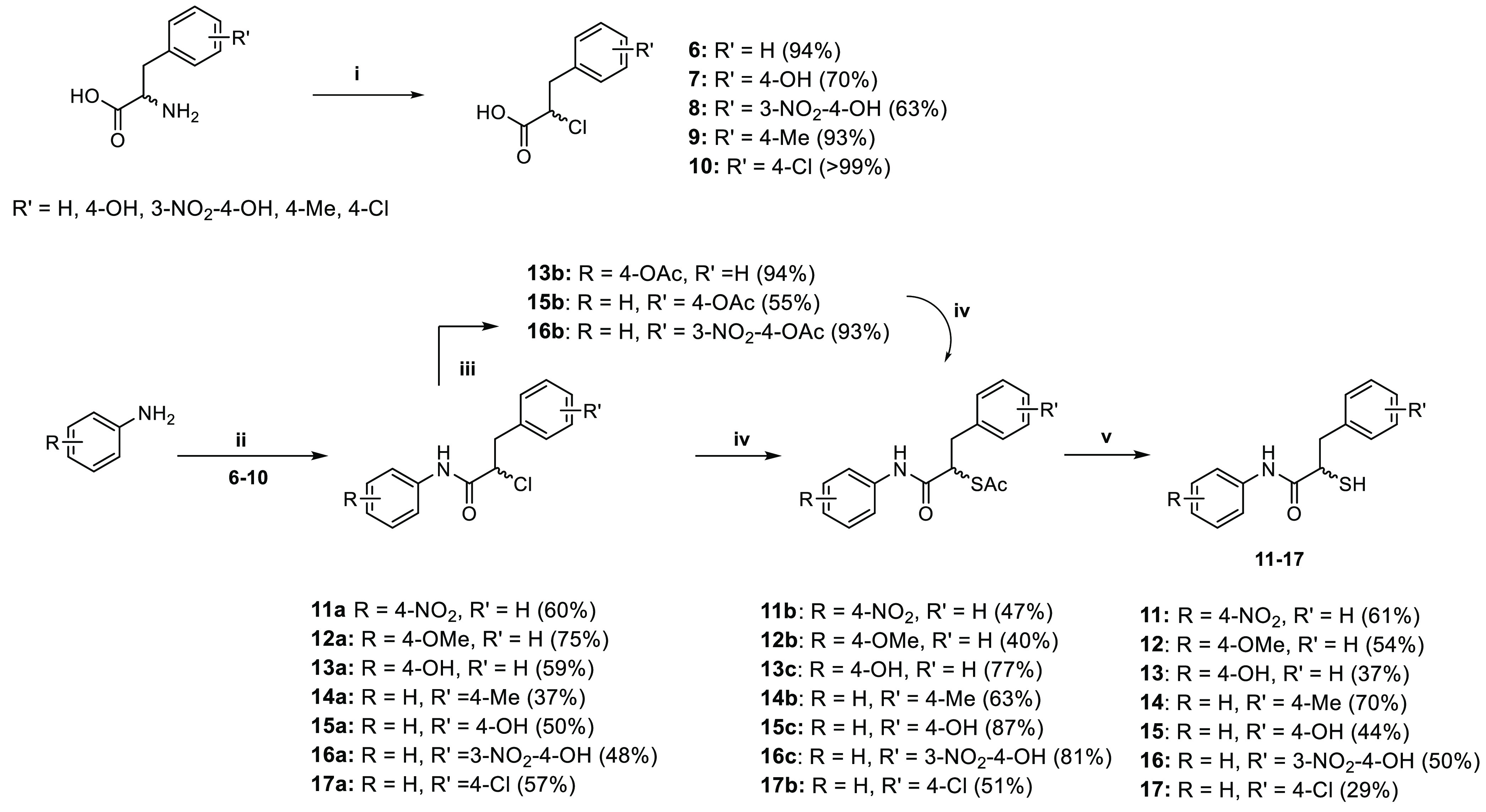
Synthetic Scheme of the α-Benzyl-*N*-aryl Mercaptoacetamide Class (i) sodium nitrite, 6 N HCl, 0 °C–r.t., 16 h; (ii) thionyl chloride, DMF, 70 °C, 1 h, aniline derivative, DMF, 0 °C–r.t., 16 h; (iii) Et_3_N, DMAP, DCM, acetic anhydride, 0 °C–r.t., 30 min; (iv) potassium thioacetate, acetone, r.t., 5 h; (v) 2 M aq. NaOH solution, MeOH, r.t., 1.5 h.

As we observed a 12-fold improvement in potency by the introduction of a small-sized methyl substituent on the *N*-aryl ring, we first analyzed the effect of other small-sized substituents on activity. Consequently, we synthesized seven derivatives bearing various substituents on both aromatic rings and evaluated their inhibitory activity against LasB. We previously identified the *para*-position to be the most favorable for a methyl group on the *N*-aryl ring. Following this observation, we introduced nitro, methoxy, and hydroxyl groups in the same position. The synthetic route is shown in Scheme 1.

The synthesis started with diazotization and subsequent chlorination of the corresponding commercially available racemic amino acids.^44^ Coupling of the α-chloro carboxylic acid (**6**–**10**) with the respective aniline gave the desired amide function (**11a**–**17a**). Derivatives containing hydroxyl groups were protected by a reaction with acetic anhydride (**13b**, **15b**, and **16b**). The thioacetate function was introduced via an SN2 reaction using potassium thioacetate. The final deprotection of the thioacetate function under basic conditions yielded compounds **11**–**17** in 29–70% yield as free thiol. The inhibitory activity of the final compounds against LasB was determined as previously reported (Table 1).^43^

**Table 1:**
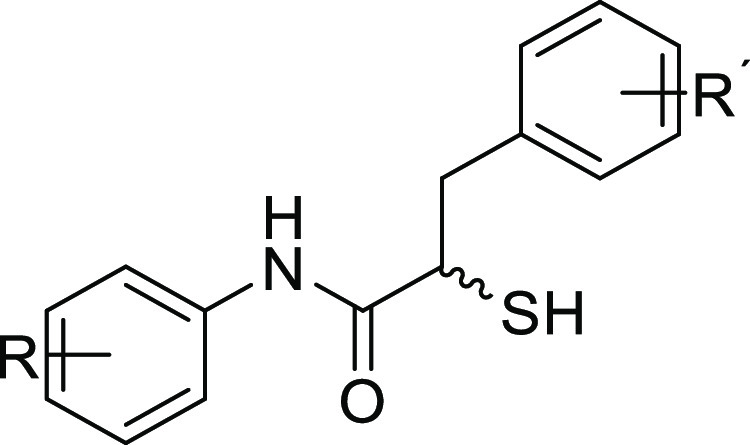
Structures and Inhibitory Activities of α-Benzyl *N*-aryl Mercaptoacetamide Derivatives **3**, **5**, and **11**–**17** against LasB^a^

compound	R	R′	IC_50_ (μM)
**3**	4-Me	H	0.48 ± 0.04
**5**	H	H	1.2 ± 0.1
**11**	4-NO_2_	H	1.0 ± 0.1
**12**	4-OMe	H	0.7 ± 0.03
**13**	4-OH	H	0.6 ± 0.04
**14**	H	4-Me	2.8 ± 0.3
**15**	H	4-OH	7.4 ± 0.6
**16**	H	3-NO_2_-4-OH	2.5 ± 0.2
**17**	H	4-Cl	1.1 ± 0.1

a Mean and SD values of at least two independent experiments.

The electron-withdrawing nitro substituent in the *para*-position in compound **11** proved to be less beneficial for the activity compared to the methyl group in compound **3**. A slight improvement in potency was achieved through the methoxy group in compound **12**. Based on this observation, we synthesized compound **13** with a hydroxyl group, which maintained the activity in a similar range as compounds **11** and **12**.

Overall, electron-donating substituents on the *N*-aryl ring proved to be more beneficial for the activity, irrespective of their hydrophilicity (**12** and **13**), while electron-withdrawing, polar substituents such as nitro group in compound **11** did not significantly improve the activity compared to compound **3**.

Introduction of various substituents on the benzyl ring in *para*-position yielded mainly unfavorable interactions, with the exception of compound **17**. Introducing a methyl group in the *para*-position in compound **14** only led to a twofold decrease in activity compared to compound **5**. The hydrophilic hydroxyl group led to a 15-fold decrease in activity of compound **15** as compared to compound **3**. Even though the addition of a strong electron-withdrawing nitro substituent in the *meta*-position in compound **16** compensated for the loss in activity, it remained low in comparison to the modifications on the *N*-aryl ring. Introduction of a *para*-chloro group in compound **17** increased the activity to a similar range compared to the modifications on the *N*-aryl ring, showing a slight increase compared to compound **5**.

These observations imply that a chloro substituent seems to be beneficial for the activity; however, a deeper exploration of various regioisomers and combinations with the *N*-aryl ring modifications is necessary for fine-tuning of the activity.

### Replacement of the *N*-Aryl Ring with Heterocycles

**Figure 3:**
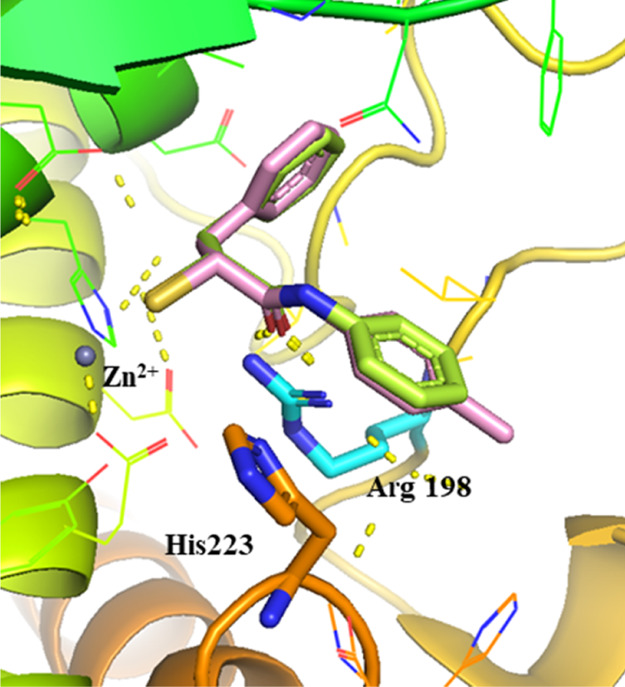
Superimposition of LasB (cyan surface) in complex with compound **5** (lime green, major conformation shown, PDB code: 7OC7) and the modeled pose of hit structure **3** (pink) with key interacting residues. The phenyl group occupies the S1′ binding site of the enzyme. The active site Zn^2+^ cation is shown as a gray sphere. The interactions in the binding pocket of LasB are predicted by SeeSAR V.11.1, and all figures are visualized using PyMOL V.2.5 software.^45^

The crystal structure of compound **5** allowed us to examine different strategies for further optimization.^40^ We previously discovered that the *N*-arylacetamide group in the S1′ pocket is stabilized by H-bonding and hydrophobic interactions. Introduction of a methyl substituent in *para*-position has improved the lipophilic interactions in the S1 pocket (Figure 3). To further improve these core interactions, we performed a molecular-docking study to replace the *N*-aryl ring with various heterocycles. By introducing this, we aimed to exploit the potential interactions such as H-bonding with the surrounding asparagine or arginine residues or π–π interactions with histidine residues.

Heterocycles are utilized in medicinal chemistry for the tuning of various physicochemical properties such as polarity, H-bonding capacity, and solubility. Pyridines, thiazoles, and benzimidazoles are commonly present in many natural products and in anti-infective drugs, providing diverse pharmaceutical applications.^46,47^

**Figure 4:**
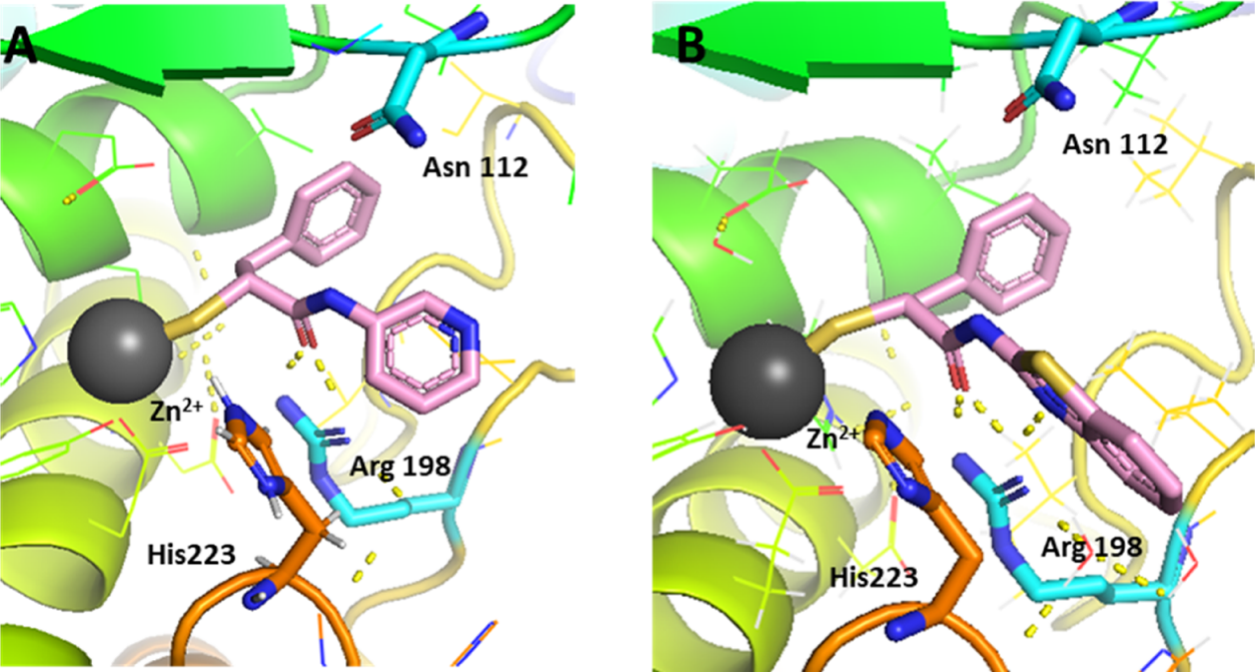
Selected docking poses for **(A)** 3-pyridine and **(B)** benzothiazole replacement. The interactions in the binding pocket of LasB (PDB code: 7OC7) are predicted by SeeSAR V.11.1 and visualized using PyMOL V.2.5 software. The dashed lines represent H bonds of less than 2.15 Å.

We selected several heterocycles differing in size and substituents and generated docking poses in the binding pocket of LasB using SeeSAR V.11.1, and visualized the interactions with PyMOL V.2.5 software (Figure S1).^45,48^ Figures showing docked compounds as stick representation were generated using PyMOL V.2.5 software. As we previously observed a preference for the *R*-configuration of the ligands in the binding pocket of LasB, all compounds were docked in their *R*-configuration. Predicted interactions in the binding pocket for two selected pyridine and benzothiazole derivatives are shown in Figure 4.

Upon replacement of the *N*-aryl ring with a pyridyl ring, the docking study predicted similar hydrophobic interactions of the benzyl ring with Val137 and Leu197 and a cation−π interaction with Arg198 as in compound **5** (Figure 4A). Additionally, a potential H-bond of the N atom in the ring with Asn112 could be predicted. Introducing a slightly larger benzothiazolyl ring (Figure 4B) led to some additional π–π stacking interactions with His223 residues along with cation−π interactions with Arg198. In most of the docking poses, the orientation of the heterocyclic compounds did not differ significantly from the crystal structure of **5** in complex with LasB.

### Synthesis and Evaluation of α-Benzyl *N*-Heteroaryl Mercaptoacetamide Derivatives

**Scheme 2:**
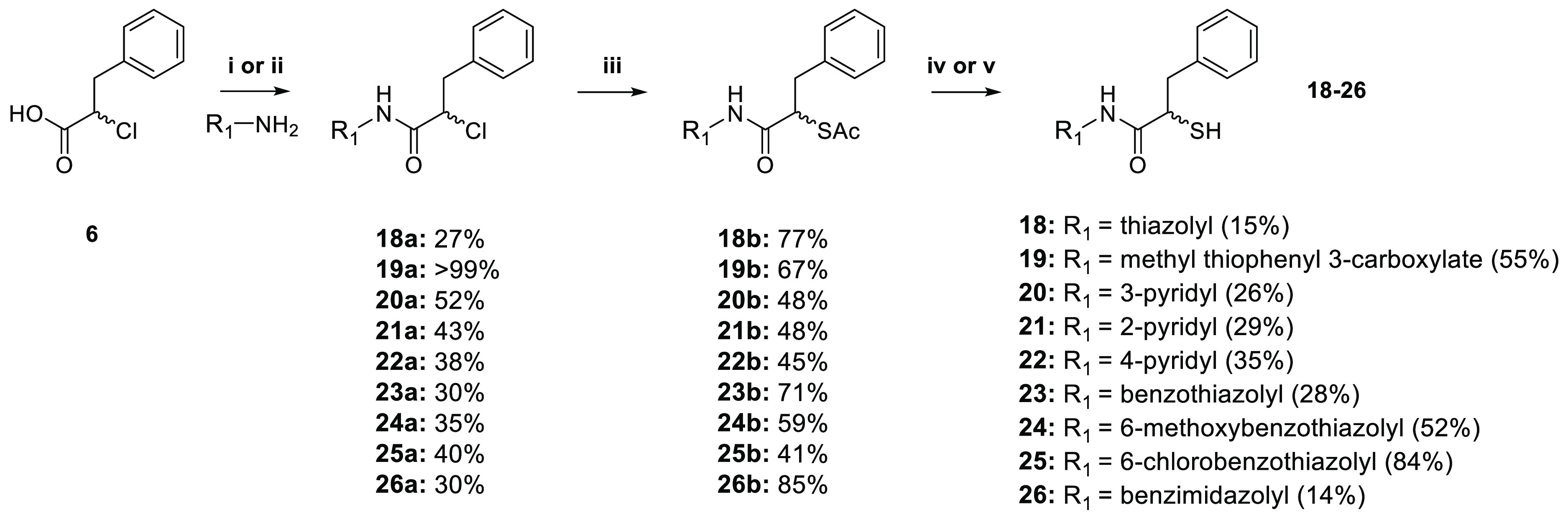
Synthetic Scheme of Heterocyclic Derivatives (i) Et_3_N, ethyl chloroformate, THF, r.t. overnight or (ii) HATU, DIEA, DCM, overnight; (iii) potassium thioacetate, acetone, r.t., 5 h; (iv) 2 M aq. NaOH solution, MeOH, r.t., 1.5 h or (v) AcCl, MeOH, r.t., 30–40 h.

Based on the input from docking, we selected and synthesized nine heterocyclic compounds. The synthetic route is summarized in Scheme 2.

The synthesis of heterocyclic derivatives **18**–**26** was achieved by coupling compound **6** with the corresponding heterocyclic anilines using either ethyl chloroformate or 1-[bis(dimethylamino)methylene]-1*H*-1,2,3-triazolo[4,5-*b*] pyridinium 3-oxide hexafluorophosphate (HATU) as the coupling reagent. Nucleophilic substitution of chlorine yielded the corresponding thioacetate intermediates **18b**–**26b**, which were hydrolyzed under basic conditions to afford free thiol derivatives **18**–**20** and **23**–**26** in moderate-to-good yield (14–84%). For the final compounds **21** and **22**, hydrolysis was performed under acidic conditions, with yields of 29 and 35%, respectively.

IC_50_ values for all nine derivatives against LasB were determined as reported previously (Table 2).^43^

**Table 2:**
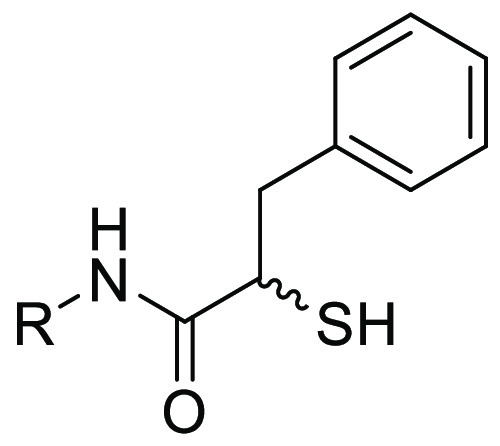

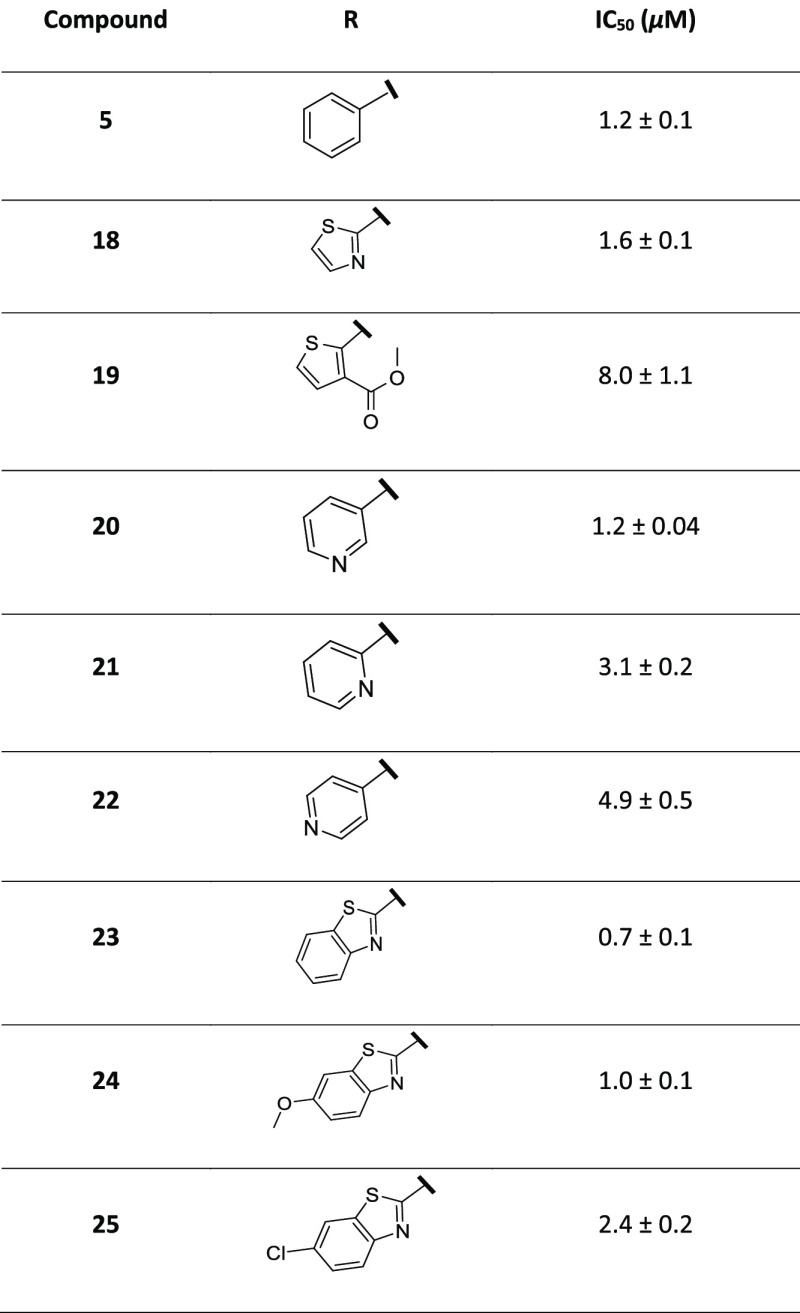
Structures and Inhibitory Activities of α-Benzyl-*N*-heteroaryl Mercaptoacetamides **18–26** against LasB^a^

a Means and SD of at least two independent experiments.

Replacement of the *N*-aryl ring with a thiazolyl group in compound **18** maintained the potency in the range of compound **5**. Interestingly, with a relatively small substituent, methyl thiophenyl 3-carboxylate in compound **19**, we observed an almost fivefold drop in IC_50_ value, presumably caused by unfavorable interactions due to the highly hydrophobic nature of the binding pocket.

We then explored pyridyl analogues in compounds **20**, **21**, and **22**. Compounds **21** and **22** demonstrated a two-fold decrease in potency compared to compound **5**, whereas compound **20** with a 3-pyridyl group did not improve the activity further. Nevertheless, among the three regioisomers, compound **20** demonstrated that the *meta*-position is most favorable for the potency.

**Figure 5:**
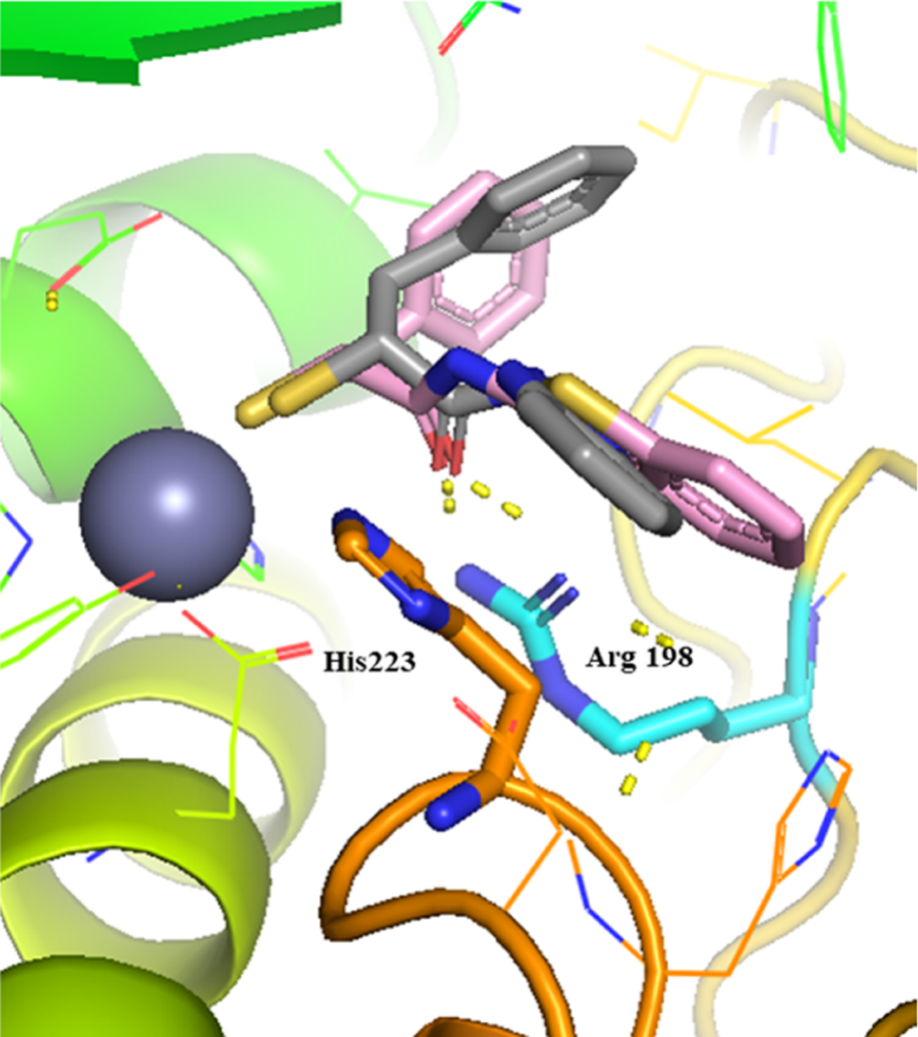
Superimposition of compound **23** (pink) and compound **26** (light gray) in the binding pocket of LasB (PDB code: 7OC7). The active site Zn^2+^ cation is shown as a gray sphere. The dashed lines represent H bonds of less than 2.15 Å.

Upon the introduction of a larger benzothiazolyl ring in compound **23**, the activity increased two-fold compared to compounds **18** and **20**. The introduction of this ring also proved to be important for the activity when compared to *N*-phenyl derivative **5**. This improvement presumably stems from the additional π–π stacking with the surrounding histidine residues for compound **23**, as predicted by the docking poses. Although similar in size, the benzimidazolyl compound **26** led to a dramatic decrease in the inhibitory activity. The comparison of interactions of the two structures in the binding pocket of LasB (Figure 5) reveals a slightly different binding mode for compound **26**, lacking some key interactions like H-bonding with the surrounding Arg198 residue compared to compound **23**. These observations highlight the importance of the correct heterocycle-mediated interactions within the binding pocket for improving potency.

As much as the ring size, the nature of the substituents also plays a role in the fine-tuning of the activity, as depicted by the threefold decrease in the activity of compound **25** with a chloro-substituted benzothiazolyl ring compared to compound **24** bearing a methoxy group on the benzothiazolyl ring.

Although the replacement of the *N*-aryl ring by heterocycles did not significantly improve the activity compared to compound **5**, compound **23** with a benzothiazolyl ring demonstrated an activity in the range similar to that of our previous hit compound **3**, while adding a slightly more hydrophilic nature to this class of inhibitors. This observation could be valuable in the future formulation studies of these inhibitors to overcome the potential solubility issues by lowering their log*D* values. Indeed, the calculation of ligand efficiency (LE) and lipophilic ligand efficiency (LLE) of compounds **3** (LE: 0.43, LLE: 2.37) and **23** (LE: 0.48, LLE: 3.21) revealed that we were able to improve the hydrophilicity by maintaining LE and the inhibitory activity in the same range.

To further demonstrate the potential of these inhibitors as pathoblockers against LasB, we selected compounds **12** and **13** along with the two heterocyclic derivatives, compounds **23** and **24**, and evaluated them further in several *in vitro* and *in vivo* assays.

### Targeting Other Virulence Factors

We previously demonstrated that the inhibitors of LasB can also act against bacterial collagenases.^49^ Collagenase H (ColH), secreted by the Gram-positive bacterium *C. histolyticum*, is a zinc-containing enzyme that causes tissue destruction by degrading collagen and is involved in various diseases.^50^ Similar to LasB, this extracellular metalloenzyme is capable of invading the host cell and acquiring nutrients to evade the immune defense. Consequently, we evaluated the inhibitory activity of our LasB inhibitors against this virulence factor.

The IC_50_ values were in the low nanomolar range (Table S1) for several selected α-benzyl-*N*-aryl derivatives, indicating the potential of this class for broad-spectrum inhibition of bacterial metalloproteases.

Among the four selected heterocyclic derivatives, only compound **23** showed a significant inhibition of ColH (*K*_*i*_: 0.1 ± 0.01 μM). This observation is noteworthy, as this compound is also a potent inhibitor of LasB.

### Antibacterial Activity

To rule out possible antibacterial activities, we assessed the inhibitory effect of compounds **13** and **23** against *P. aeruginosa* PA14. The minimum inhibitory concentration (MIC) assay showed no reduction of bacterial density up to 100 μM for both inhibitors, as expected for antivirulence agents.

### Selectivity against MMPs and TACE as Human Off-Targets

Inhibition of human zinc-containing enzymes is described frequently for inhibitors of LasB, hindering the development of selective compounds. MMPs are calcium-dependent zinc-metalloenzymes, playing crucial roles in the human body.^51^

To confirm the excellent selectivity we had previously reported for this class of inhibitors, we tested compounds **12**, **13**, and **23** for their activity against six representative MMPs and the three human off-targets, tumor necrosis factor-α-converting enzyme (TACE) or ADAM17, HDAC-3, and HDAC-8 (Table 3).^52,53^ Compound **3** is shown in Table 3 for comparison.

**Table 3:** Activities of Four LasB Inhibitors against the Selected MMPs (% of inhibition at 100 μM) and Further Human Off-Targets^a^

	**3**	**12**	**13**	**23**
MMP-1	n.i.	n.i.	n.i.	n.i.
MMP-2	n.i.	n.i.	n.i.	n.i.
MMP-3	n.i.	n.i.	n.i.	n.i.
MMP-7	n.i.	n.i.	n.i.	n.i.
MMP-8	n.i.	12 ± 2	19 ± 4	n.i.
MMP-14	n.i.	n.i.	n.i.	n.i.
IC_50_ (μM)				
ADAM17	4.8 ± 1.5	4.1 ± 0.1	2.3 ± 1.4	10.4 ± 0.2
HDAC-3	>100	>100	>250	>100
HDAC-8	>100	>100	>250	>100

a n.i. = <10% inhibition at 100 μM. Means and SD of at least two independent experiments.

All tested inhibitors demonstrated a high selectivity over MMPs, whereas they showed a relatively low selectivity for TACE (ADAM17), with IC_50_ values between 2 and 10 μM. Therefore, optimization strategies to improve selectivity toward this target are still necessary to develop pathoblockers that are closer to a potential therapeutic application.

### Cytotoxicity

We next evaluated the cytotoxicity of compounds **13** and **23** against three human cell lines to further support the potential therapeutic use of our compounds. Both inhibitors did not show any toxicity against human hepatoma (HepG2), human embryonic kidney (HEK) 293, and adenocarcinomic human alveolar basal epithelial (A549) cells up to 100 μM.

### *In Vivo* Zebrafish Embryo Toxicity

In view of their potency, relatively high selectivity, and the lack of cytotoxicity, we next evaluated compounds **12** and **23** in an *in vivo* toxicity study using zebrafish embryos. These embryos possess a high degree of genetic similarity compared to the human genome, offering a feasible, medium-throughput *in vivo* toxicity screening.^54,55^ Additionally, the lethality and malformation during the development of embryonic zebrafish can also be assessed with this experiment. A maximum tolerated concentration (MTC) of ≤30 μM was obtained for compound **23** and ≤2 μM for compound **12** (Table S2).

### Validation of the Effect of LasB Inhibitors

Before validating the effect of selected inhibitors, we examined the cytotoxic effect of LasB-containing culture supernatant *in vitro*. To this end, wild-type (wt) PA14 supernatant and LasB knockout (Δ*lasB*) PA14 supernatant were investigated on A549 cells and human dermal fibroblasts (NHDF). As shown in Figure S2, the wt PA14 supernatant reduced the viability (determined with MTT assay) and showed a dose-dependent effect on both cell lines. A concentration of 15% (*v/v*) decreased the viability to 10 ± 5 and 40 ± 5% of A549 and NHDF after 1 day of incubation, respectively. In contrast, the Δ*lasB* PA14 supernatant exhibited a smaller effect on the viability after 1 day of incubation, as 15% (*v/v*) resulted in a viability of 80 ± 20% in both cell types. This effect was less prominent on both cell lines after 2 days of incubation. The effect on cell morphology and attachment of both supernatants at 15% (*v/v*) was also examined with bright-field imaging. The wt PA14 supernatant induced cell detachment and rounding, indicating cell death (Figures S3and S4). On the other hand, 15% (*v/v*) Δ*lasB* PA14 supernatant showed a negligible effect on cell morphology and attachment of both A549 and NHDF cells. These data underline the role of LasB in inducing cell death.

Due to their high inhibitory activity, low cytotoxicity, and high selectivity over human off-targets, we selected compounds **12**, **13**, **23,** and **24** to verify their effect against LasB in this cell-based assay. A mixture of various concentrations of compounds and 15% (*v/v*) wt PA14 supernatant or Δ*lasB* PA14 supernatant was prepared and incubated with the cells for 1 day.

**Figure 6:**
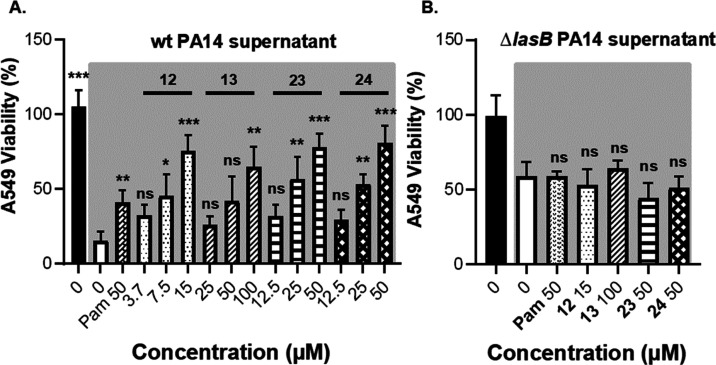
Viability of A549 cells treated with **12**, **13**, **23**, and **24** and 15% (*v/v*) wt PA14 supernatant or Δ*lasB* PA14 supernatant. **(A)** Dose-dependent effect of the compounds on the viability of A549 cells treated with wt PA14 supernatant; **(B)** no effect of the compounds on A549 cells treated with Δ*lasB* PA14 supernatant. The supernatant-treated groups are shown in gray background. Each graph is a representation of three independent experiments; mean ± SD. One-way ANOVA was performed for each experiment following Dunnett’s multiple comparison test. The mean of each column was compared with the mean of the negative control (ns: not significant, *: *p* ≤ 0.05, **: *p* ≤ 0.01, ***:*p* ≤ 0.001). wt PA14: wild-type *Pseudomonas aeruginosa*, Δ*lasB* PA14: LasB knockout *P. aeruginosa*, Pam: phosphoramidon.

Cell viability was assessed by the MTT assay, and live/dead cells were visualized by fluorescence microscopy. The MTT results in Figures 6 and S5 revealed that the selected compounds improved the viability of the cells and reduced the cytotoxic effect of the wt PA14 supernatant in a dose-dependent manner. For instance, compounds **23** and **24** showed an increase of 80 ± 15% in viability of A549 cells at 50 μM. This effect was smaller at lower concentrations (Figure 6a). Interestingly, they did not affect the activity of the Δ*lasB* PA14 supernatant, and the viability was similar to that of the control (no inhibitor) (Figure 6b).

Following this, live/dead staining showed an improved cell adhesion and live cell counts in both cell lines when treated with LasB inhibitors and wt PA14 supernatant (Figures S7 and S9), while showing no effect on the viability of the cells when challenged with Δ*lasB* PA14 supernatant and treated with our inhibitors (Figures S8 and S10). These data confirm that our compounds are selective and only active against LasB but not against other virulence targets in the supernatant. Moreover, these findings imply that our inhibitors can maintain the integrity of lung and skin cells during the disease state induced by *P. aeruginosa* and may reduce the bacterial propagation through the cells.

### *G. mellonellaIn Vivo* Model

To analyze the antivirulence activity of LasB inhibitors *in vivo*, we used a simple model based on *G. mellonella* larvae.

**Figure 7:**
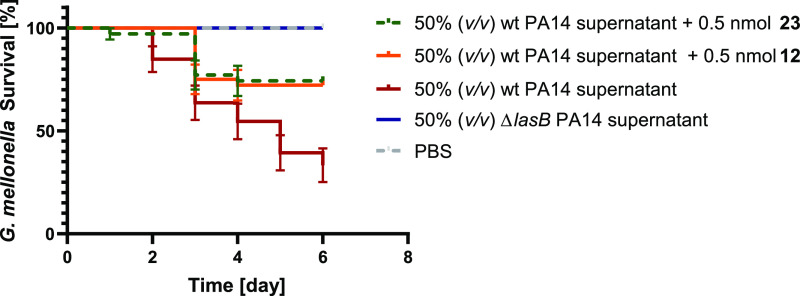
Kaplan–Meier survival analysis of larvae treated with 0.5 nmol of compounds **12** and **23** and 50% (*v/v*) wt PA14 supernatant. The survival was improved when wt PA14 supernatant-challenged larvae were treated with compounds. Each curve represents the results of three independent experiments. The statistical difference between the groups treated with wt PA14 supernatant and compounds **12** and **23** is *P* = 0.0013 and 0.0016 (log-rank test), respectively. The survival of the group treated with Δ*lasB* PA14 supernatant did not change compared to the wt PA14 supernatant-treated group (*P* = 0.0001). The survival of larvae treated with 0.5 nmol of the compounds (in sterile PBS) showed 100% viability. wt PA14: wild-type *Pseudomonas aeruginosa* and Δ*lasB* PA14: LasB knockout *P. aeruginosa*.

We have used this model previously to evaluate the treatment options for *P. aeruginosa*.^40^ We injected the larvae with a mixture of the compounds and wt PA14 supernatant, incubated them for 6 days, and recorded survival once per day (Figure 7). Our results show that wt PA14 supernatant reduced the survival of larvae to 35 ± 15% after 6 days of incubation, whereas the Δ*lasB* PA14 supernatant resulted in the survival of all larvae. Compared to the larvae treated with wt PA14 supernatant only, 0.5 mmol of compounds **12** and **23** increased the survival from 35 ± 15 to 70 ± 5% after 6 days compared to the non-treated larvae. Interestingly, the performance of both compounds **12** and **23** was comparable with that of compound **3** with an increase in survival rates up to ∼50%. These results validate that our inhibitors are potential candidates to block the pathogenicity of*P. aeruginosa*.

### Conclusions

In this work, we applied a structure-based optimization approach to extend the chemical space of the recently identified LasB inhibitor class of α-benzyl-*N*-aryl mercaptoacetamides. By exploiting the crystal structure of LasB with the previously reported inhibitor **5**, we first explored the effect of different substituents on both sides of the mercaptoacetamide core and synthesized seven derivatives. We then replaced the *N*-aryl ring with nine different heterocycles varying in size and substituents.

Although no notable improvement in potency was observed for these derivatives, we were able to identify three compounds (**12**, **13**, and **23**) with a maintained selectivity against selected human off-targets and a preserved low micromolar inhibitory activity against LasB compared to our previous hit compound **3**. With no signs of toxicity against human cell lines, these compounds also demonstrated a reduction in the pathogenicity of *P. aeruginosa* and maintained the integrity of lung and skin cells treated with the LasB-containing supernatant.

Inspired by these results, the *in vivo* efficacy of compounds **12** and **23** was further explored using an *in vivo* model based on*G. mellonella* larvae. The survival rate of the larvae challenged with wt PA14 supernatant was slightly increased in the presence of both compounds. This achievement is noteworthy, considering the gain in potency and increased hydrophilicity with this new class of compounds.

In addition to this, the inhibitory effect of this class of inhibitors against the structurally similar target ColH from *C. histolyticum* was also investigated, revealing several inhibitors with submicromolar *K*_i_ against this promising target, such as compound **23**.

In view of the current antimicrobial resistance crisis, our results highlight the potential of this class of inhibitors as attractive candidates for becoming effective pathoblockers in reducing bacterial pathogenicity while diminishing potential resistance development. Further optimization strategies on both binding pockets should be explored to ensure an improved physicochemical and pharmacokinetic profile and to address the potential stability issues associated with the free thiol group in this class of inhibitors.

## Methods

### General Chemistry

All reagents obtained from commercial suppliers were used without further purification. Procedures were not optimized regarding yield. NMR spectra were recorded on a Bruker AV 500 (500 MHz) spectrometer at room temperature. Chemical shifts are given in parts per million (ppm) and referenced against the residual proton, ^1^H, or carbon, ^13^C, resonances of the >99% deuterated solvents as internal reference. Coupling constants (*J*) are given in hertz (Hz). Data are reported as follows: chemical shift, multiplicity (s = singlet, d = doublet, t = triplet, dd = doublet of doublets, dt = doublet of triplets, m = multiplet, br = broad), and combinations of these coupling constants and integration. Liquid chromatography–mass spectrometry (LC–MS) was performed on an LC–MS system, consisting of a DionexUltiMate 3000 pump, an autosampler, a column compartment, and a detector (Thermo Fisher Scientific, Dreieich, Germany), and an ESI quadrupole MS system (MSQ Plus or ISQ EC, Thermo Fisher Scientific, Dreieich, Germany). Flash chromatography was performed using an automated flash chromatography system CombiFlash Rf+ (Teledyne Isco, Lincoln, NE, USA) equipped with RediSepRf silica columns (Axel Semrau, Sprockhövel Germany) or Chromabond Flash C18 columns (Macherey-Nagel, Düren, Germany). High-resolution mass was determined by LC–MS/MS using a Thermo Scientific Q Exactive Focus Orbitrap LC–MS/MS system. The purity of the final compounds was determined by LC–MS, using the area percentage method on the UV trace recorded at a wavelength of 254 nm, and found to be >95%.

### Synthesis of Intermediates and Final Compounds

#### General Procedure **A**: Synthesis of Chloro Acid Derivatives **6**–**10** from Amino Acid

Amino acid (1.0 equiv) was dissolved in 6 N HCl (2 mL/mmol or until mostly dissolved) under nitrogen atmosphere and cooled to −5 °C. NaNO_2_ (1.5–2.5 equiv) was dissolved in water (0.3 mL/mmol amino acid) and added dropwise slowly. The mixture was stirred overnight while warming to r.t. The reaction mixture was extracted with EtOAc/THF (3:1). The combined organic extracts were washed with saturated aq. NaCl solution and dried over anhydrous Na_2_SO_4_ and filtered. The solvent was removed under reduced pressure to obtain the product. The crude was used in the next steps without further purification.

#### General Procedure **B**: Synthesis of Derivatives **11a**–**17a** Using Thionyl Chloride

The acid (1.0 equiv), SOCl_2_ (2.0 equiv), and a few drops of DMF were heated to 70 °C for 1 h. The cooled mixture was added dropwise to a solution of the corresponding aniline (1.1 equiv) in DMF (1 mL/mmol) at 0 °C. The mixture was stirred overnight at r.t. The reaction mixture was quenched with water and extracted with EtOAc (3×). The combined organic extracts were washed with saturated aqueous NaCl solution and dried over anhydrous Na_2_SO_4_ and filtered. The solvent was removed under reduced pressure to obtain the crude product. The purification was done by column chromatography or flash chromatography.

#### General Procedure **B-1**: Synthesis of Coupling Derivatives **18a** and **23a**–**25a** Using Ethylchloroformate as the Coupling Reagent

The acid (1.2 equiv) was dissolved in THF and cooled in an ice bath. Et_3_N (1.2 equiv) was added, followed by the addition of ClCO_2_Et (1.3 equiv). After 5 min, the ice bath was removed, and the mixture was stirred at r.t. for 30 min. The corresponding amine (1.0 equiv) was slowly added. The reaction was monitored using TLC or LC–MS. After the reaction was completed, volatiles were evaporated under reduced pressure, and the crude product was purified using column chromatography.

#### General Procedure **B-2**: Synthesis of Coupling Derivatives **17a**, **19a**–**22a,** and **26a** Using HATU as the Coupling Reagent

The acid (1.5 equiv) was dissolved in DCM (10 mL) at r.t., and to this DIEA (1.5 equiv) and HATU (1.5 equiv) were added. The corresponding aniline (1 equiv) was then added to this mixture, and the reaction was monitored by LC–MS. The mixture was extracted with saturated aqueous NaCl solution (1×) and then dried over anhydrous Na_2_SO_4_ and filtered. The crude was purified using reverse phase flash chromatography (H_2_O + 0.1 % FA/ACN + 0.1% FA 95:5 → 5:95).

#### General Procedure **C**: Protection of the Hydroxyl Group in Derivatives **13b**, **15b,** and **16b**

The amide (1.0 equiv), Et_3_N (2.0 equiv), and 4-dimethylaminopyridine (0.03 equiv) were dissolved in DCM (5 mL/mmol) and cooled to 0 °C. Acetic anhydride (2.0 equiv) was added dropwise. The solution was warmed to r.t. and stirred for 30 min. The reaction mixture was washed with DCM, washed with saturated aqueous NaCl solution, dried over anhydrous Na_2_SO_4_, and filtered. The solvent was removed under reduced pressure to obtain the crude product.

#### General Procedure **D**: Synthesis of Thioacetate Derivatives **11b**, **12b**, **13c**, **14b**, **15c**, **16c,** and **17b**–**26b**

The corresponding chloro derivative (1.0 equiv) was dissolved in acetone under argon atmosphere. To this solution, CH_3_COSK (1.5–3.0 equiv) was added, and the mixture was stirred for 2–6 h at r.t. It was monitored by TLC or LC–MS. The reaction mixture was quenched with water and extracted with EtOAc (3×). The combined organic extracts were washed with saturated aqueous NaCl solution, dried over anhydrous Na_2_SO_4_, and filtered. The solvent was removed under reduced pressure to obtain the crude product. The purification was done by flash chromatography.

#### General Procedure **E**: Hydrolysis of Thioacetate for Derivatives **11**–**26**

Thioacetate (1.0 equiv) was dissolved in methanol (5 mL/mmol) under argon atmosphere, and 2 M aqueous NaOH solution (2.0 equiv) or solid NaOH (3.0 equiv) was added. The solution was stirred 1–3 h at r.t. before being quenched with 1 M HCl. The solution was extracted with EtOAc and washed with 0.5 M HCl. The combined organic extracts were washed with saturated aqueous NaCl solution and dried over anhydrous Na_2_SO_4_ and filtered. The solvent was removed under reduced pressure to obtain the crude product. The purification was done by column chromatography or preparative HPLC (H_2_O + 0.05% FA/ACN + 0.05% FA, 95:5 → 5:95). For more polar compounds, instead of quenching the reaction with 1 M HCl, the pH was adjusted to acidic using Amberlite IR-120. After filtration, Amberlite was washed with MeOH (3×), the solvent was evaporated, and the product was purified using preparative HPLC (H_2_O + 0.05% FA/ACN + 0.05% FA, 95:5 → 5:95). For compounds **21** and **22**, thioacetate (1.0 equiv) was dissolved in methanol (5 mL/mmol) under argon atmosphere, and acetyl chloride (15 equiv) was added dropwise over 10 h. The mixture was stirred at room temperature for 30–40 h and carefully monitored by LC–MS. Once the conversion was complete, the solvent was removed under reduced pressure to obtain the crude product. Purification was done by preparative HPLC (H_2_O + 0.05% FA/ACN + 0.05% FA, 95:5 → 5:95).

#### 2-Chloro-3-phenylpropanoic Acid (**6**)

Compound **6** was prepared according to general procedure **A**, using dl-phenylalanine (1 g, 6.0 mmol) and NaNO_2_ (1.46 g, 21.2 mmol). The crude product was obtained as a yellow oil and used without further purification (1.05 g, 94%). ^1^H NMR (500 MHz, CDCl_3_): δ ppm 7.37–7.24 (m, 5H), 4.51 (dd, *J* = 7.8, 6.9 Hz, 1H), 3.42 (dd, *J* = 14.0, 6.7 Hz, 1H), 3.21 (dd, *J* = 14.1, 7.9 Hz, 1H). MS (ESI^–^) *m/z*: 183.25 [M – H]^−^, 147.23 [M – H – HCl]^−^.

#### 2-Chloro-*N*-(4-methoxyphenyl)-3-phenylpropanamide (**12a**)

Compound **12a** was prepared according to the general procedure **B**, using compound **6** (200 mg, 1.08 mmol), SOCl_2_ (157 μL, 2.17 mmol), and *p*-anisidine (147 mg, 1.19 mmol). Purification was done by flash chromatography (Hex/EtOAc, 100:0 to 0:100). The product was obtained as a green solid (234 mg, 75%). ^1^H NMR (500 MHz, CDCl_3_): δ ppm 8.01 (br s, 1H), 7.38–7.24 (m, 7H), 6.90–6.86 (m, 2H), 4.71 (dd, *J* = 7.8, 4.4 Hz, 1H), 3.81 (s, 3H), 3.52 (dd, *J* = 14.3, 4.4 Hz, 1H), 3.32 (dd, *J* = 14.3, 7.6 Hz, 1H). MS (ESI^+^) *m/z*: 290.04 [M + H]^+^.

#### *S*-(1-((4-Methoxyphenyl)amino)-1-oxo-3-phenylpropan-2-yl) Ethanethioate (**12b**)

Compound **12b** was prepared according to the general procedure **D**, using compound **12a** (230 mg, 0.95 mmol) and potassium thioacetate (162 mg, 1.42 mmol). Purification was done by flash chromatography (Hex/EtOAc, 100:0 to 0:100). The product was obtained as a yellow solid (126 mg, 40%). ^1^H NMR (500 MHz, CDCl_3_): δ ppm 7.81 (br s, 1H), 7.37–7.34 (m, 2H), 7.33–7.23 (m, 5H), 6.86–6.81 (m, 2H), 4.28 (dd, *J* = 8.4, 7.2 Hz, 1H), 3.79 (s, 3H), 3.44 (dd, *J* = 14.0, 8.4 Hz, 1H), 3.01 (dd, *J* = 14.1, 7.1 Hz, 1H), 2.38 (s, 3H). ^13^C NMR (126 MHz, CDCl_3_): δ ppm 197.4, 168.3, 156.7, 137.9, 130.9, 129.5, 128.8, 127.2, 114.3, 121.8, 55.7, 48.7, 36.1, 30.7. MS (ESI^+^) *m/z*: 330.08 [M + H]^+^, 288.08 [M – Ac + 2H]^+^.

#### 2-Mercapto-*N*-(4-methoxyphenyl)-3-phenylpropanamide (**12**)

Compound **12** was prepared according to the general procedure **E**, using compound **12b** (95 mg, 0.29 mmol) and 2 M NaOH aqueous solution (290 μL, 0.58 mmol) in MeOH (2 mL). Purification was done by flash chromatography (Hex/EtOAc, 100:0 to 0:100). The final product was obtained as a white solid (45 mg, 54%). ^1^H NMR (500 MHz, CDCl_3_): δ ppm 7.90 (br s, 1H), 7.37–7.33 (m, 2H), 7.31 (d, *J* = 7.5 Hz, 2H), 7.28–7.23 (m, 3H), 6.89–6.84 (m, 2H), 3.80 (s, 3H), 3.70 (dt, *J* = 8.9, 6.6 Hz, 1H), 3.36 (dd, *J* = 13.7, 6.7 Hz, 1H), 3.24 (dd, *J* = 14.0, 6.4 Hz, 1H), 2.10 (d, *J* = 8.9 Hz, 1H). ^13^C NMR (126 MHz, CDCl_3_): δ ppm 169.5, 156.9, 137.5, 130.4, 129.6, 128.7, 127.3, 122.1, 114.3, 55.6, 45.9, 41.7. HRMS (ESI^+^) *m*/*z*: calcd for C_16_H_18_NO_2_S [M + H]^+^, 288.10527; found, 288.10453.

#### *N*-(Benzo[*d*]thiazol-2-yl)-2-chloro-3-phenylpropanamide (**23a**)

Compound **23a** was synthesized according to the general procedure **B-1**, using compound **6** (626 mg, 3.39 mmol), 2-aminobenzothiazole (422 mg, 2.81 mmol), Et_3_N (476 μL, 3.39 mmol), and ClCO_2_Et (355 μL, 3.72 mmol) in THF (33 mL). The final product was purified using flash chromatography (DCM/MeOH, 100:0 to 95:5). The final product was obtained as an off-white oil (324 mg, 30%). ^1^H NMR (500 MHz, CDCl_3_): δ ppm 7.86 (d, *J* = 7.9 Hz, 1H), 7.78 (m, 1H), 7.47 (t, *J* = 7.7 Hz, 1H), 7.36 (t, *J* = 7.6 Hz, 1H), 7.32–7.23 (m, 3H), 7.21–7.18 (m, 2H), 4.76–4.70 (m, 1H), 3.56–3.51 (m, 1H), 3.29 (dd, *J* = 14.4, 7.8 Hz, 1H). ^13^C NMR (126 MHz, CDCl_3_): δ ppm 167.1, 157.3, 148.2, 135.3, 132.3, 129.6, 128.8, 127.7, 127.72, 126.7, 124.6, 121.7, 121.3, 60.3, 41.2. MS (ESI^+^) *m/z*: 316.98 [M + H]^+^.

#### *S*-(1-(Benzo[*d*]thiazol-2-ylamino)-1-oxo-3-phenylpropan-2-yl) Ethanethioate (**23b**)

Compound **23b** was prepared according to the general procedure **D**, using compound **23a** (323 mg, 1.02 mmol) and potassium thioacetate (174 mg, 1.53 mmol) in acetone (10 mL). Purification was done by flash chromatography (Hex/DCM, 100:0 to 0:100). The final product was obtained as a yellow solid (257 mg, 71%). ^1^H NMR (500 MHz, acetone-*d*_6_): δ ppm 11.24 (s, 1H), 8.02–7.88 (m, 1H), 7.69 (d, *J* = 8.1 Hz, 1H), 7.43 (m, 1H), 7.35–7.26 (m, 5H), 7.24–7.17 (m, 1H), 4.72 (dd, *J* = 8.7, 6.9 Hz, 1H), 3.41 (dd, *J* = 13.8, 8.7 Hz, 1H), 3.06 (dd, *J* = 13.8, 6.9 Hz, 1H), 2.36 (s, 3H). ^13^C NMR (126 MHz, CDCl_3_): δ ppm 196.2, 169.1, 157.6, 148.4, 136.9, 132.3, 129.3, 128.8, 127.4, 126.5, 124.3, 121.5, 121.2, 47.9, 35.9, 30.5. MS (ESI^+^) *m/z*: 357.01 [M + H]^+^, 314.90 [M – Ac + H]^+^.

#### *N*-(Benzo[*d*]thiazol-2-yl)-2-mercapto-3-phenylpropanamide (**23**)

Compound **23** was prepared according to the general procedure **E**, using compound **23b** (128 mg, 0.36 mmol) and 2 M NaOH aqueous solution (359 μL, 0.72 mmol) in MeOH (3 mL). Purification was done by flash chromatography (Hex/EtOAc, 7:3). The final product was obtained as a white solid (30 mg, 28%).^1^H NMR (500 MHz, CDCl_3_): δ ppm 7.85 (d, *J* = 7.8 Hz 1H), 7.76 (d, *J* = 8.1, 1H), 7.49–7.44 (m, 1H), 7.39–7.36 (m, 1H), 7.29–7.27 (m, 1H), 7.25–7.15 (m, 4H), 3.87–3.80 (m, 1H), 3.40 (dd, *J* = 14.0, 7.0 Hz, 1H), 3.24 (dd, *J* = 14.0, 6.8 Hz, 1H), 2.26–2.17 (m, 1H).^13^C NMR (126 MHz, CDCl_3_): δ ppm 170.7, 159.0, 145.5, 136.7, 131.0, 129.4, 128.9, 127.5, 127.2, 125.0, 121.9, 120.2, 44.7, 41.1. HRMS (ESI^+^) *m*/*z*: calcd for C_16_H_15_N_2_OS_2_ [M + H]^+^, 315.06203; found, 315.06178.

## Supporting Information Available

Experimental methods; synthesis of intermediates and final compounds; NMR spectra of final compounds; LC–MS spectra of final compounds (PDF)

## Abbreviations

A549: lung carcinoma cell line
ColH: *Clostridium histolyticum* (*Hathewaya histolytica*) collagenase
DMEM: Dulbecco’s modified Eagle’s medium
DMAP: 4-dimethylaminopyridine
DCM: dichloromethane
HDAC: histone deacetylase
HEK293: embryonal kidney cell line
HepG2: hepatocellular carcinoma cell line
IC_50_: the half-maximal inhibitory concentration
LasB: *Pseudomonas aeruginosa* elastase
MIC: minimum inhibitory concentration
MMPs: human matrix metalloproteases
MTC: maximum tolerated concentration
SAR: structure–activity relationship
TACE: tumor necrosis factor-α-converting enzyme
TCEP: Tris(2-carboxyethyl)phosphine hydrochloride
THF: tetrahydrofuran
