# Supplementary material for: Structure-Based Design of α-Substituted Mercaptoacetamides as Inhibitors of the Virulence Factor LasB from Pseudomonas aeruginosa
Source: ACS Infect Dis. 2022 Apr 22;8(5):1010–21. doi: 10.1021/acsinfecdis.1c00628 (PMC9112332; doi:10.1021/acsinfecdis.1c00628)
Supplement: Supplementary file 1 — id1c00628_si_001.pdf [file id1c00628_si_001.pdf]

Supporting Information

Structure-based Design of α-Substituted Mercaptoacetamides as Inhibitors of the Virulence Factor LasB from *Pseudomonas aeruginos*a

Cansu Kaya,[1],[2] Isabell Walter,[1],[2] Alaa Alhayek,[1],[2] Roya Shafiei,[1],[2] Gwenaëlle Jézéquel,[1] Anastasia Andreas,[1],[2] Jelena Konstantinović,[1] Esther Schönauer,[3] Asfandyar Sikandar,[1] Jörg Haupenthal,[1] Rolf Müller,[1],[2],[4] Hans Brandstetter,[3] Rolf W. Hartmann,[1],[2] Anna K.H. Hirsch[1],[2],[4]*

[1] Helmholtz Institute for Pharmaceutical Research Saarland (HIPS) – Helmholtz Centre for Infection Research (HZI), Campus E8.1, 66123 Saarbrücken, Germany

[2] Department of Pharmacy, Saarland University, Campus E8.1, 66123 Saarbrücken, Germany

[3] Department of Biosciences and Medical Biology, University of Salzburg, Hellbrunner Straße, 34, 5020 Salzburg, Austria

[4] Helmholtz International Lab for Anti-Infectives, Campus E8.1, 66123 Saarbrücken, Germany

Table Of Contents

Experimental Methods 2

Synthesis of Intermediates and Final Compounds 4

Tables and Figures 19

NMR Spectra of Final Compounds 32

References 56

Experimental Methods

LasB Inhibition Assay. The purification of LasB from *P. aeruginosa* P14 supernatant as well as the subsequent performance of the FRET-based *in vitro* inhibition assay was performed as described previously.[1] All samples were run in duplicate for each condition, and experiments were performed independently at least twice.

***In vitro* ColH Inhibition Assay.** The purification of ColH-PD and determination of the inhibitory activities of the selected compounds were performed as described previously.[2,3] In short, enzyme and inhibitor or buffer control were preincubated for 1 h at RT, before the reactions were initiated by the addition of the quenched fluorescent substrate Mca-Ala-Gly-Pro-Pro-Gly-Pro-Dpa-Gly-Arg-NH2 (Mca = (7-Methoxycoumarin-4-yl)acetyl; Dpa = *N*-3-(2,4-dinitrophenyl)-*L*-2,3-diaminopropionyl) (FS1-1). The increase in fluorescence was monitored for 2 min 24 s (Excitation: 328 nm, Emission: 392 nm) at 25°C. The final concentrations were 2 nM ColH-PD, 10 µM compound, 250 mM Hepes pH 7.5, 400 mM NaCl, 10 mM $CaCl_2$, 10 µM $ZnCl_2$, 2% DMSO, and 2 µM FS1-1. The percentage of enzyme inhibition was calculated in relation to a reference without a compound added, only plus buffer control. For the K_i determination, the concentrations of the compound were optimized according to Murphy.[4] The apparent inhibition constant (K_i^{app}) value was determined by non-linear fitting to the Morrison equation[5] following a two-stage regression analysis strategy for tight-binding inhibitors.[6] Regression analysis was performed using GraphPad Prism 9.0.0 (Graph Pad Software, San Diego, CA, USA). The experiments were performed under first order conditions ($[S_0] \ll K_M$), which resulted in an approximation of the K_i^{app} to the true inhibition constant (K_i), and, therefore, the results are reported as K_i values.

Antibacterial Activity assay. Minimum inhibitory concentration (MIC) assays were performed as described previously.[1] The MIC value was higher than 100 μM for compounds **13** and **23**. At 100 µM, the bacterial growth was reduced by less than 10% for both compounds. All samples were run in duplicate for each condition, and experiments were performed independently at least twice.

Inhibition Assays with human off-targets. Assays focusing on the inhibition of human MMPs and ADAM17 were performed as described previously.[3,7] All samples were run in duplicate for each condition, and experiments were performed independently at least twice.

Cytotoxicity Assay. The toxicity of selected compounds toward HepG2, A549 and HEK293 cells was determined as described previously.[1,8] Compounds **13** and **23** showed no relevant cytotoxic behaviour against the human hepatoma cell line (HepG2), human embryonic kidney (HEK) 293 cells and adenocarcinomic human alveolar basal epithelial cells (A549) with IC_{50} values higher than 100 μM. All samples were run in duplicate for each condition, and experiments were performed independently at least twice.

Docking Studies. Modelling of derivatives of compound **5** in the LasB ligand binding pocket (PDB:7OC7) were performed using SeeSAR V.11.1 (BioSolveIT GmbH, Sankt Augustin, Germany)[9] software and the interactions are visualized using PyMOL Molecular Graphics System, V. 2.5 Schrödinger, LLC.[10]

Zebrafish Experiments. Maximum Tolerated Concentration (MTC) assay was performed with minor modifications according to the procedure described in literature.[11] After successful mating of parent fish from the AB wild-type line, embryos were collected, sorted and kept until the next day at 28 °C in 0.3× Danieau's medium [17 mM NaCl, 2 mM KCl, 1.8 mM $Ca(NO_3)_2$, 1.5 mM HEPES (pH 7.1–7.3), 0.12 mM $MgSO_4$ and 1.2 µM methylene blue]. The assay was

performed in 96-well plates using zebrafish embryos at 1 day post fertilization (dpf). Compound solutions in 0.3× Danieau's medium were prepared freshly on the day of experiment with a final DMSO concentration of 1% (*v/v*). Single zebrafish embryos were placed in a 96-well microtiter plate - one embryo per well and ten embryos per condition - and directly incubated in the corresponding compound solutions. The embryos were monitored daily via microscopy until 120 hours post fertilization (hpf) (Table S2). All described experiments were performed with zebrafish embryos younger than 120 hpf and are therefore not classified as animal experiments according to EU Directive 2010/63/EU. Protocols for husbandry and care of adult animals are in accordance with the German Animal Welfare Act (§11 Abs. 1 TierSchG).

Preparation of *P. aeruginosa* culture supernatants and LasB activity evaluation.

The mutant *P. aeruginosa* Δ*lasB* PA14 was kindly provided by the Häussler group (Twincore, Hannover, Germany). *P. aeruginosa* Δ*lasB* PA14 (parental strain: "*P. aeruginosa* PA14 (DSM 19882)") is a knockout mutant with markerless in-frame deletion (in frame deletion with pEX18Ap (no antibiotic resistance introduction)), as described in Casilag *et al*.[12] Overnight cultures of a single colony of wild-type (wt) and the LasB knockout (Δ*lasB*) PA14 strains were grown in lysogeny broth medium at 37 °C with constant shaking at 200 rpm. The next day, the culture was centrifuged at 4 °C, 5000 rpm for 30 min. Then, the supernatant was passed through a membrane filter of 0.2 μm to sterilize it, it wasaliquoted and stored at –80 °C until use. LasB activity of both supernatants was evaluated using the FRET-based assay which was described previously (Figure S11).

Cell-based *in vitro* experiments. A549 cells were purchased from Sigma Aldrich and NHDF cells were provided from Leibniz Institute for New Materials (INM) (Saarbrücken, Germany). Both cell lines were cultured in cell culture plates with a 150 X 20 mm diameter. The cells were incubated with Dulbecco's modified Eagle's medium (DMEM) (Gibco) supplemented with 10% (*v/v*) fetal bovine serum (FBS, Gibco) and 1% (*v/v*) Penicillin-Streptomycin (Pen-Strep) at 37 °C under 5% CO_2 in a humidified incubator. 50,000 NHDF cells/well and 100,000 A549 cells/well were seeded in 96-well plates (Greiner) and incubated for 24 h at 37 °C and 5% CO_2 so that the cells reached a confluency of 90%. For imaging purposes, the cells were plated on 96 well glass bottom plates (Cellvis). Next, the cells were treated with (0–25%) wt PA14 supernatant or Δ *lasB* PA14 supernatant to compare between their cytotoxic effects. 15% of each supernatant was used in the next experiments with compounds. To prevent disulfide formation of our compounds, we added tris(2-carboxyethyl) phosphine (TCEP) as a reducing agent and optimized its concentration in the assay before the evaluation of the compounds. A mixture of PA14 supernatant (*i.e.,* wt or Δ *lasB*), various concentrations of LasB inhibitors, 40 µM $ZnCl_2$, 40 µM $CaCl_2$ and 300 µM TCEP was preincubated for 30 min and directly added to the cells. The optimized concentration of TCEP did not show any toxic effect on cells and did not affect LasB activity. Phosphroamidon was included in the experiments as LasB reference inhibitor. A mixture of DMEM with TCEP, 40 µM $ZnCl_2$, 40 µM $CaCl_2$ and 1% DMSO was used as a control. To determine the cell viability, we conducted two different assays: an MTT assay and a live/dead staining followed by imaging using a Leica epifluorescence microscope (DMi8, Leica microsystem CMS GmbH). The MTT assay is a method that can be used to determine the metabolic activity of cells, since active cells are able to reduce the MTT dye (3-(4,5-Dimethylthiazol-2-yl)-2,5-Diphenyltetrazolium Bromide) to the purple formazan precipitate which can be dissolved, and its absorbance can be measured. In the MTT assay, the cells were washed twice with 200 µL of sterile PBS, followed by addition of DMEM containing 5 mg/mL of MTT solution. The cells were incubated at 37 °C, 5% CO_2 for 2 h. In a next step, the MTT solution was carefully removed and 200 µL of 100% DMSO

was added to dissolve the purple formazan crystals. After this, we measured the absorbance at 550 nm using a PHERastar plate reader (BMG Labtech, Ortenberg, Germany). The viability of the cells was related to untreated control wells/cells. Live/dead staining was performed using fluorescein diacetate (FDA) to stain living cells and propidium iodide (PI) to stain the dead ones. The cells were seeded and incubated with the supernatant as described before. After 1 day of incubation, the cells were washed 3 times with sterile PBS and then 0.03 mg/mL FDA and 0.02 mg/mL PI were added into each well and incubated for 5 min at 37 °C and 5% CO_2. Imaging was performed using 5x magnification to have a general overview about the cell behavior. 20x magnification was used as well to visualize the change in the morphology of the cells in the bright field channel.

Results of a duplicate of three independent experiments were plotted and illustrated using GraphPad Prism V.9 and presented as mean values ± standard deviation. The statistical analysis of variance was performed with ANOVA followed by Dunnett's multiple comparisons test. Statistical significance was calculated by comparing non-treated cell *vs* treated cells and a *P* value less than 0.05 was significant. For image illustration purposes, the brightness and contrast were optimized for all images based on the values of control (no treatment) images for each channel.

***In vivo Galleria mellonella* virulence assay.** *G. mellonella* larvae were purchased from BioSystems Technology (Exeter, United Kingdom), stored at 8 °C in the dark and used within 2 weeks. Prior to injection, larvae were immobilized by incubation for 10–15 min on ice. Then, the injection was performed using an LA120 syringe pump (Landgraf Laborsysteme, Langenhagen, Germany) supplied with a 1 mL syringe (B. Braun, Melsungen, Germany) and Sterican 0.30 × 12 mm, 30G × 1.5 sterile needles (B. Braun). The larvae were injected with 10 μL of sample into the right proleg. The larvae were classified into various groups based on the applied treatment. Two negative control groups supplemented with no injection to control the quality of the larvae and a buffer control group injected with sterile PBS were included. A positive control group was also included, and the larvae were administered with 50% wt PA14 supernatant. To test the anti-virulence effect of LasB inhibitors, a mixture of 50% wt PA14 supernatant, LasB inhibitor and 300 μM TCEP were incubated for 30 min at 37 °C and injected into the larvae. A group of larvae injected with 50% Δ*lasB* PA14 supernatant was also involved. All groups were incubated at 37 °C and inspected once per day for 4 days post-treatment and to record mortality. The larvae were considered dead if they are black and do not move when stimulated by contact with the forceps. The survival analysis was performed using GraphPad Prism V9, data were plotted using the Kaplan–Meier method and statistical significance between groups was calculated with log-rank test. The data of three independent experiments were combined and plotted in the survival curve, thirty larvae in total were included for each condition.

Synthesis of Intermediates and Final Compounds

General procedure A: Synthesis of chloro acid derivatives 6–10 from amino acid

Amino acid (1.0 eq) was dissolved in 6 N HCl (2 mL/mmol or until mostly dissolved) under nitrogen atmosphere and cooled to –5 °C. $NaNO_2$ (1.5–2.5 eq) was dissolved in water (0.3 mL/mmol amino acid) and added dropwise slowly. The mixture was stirred overnight while warming to r.t. The reaction mixture was extracted with EtOAc/THF (3:1). Combined organic extracts were washed with saturated aq. NaCl solution, dried over anh. Na_2SO_4 and

filtered. The solvent was removed under reduced pressure to obtain the product. The crude is used in the next steps without further purification.

General procedure B: Synthesis of derivatives 11a–17a using thionyl chloride

The acid (1.0 eq), $SOCl_2$ (2.0 eq) and a few drops of DMF were heated to 70 °C for 1 h. The cooled mixture was added dropwise to a solution of the corresponding aniline (1.1 eq) in DMF (1 mL/mmol) at 0 °C. The mixture was stirred overnight at r.t. The reaction was quenched with water and extracted with EtOAc (3×). Combined organic extracts were washed with saturated aq. NaCl solution, dried over anh. Na_2SO_4 and filtered. The solvent was removed under reduced pressure to obtain the crude product. The purification was done by column chromatography or flash chromatography.

General Procedure B-1: Synthesis of coupling derivatives 18a, 23a–25a using ethylchloroformate as coupling reagent

The acid (1.2 eq) was dissolved in THF and cooled in an ice-bath. Et_3N (1.2 eq) was added, followed by addition of $ClCO_2Et$ (1.3 eq). After 5 minutes, ice-bath was removed, and reaction was stirred at r.t. for 30 minutes. The corresponding amine (1.0 eq) was slowly added. The reaction was monitored using TLC or LC-MS. After the reaction was completed, volatiles were evaporated under reduced pressure and crude product was purified using column chromatography.

General Procedure B-2: Synthesis of coupling derivatives 17a, 19a–22a and 26a using HATU as coupling reagent

The acid (1.5 eq) was dissolved in DCM (10 mL) at r.t. and to this DIEA (1.5 eq) and HATU (1.5 eq) were added. The corresponding aniline (1 eq) was then added to this mixture and the reaction was monitored by LC-MS. The reaction is extracted with saturated aq. NaCl solution (1×) then dried over anh. Na_2SO_4 and filtered. The crude was purified using reverse phase flash chromatography (H_2O+0.1 %FA/ACN+0.1%FA 95:5 → 5:95).

General procedure C: Protection of hydroxyl group in derivatives 13b, 15b and 16b

The amide (1.0 eq), Et_3N (2.0 eq) and 4-dimethylaminopyridine (0.03 eq) were dissolved in DCM (5 mL/mmol) and cooled to 0 °C. Acetic anhydride (2.0 eq) was added dropwise. The solution was warmed to r.t. and stirred for 30 min. The reaction was washed with DCM, washed with saturated aq. NaCl solution, and dried over anh. Na_2SO_4. The solvent was removed under reduced pressure to obtain the crude product.

General procedure D: Synthesis of thioacetate derivatives 11b, 12b, 13c, 14b, 15c, 16c, and 17b–26b

The corresponding chloro derivative (1.0 eq) was dissolved in acetone under argon atmosphere. To this solution, CH_3COSK (1.5–3.0 eq) was added and the reaction was stirred for 2–6 h at r.t. It was monitored by TLC or LC-MS. The reaction was quenched with water and extracted with EtOAc (3×). Combined organic extracts were washed with saturated aq. NaCl solution, dried over anh. Na_2SO_4 and filtered. The solvent was removed under reduced pressure to obtain the crude product. The purification was done by flash chromatography.

General procedure E: Hydrolysis of thioacetate for derivatives 11–26

Thioacetate (1.0 eq) was dissolved in methanol (5 mL/mmol) under argon atmosphere and 2 M aqueous NaOH solution (2.0 eq) or solid NaOH (3.0 eq) was added. The reaction was stirred 1–3 h at r.t. before quenching with 1 M HCl. Reaction was extracted with EtOAc and washed with 0.5 M HCl. Combined organic extracts were washed with saturated aqueous NaCl solution

and dried over anh. Na_2SO_4 and filtered. The solvent was removed under reduced pressure to obtain the crude product. The purification was done by column chromatography or preparative HPLC (H_2O+0.05%FA/ACN+0.05%FA, 95:5 → 5:95). For more polar compounds, instead of quenching the reaction with 1 M HCl, pH was adjusted to acidic using Amberlite IR-120. After filtration, Amberlite was washed with MeOH (3×), the solvent was evaporated, and the product was purified using preparative HPLC (H_2O+0.05%FA/ACN+0.05%FA, 95:5 → 5:95). For compounds **21** and **22**, thioacetate (1.0 eq) was dissolved in methanol (5 mL/mmol) under argon atmosphere and acetyl chloride (15 eq) was added dropwise over 10 hours. The mixture was stirred at room temperature for 30–40 hours and carefully monitored by LC-MS. Once the conversion was complete, the solvent was removed under reduced pressure to obtain the crude product. Purification was done by preparative HPLC (H_2O+0.05%FA/ACN+0.05%FA, 95:5 → 5:95).

2-Chloro-3-phenylpropanoic acid (6).

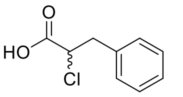

Compound **6** was prepared according to general procedure **A**, using DL-phenylalanine (1 g, 6.0 mmol) and $NaNO_2$ (1.46 g, 21.2 mmol). The crude product was obtained as yellow oil and used without further purification (1.05 g, 94%). ^{1}H NMR (500 MHz, $CDCl_3$) δ ppm: 7.37–7.24 (m, 5H), 4.51 (dd, *J* = 7.8, 6.9 Hz, 1H), 3.42 (dd, *J* = 14.0, 6.7 Hz, 1H), 3.21 (dd, *J* = 14.1, 7.9 Hz, 1H). MS (ESI$^-$) *m/z* 183.25 [M–H]$^-$, 147.23 [M–H–HCl]$^-$. The signals correspond to those reported in literature.[13]

2-Chloro-3-(4-hydroxyphenyl)propanoic acid (7).

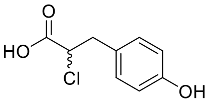

Compound **7** was prepared according to general procedure **A**, using DL-tyrosine (500 mg, 2.76 mmol) and $NaNO_2$ (286 mg, 4.10 mmol). The product was obtained as off-yellow oil and used without further purification (385 mg, 70%). ^{1}H NMR (500 MHz, $CDCl_3$) δ ppm: 7.10 (m, 2H), 6.77 (m, 2H), 4.44 (t, *J* = 7.3 Hz, 1H), 3.35–3.27 (m, 1H), 3.14 (dd, *J* = 14.1, 7.2 Hz, 1H). MS (ESI$^-$) *m/z* 199.22 [M–H]$^-$, 163.20 [M–H–HCl]$^-$. The signals correspond to those reported in literature.[14]

2-Chloro-3-(4-hydroxy-3-nitrophenyl)propanoic acid (**8**).

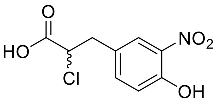

Compound **8** was prepared according to general procedure **A**, using 3-nitro-DL-tyrosine (500 mg, 2.76 mmol) and $NaNO_2$ (665 mg, 3.5 mmol). The product was obtained as brown oil and used without further purification (344 mg, 63%). ^{1}H NMR (500 MHz, $CDCl_3$) δ ppm: 10.53 (br s, 1H), 8.02 (d, *J* = 2.0 Hz, 1H), 7.50 (dd, *J* = 8.5, 2.1 Hz, 1H), 7.14 (d, *J* = 8.7 Hz, 1H), 4.49 (dd, *J* = 7.5, 6.4 Hz, 1H), 3.39 (dd, *J* = 14.4, 6.2 Hz, 1H), 3.22 (dd, *J* = 14.4, 7.9 Hz, 1H). MS (ESI$^-$) *m/z* 244.19 [M–H]$^-$, 208.22 [M–H–HCl]$^-$.

2-Chloro-3-(*p*-tolyl)propanoic acid (9).

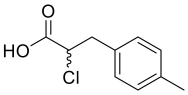

Compound **9** was prepared according to general procedure **A**, using 4-methyl-DL-phenylalanine (200 mg, 1.12 mmol) and $NaNO_2$ (192 mg, 2.79 mmol). The crude product was obtained as yellow oil and used without further purification (206 mg, 93%). ^{1}H NMR (500 MHz, CD_3OD) δ ppm: 7.25–7.07 (m, 4H), 4.21 (dd, *J* = 7.8, 5.4 Hz, 1H), 3.27 (dd, *J* = 14.6, 5.3 Hz, 1H), 3.13 (dd, *J* = 14.5, 7.8 Hz, 1H), 2.33 (s, 3H). MS (ESI$^-$) *m/z* 197.15 [M–H]$^-$161.15 [M–H–HCl]$^-$. The signals correspond to those reported in literature.[15]

2-Chloro-3-(4-chlorophenyl)propanoic acid (10).

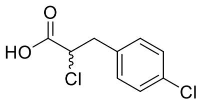

Compound **10** was prepared according to general procedure **A**, using 4-chloro-DL-phenylalanine (500 mg, 2.51 mmol) and $NaNO_2$ (605 mg, 8.77 mmol). The crude product was obtained as yellow oil and used without further purification (550 mg, 100%). ^{1}H NMR (500 MHz, CDCl3) δ ppm: 7.43–7.11 (m, 4H), 4.51 (m, 1H), 3.39 (dd, J = 14.1, 6.9 Hz, 1H), 3.21 (dd, J = 14.0, 7.7 Hz, 1H). MS (ESI^{-}) *m/z* 218.05 [M–H]$^{-}$. The signals correspond to those reported in literature.[16]

2-Chloro-*N*-(4-nitrophenyl)-3-phenylpropanamide. (11a).

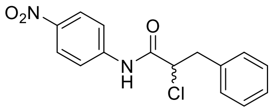

Compound **11a** was prepared according to general procedure **B**, using compound **6** (200 mg, 1.08 mmol), $SOCl_2$ (157 µL, 2.17 mmol) and 4-nitroaniline (164 mg, 1.19 mmol). Purification was done by flash chromatography (Hex/EtOAc, 100:0 to 0:100). The product was obtained as yellow oil (198 mg, 60%). ^{1}H NMR (500 MHz, $CDCl_3$) δ ppm: 8.33 (br s, 1H), 8.26–8.21 (m, 2H), 7.70–7.65 (m, 2H), 7.36–7.24 (m, 5H), 4.73 (dd, *J* = 7.5, 4.6 Hz, 1H), 3.53 (dd, *J* = 14.3, 4.6 Hz, 1H), 3.34 (dd, *J* = 14.3, 7.5 Hz, 1H). MS (ESI^{+}) *m/z* 305.11 [M+H]$^{+}$. The signals correspond to those reported in literature.[17]

2-Chloro-*N*-(4-methoxyphenyl)-3-phenylpropanamide (12a).

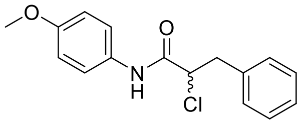

Compound **12a** was prepared according to general procedure **B**, using compound **6** (200 mg, 1.08 mmol), $SOCl_2$ (157 µL, 2.17 mmol) and *p*-anisidine (147 mg, 1.19 mmol). Purification was done by flash chromatography (Hex/EtOAc, 100:0 to 0:100). The product was obtained as green solid (234 mg, 75%) ^{1}H NMR (500 MHz, $CDCl_3$) δ ppm: 8.01 (br s, 1H), 7.38–7.24 (m, 7H), 6.90–6.86 (m, 2H), 4.71 (dd, *J* = 7.8, 4.4 Hz, 1H), 3.81 (s, 3H), 3.52 (dd, *J* = 14.3, 4.4 Hz, 1H), 3.32 (dd, *J* = 14.3, 7.6 Hz, 1H). MS (ESI^{+}) *m/z* 290.04 [M+H]$^{+}$. The signals correspond to those described previously.[18]

2-Chloro-*N*-(4-hydroxyphenyl)-3-phenylpropanamide (13a).

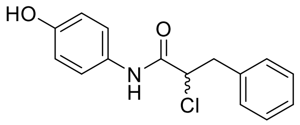

Compound **13a** was prepared according to general procedure **B**, using compound **6** (300 mg, 1.62 mmol), $SOCl_2$ (239 µL, 3.25 mmol) and 4-aminophenol (195 mg, 1.79 mmol). Purification was done by flash chromatography (Hex/EtOAc, 100:0 to 0:100). The final product was obtained as yellow solid (264 mg, 59%). ^{1}H NMR (500 MHz, acetone-d_6) δ ppm: 9.19 (br s, 1H), 8.24 (s, 1H), 7.41 (d, *J* = 8.8 Hz, 2H), 7.33–7.26 (m, 4H), 7.24–7.20 (m, 1H), 6.76 (d, *J* = 8.8 Hz, 2H), 4.65 (t, *J* = 7.3 Hz, 1H), 3.47 (dd, *J* = 13.7, 7.3 Hz, 1H), 3.16 (dd, *J* = 13.8, 7.2 Hz, 1H). MS (ESI^{+}) *m/z* 276.00 [M+H]$^{+}$.

2-Chloro-*N*-phenyl-3-(p-tolyl)propenamide (14a).

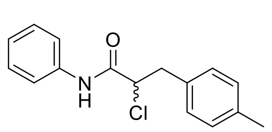

Compound **14a** was prepared according to general procedure **B**, using compound **9** (335 mg, 1.68 mmol), $SOCl_2$ (244 µL, 3.36 mmol) and aniline (196 µL, 1.85 mmol). Purification was done by flash chromatography (Hex/EtOAc, 100:0 to 0:100). The product was obtained as yellow solid (169 mg, 37%). ^{1}H NMR (500 MHz, $CDCl_3$) δ ppm: 8.07 (br s, 1H), 7.49 (br d, *J* = 8.2 Hz, 2H), 7.36 (t, *J* = 7.8 Hz, 2H), 7.20–7.11 (m, 5H), 4.67 (dd, *J* = 7.8, 4.4 Hz, 1H), 3.50 (dd, *J* = 14.3, 4.4 Hz, 1H), 3.28 (dd, *J* = 14.3, 7.8 Hz, 1H), 2.34 (s, 3H). ^{13}C NMR (126 MHz, $CDCl_3$) δ ppm: 166.0, 136.7, 136.5, 132.5, 129.3, 128.9, 128.8, 124.9, 120.0, 61.8, 40.8, 20.8. MS (ESI^{+}) *m/z* 274.04 [M+H]$^{+}$.

2-Chloro-3-(4-hydroxyphenyl)-*N*-phenylpropanamide (15a).

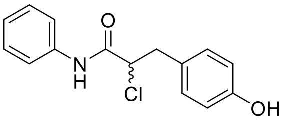

Compound **15a** was prepared according to general procedure **B**, using compound **7** (283 mg, 1.4 mmol), $SOCl_2$ (205 µL, 2.8 mmol) and aniline (142 µL, 1.55 mmol). Purification was done by flash chromatography (Hex/EtOAc, 100:0 to 0:100). The product was obtained as yellow oil (194 mg, 50%). ^{1}H NMR (500 MHz, acetone-d_6) δ ppm: 9.36 (br s, 1H), 8.22 (s, 1H), 7.62 (d, *J* = 8.1 Hz, 2H), 7.30 (t, *J* = 7.8 Hz, 2H), 7.14 (d, *J* = 8.4 Hz, 2H), 7.09 (t, *J* = 7.0 Hz, 1H), 6.75 (d, *J* = 8.4 Hz, 2H), 4.61 (t, *J* = 7.3 Hz, 1H), 3.39 (dd, *J* = 13.9, 7.8 Hz, 1H), 3.08 (dd, *J* = 13.9, 6.9 Hz, 1H). ^{13}C NMR (126 MHz, acetone-*d*$_6$) δ ppm: 167.4. 157.4, 139.5, 131.5, 129.7, 128.4, 125.0, 120.6, 116.2, 60.9, 40.9. MS (ESI$^+$) *m/z* 276.08 [M+H]$^+$.

2-Chloro-3-(4-hydroxy-3-nitrophenyl)-*N*-phenylpropanamide (16a).

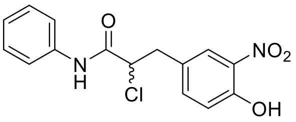

Compound **16a** was prepared according to general procedure **B**, using compound **8** (300 mg, 1.22 mmol), $SOCl_2$ (218 µL, 3.0 mmol) and aniline (150 µL, 1.65 mmol). Purification was done by flash chromatography (Hex/EtOAc, 100:0 to 0:100). The product was obtained as yellow solid (189 mg, 48%). ^{1}H NMR (500 MHz, $CDCl_3$) δ ppm: 10.54 (s, 1H), 8.15 (br s, 1H), 8.07 (d, *J* = 2.1 Hz, 1H), 7.52 (dd, *J* = 8.5, 2.1 Hz, 1H), 7.49 (d, *J* = 7.8 Hz, 2H), 7.37 (t, *J* = 7.9 Hz, 2H), 7.12 (d, *J* = 8.7 Hz, 1H), 7.19 (t, *J* = 7.9 Hz, 1H), 4.69 (dd, *J* = 7.4, 4.3 Hz, 1H), 3.50 (dd, *J* = 14.6, 4.7 Hz, 1H), 3.39 (dd, *J* = 14.6, 7.5 Hz, 1H). ^{13}C NMR (126 MHz, $CDCl_3$) δ ppm: 165.1, 154.0, 138.8, 136.2, 133.1, 128.9, 127.9, 125.7, 125.2, 120.0, 119.9, 60.8, 39.6. MS (ESI$^+$) *m/z* 321.16 [M+H]$^+$.

2-Chloro-3-(4-chlorophenyl)-*N*-phenylpropanamide (17a).

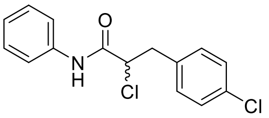

Compound **17a** was prepared according to the general procedure **B-2**, using compound **10** (550 mg, 2.51 mmol), aniline (257 mg, 2.76 mmol), DIEA (641 µL, 3.77 mmol), HATU (1.43 g, 3.77 mmol) in DCM (17 mL). The product was purified by flash column chromatography (Hex/EtOAc, 100:0 to 0:100). The final product was obtained as yellow oil (420 mg, 57%). ^{1}H NMR (500 MHz, DMSO) δ ppm: 10.31 (s, 1H), 7.55 (d, *J* = 7.7 Hz, 2H), 7.39–7.29 (m, 6H), 7.12–7.06 (m, 1H), 4.72 (t, *J* = 7.4 Hz, 1H), 4.06 (s, 1H), 3.38 (dd, *J* = 13.9, 7.8 Hz, 1H), 3.13 (dd, *J* = 13.8, 7.8 Hz, 1H). ^{13}C NMR (126 MHz, $CDCl_3$) δ ppm: 166.2, 138.2, 135.8, 131.7, 131.2, 128.9, 128.4, 124.1, 119.5, 59.2, 40.0 MS (ESI$^+$) *m/z* 294.04 [M+H]$^+$.

2-Chloro-3-phenyl-*N*-(thiazol-2-yl)propenamide (18a).

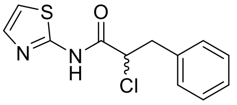

Compound **18a** was synthesized according to the general procedure **B-1**, using compound **6** (1.15 g, 6.23 mmol), 2-aminothiazole (517 mg, 5.17 mmol), Et_3N (875 µL, 6.23 mmol) and $ClCO_2Et$ (652 µL, 6.86 mmol) in THF (61 mL). The final product was purified using column chromatography (Hex/EtOAc, 4:1). The final product was obtained as yellow oil (459 mg, 27%). ^{1}H NMR (500 MHz, acetone-*d*$_6$) δ ppm: 11.15 (s, 1H), 7.49–7.40 (t, *J* = 3.1 Hz, 1H), 7.38–7.05 (m, 6H), 4.95 (m, 1H), 3.54 (m, 1H), 3.26 (m, 1H).^{13}C NMR (126 MHz, acetone-*d*$_6$) δ ppm: 167.4, 138.9, 138.8, 137.3, 130.3, 129.3, 128.0, 114.9, 58.9, 41.1. MS (ESI$^+$) *m/z* 266.84 [M+H]$^+$.

Methyl 2-(2-chloro-3-phenylpropanamido)thiophene-3-carboxylate (19a).

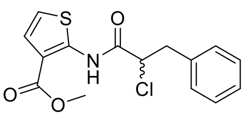

Compound **19a** was prepared according to the general procedure **B-2**, using compound **6** (658 mg, 3.56 mmol), methyl 3-amino-thiophene-2-carboxylate (372 mg, 2.37 mmol), DIEA (619 µL, 3.56 mmol), HATU (1.35 mg, 3.56 mmol) in DCM (25 mL). The product was purified by reverse phase flash column chromatography. The final product was obtained as off-white solid (200 mg, 17%). ^{1}H NMR (500 MHz, DMSO-*d*$_6$) δ ppm: 10.66 (s, 1H), 7.94 (q, *J* = 5.4 Hz, 2H), 7.33–7.27 (m, 4H), 7.27–7.21 (m, 1H), 5.21 (dd, *J* = 8.4, 5.5 Hz, 1H), 3.83 (s, 3H), 3.43 (dd, *J*

= 14.3, 5.5 Hz, 1H), 3.18 (dd, *J* = 14.3, 8.4 Hz, 1H).^{13}C NMR (126 MHz, $CDCl_3$) δ ppm: 166.1, 163.3, 142.5, 136.4, 133.4, 129.4, 128.4, 127.0, 122.0, 111.9, 60.1, 52.3, 40.2. MS (ESI$^+$) *m/z* 324.03 [M+H]$^+$.

2-Chloro-3-phenyl-*N*-(pyridin-3-yl)propenamide (20a).

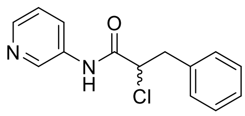

Compound **20a** was prepared according to the general procedure **B-2**, using compound **6** (422 mg, 2.28 mmol), 3-amino-pyridine (143 mg, 1.52 mmol), DIEA (396.9 µL, 2.28 mmol), HATU (866 mg, 2.28 mmol) in DCM (20 mL). The product was purified by reverse phase flash column chromatography. The final product was obtained as yellow oil (312 mg, 52%). ^{1}H NMR (500 MHz, $CDCl_3$) δ ppm: 8.57 (s, 1H), 8.45–8.31 (m, 2H), 8.18 (d, *J* = 8.1 Hz, 1H), 7.37 (dd, *J* = 8.1, 4.6 Hz, 1H), 7.34–7.26 (m, 4H), 4.71 (dd, *J* = 7.5, 4.9 Hz, 1H), 4.06 (s, 1H), 3.51 (dd, *J* = 14.3, 4.8 Hz, 1H), 3.31 (dd, *J* = 14.3, 7.6 Hz, 1H). ^{13}C NMR (126 MHz, $CDCl_3$) δ ppm: 167.2, 144.9, 140.6, 135.7, 134.4, 129.8, 128.8, 128.7, 127.6, 124.4, 61.3, 41.3. MS (ESI$^+$) *m/z* 261.07 [M+H]$^+$.

2-Chloro-3-phenyl-*N*-(pyridin-2-yl)propenamide (21a).

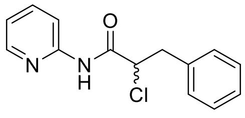

Compound **21a** was prepared according to the general procedure **B-2**, using compound **6** (300 mg, 1.61 mmol), 2-amino-pyridine (166 mg, 1.77 mmol), DIEA (328.0 µL, 1.93 mmol), HATU (733 mg, 1.93 mmol) in DCM (10 mL). The product was purified by reverse phase flash column chromatography. The final product was obtained as yellow oil (180 mg, 43%). ^{1}H NMR (500 MHz, MeOD) δ ppm: 8.29 (d, *J* = 4.6 Hz, 1H), 8.07 (d, *J* = 8.3 Hz, 1H), 7.84–7.79 (m, 1H), 7.30 (d, *J* = 4.3 Hz, 4H), 7.26–7.21 (m, 1H), 7.18–7.14 (m, 1H), 4.79 (t, *J* = 7.3, 1H), 4.06 (s, 1H), 3.46 (dd, *J* = 13.8, 7.6 Hz, 1H), 3.22 (dd, *J* = 13.8, 7.6 Hz, 1H). ^{13}C NMR (126 MHz, MEOD) δ ppm: 167.2, 144.9, 140.6, 135.7, 134.4, 129.8, 128.8, 128.7, 127.6, 124.4, 61.3, 41.3. MS (ESI$^+$) *m/z* 261.08 [M+H]$^+$.

2-Chloro-3-phenyl-*N*-(pyridin-4-yl)propanamide (22a).

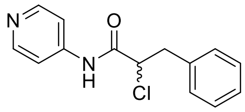

Compound **22a** was prepared according to the general procedure **B-2**, using compound **6** (500 mg, 2.68 mmol), 4-amino-pyridine (277 mg, 2.95 mmol), DIEA (683.0 µL, 4.02 mmol), HATU (1.53 g, 4.02 mmol) in DCM (18 mL). The product was purified by reverse phase flash column chromatography. The final product was obtained as yellow oil (265 mg, 38%). ^{1}H NMR (500 MHz, $CDCl_3$) δ ppm: 8.64 (s, 1H), 8.50 (d, *J* = 5.6 Hz, 2H), 7.57 (d, *J* = 5.7 Hz, 2H), 7.35–7.22 (m, 5H), 4.74–4.68 (m, 1H), 4.06 (s, 1H), 3.51 (dd, *J* = 14.3, 4.9 Hz, 1H), 3.29 (dd, *J* = 14.3, 7.6 Hz, 1H). ^{13}C NMR (126 MHz, $CDCl_3$) δ ppm: 167.4, 149.2, 145.6, 135.5, 129.9, 129.7, 128.7, 127.7, 114.2, 61.2, 41.2. MS (ESI$^+$) *m/z* 261.08 [M+H]$^+$.

***N*-(Benzo[d]thiazol-2-yl)-2-chloro-3-phenylpropanamide (23a).**

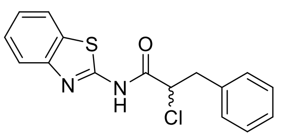

Compound **23a** was synthesized according to the general procedure **B-1**, using compound **6** (626 mg, 3.39 mmol), 2-aminobenzothiazole (422 mg, 2.81 mmol), Et_3N (476 µL, 3.39 mmol) and $ClCO_2Et$ (355 µL, 3.72 mmol) in THF (33 mL). The final product was purified using flash chromatography (DCM/MeOH, 100:0 to 95:5). Final product was obtained as off-white oil (324 mg, 30%). ^{1}H NMR (500 MHz, $CDCl_3$) δ ppm: 7.86 (d, *J* = 7.9 Hz, 1H), 7.78 (m, 1H), 7.47 (t, *J* = 7.7 Hz, 1H), 7.36 (t, *J* = 7.6 Hz, 1H), 7.32–7.23 (m, 3H), 7.21–7.18 (m, 2H), 4.76–4.70 (m, 1H), 3.56–3.51 (m, 1H), 3.29 (dd, *J* = 14.4, 7.8 Hz, 1H). ^{13}C NMR (126 MHz, $CDCl_3$) δ ppm: 167.1, 157.3, 148.2, 135.3, 132.3, 129.6, 128.8, 127.7, 127.72, 126.7, 124.6, 121.7, 121.3, 60.3, 41.2. MS (ESI$^+$) *m/z* 316.98 [M+H]$^+$.

2-Chloro-*N*-(6-methoxybenzo[d]thiazol-2-yl)-3-phenylpropanamide (24a).

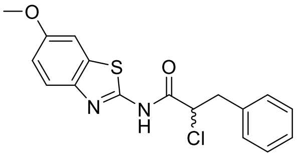

Compound **24a** was synthesized according to the general procedure **B-1**, using compound **6** (675 mg, 3.65 mmol), 2-amino-6-methoxybenzothiazole (545 mg, 3.03 mmol), Et_3N (510 µL, 3.65 mmol) and $ClCO_2Et$ (380 µL, 4.01 mmol) in THF (36 mL). The product was purified using column chromatography (DCM/Hex, 3:2). The final product was obtained as yellow oil (450 mg, 35%). ^1H NMR (500 MHz, acetone-d_6) δ ppm 11.36 (s, 1H), 7.62 (d, J = 8.8 Hz 1H), 7.53 (d, J = 2.6 Hz, 1H), 7.38–7.28 (m, 4H), 7.26–7.22 (m, 1H), 7.04 (dd, J = 8.9, 2.6 Hz, 1H), 4.98 (t, J = 7.4 Hz, 1H), 3.86 (s, 3H), 3.56 (dd, J = 13.9, 7.1 Hz, 1H), 3.27 (dd, J = 13.9, 7.7 Hz, 1H). ^{13}C NMR (126 MHz, acetone-d_6) δ ppm: 168.0, 157.9, 155.8 143.9, 137.3, 134.4, 130.3, 129.4, 128.0, 122.5, 116.1, 105.0, 59.1, 56.1, 41.0. MS (ESI$^+$) *m/z* 346.88 [M+H]$^+$.

2-Chloro-*N*-(6-chlorobenzo[d]thiazol-2-yl)-3-phenylpropanamide (25a).

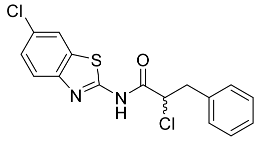

Compound **25a** was prepared according to the general procedure **B-1**, using compound **6** (862 mg, 4.66 mmol), 2-amino-6-chlorobenzothiazole (715 mg, 3.86 mmol), Et_3N (656 µL, 4.66 mmol) and $ClCO_2Et$ (489 µL, 5.13 mmol) in THF (46 mL). The product was purified by flash column chromatography (Hex/EtOAc, 7:3). The final product was obtained as yellow oil (658 mg, 40%). ^1H NMR (500 MHz, $CDCl_3$) δ ppm: 9.66 (s, 1H), 7.82 (d, J = 2.0 Hz, 1H), 7.75–7.67 (m, 1H), 7.45–7.37 (m, 1H), 7.34–7.26 (m, 3H), 7.25–7.20 (m, 2H), 4.77 (dd, J = 7.8, 4.6, Hz, 1H), 3.59–3.50 (m, 1H), 3.32 (dd, J = 14.4, 7.9 Hz, 1H). ^{13}C NMR (126 MHz, $CDCl_3$) δ ppm: 167.1, 157.3, 147.0, 135.1, 133.6, 130.2, 129.7, 128.8, 127.8, 127.4, 122.3, 121.3, 60.4, 41.2. MS (ESI$^+$) *m/z* 350.95 [M+H]$^+$.

***N*-(1*H*-Benzo[d]imidazol-2-yl)-2-chloro-3-phenylpropanamide (26a).**

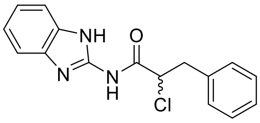

Compound **26a** was prepared according to the general procedure **B-2**, using compound **6** (658 mg, 3.56 mmol), 1*H*-benzo[d]imidazol-2-amine (372 mg, 2.37 mmol), DIEA (619 µL, 3.56 mmol), HATU (1.35 mg, 3.56 mmol) in DCM (25 mL). The product was purified by reverse phase flash column chromatography. The final product was obtained as off-white solid (200 mg, 30%). ^1H NMR (500 MHz, $CDCl_3$) δ ppm: 7.52–7.46 (m, 2H), 7.34–7.26 (m, 2H), 7.25–7.15 (m, 3H), 7.14–7.10 (m, 2H), 4.70 (t, J = 7.1 Hz, 1H), 4.12(br s, 1H), 3.54–3.42 (m, 1H), 3.33–3.16 (m, 1H). ^{13}C NMR (126 MHz, $CDCl_3$) δ ppm: 170.1, 147.1, 135.6, 129.50, 129.5, 128.8, 128.7, 127.63, 127.6, 123.4, 59.5, 41.2. MS (ESI$^+$) *m/z* 300.03 [M+H]$^+$.

***S*-(1-((4-Nitrophenyl)amino)-1-oxo-3-phenylpropan-2-yl) ethanethioate (11b).**

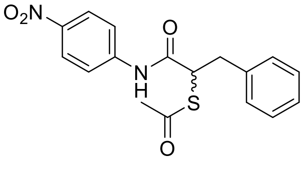

Compound **11b** was prepared according to general procedure **D**, using compound **11a** (190 mg, 0.78 mmol) and potassium thioacetate (134 mg, 1.17 mmol). Purification was done by flash chromatography (Hex/EtOAc, 100:0 to 0:100). The product was obtained as yellow oil (127 mg, 47%). ^1H NMR (500 MHz, $CDCl_3$) δ ppm: 8.45 (br s, 1H), 8.21–8.15 (m, 2H), 7.66–7.61 (m, 2H), 7.33–7.29 (m, 1H), 7.28–7.23 (m, 4H), 4.31 (dd, J = 8.5, 7.1 Hz, 1H), 3.46 (dd, J = 14.2, 8.5 Hz, 1H), 3.01 (dd, J = 14.2, 7.0 Hz, 1H), 2.41 (s, 3H). ^{13}C NMR (126 MHz, $CDCl_3$) δ ppm: 197.9, 168.6, 143.4, 143.1, 136.8, 128.9, 128.4, 126.9, 124.8, 119.0, 48.1, 35.0, 30.2. MS (ESI$^+$) *m/z* 345.11 [M+H]$^+$, 303.03 [M–Ac+2H]$^+$.

***S*-(1-((4-Methoxyphenyl)amino)-1-oxo-3-phenylpropan-2-yl) ethanethioate (12b).**

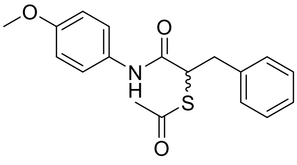

Compound **12b** was prepared according to general procedure **D**, using compound **12a** (230mg, 0.95 mmol) and potassium thioacetate (162 mg, 1.42 mmol). Purification was done by flash chromatography (Hex/EtOAc, 100:0 to 0:100). The product was obtained as yellow

solid (126 mg, 40%). ^{1}H NMR (500 MHz, $CDCl_3$) δ ppm: 7.81 (br s, 1H), 7.37–7.34 (m, 2H), 7.33–7.23 (m, 5H), 6.86–6.81 (m, 2H), 4.28 (dd, *J* = 8.4, 7.2 Hz, 1H), 3.79 (s, 3H), 3.44 (dd, *J* = 14.0, 8.4 Hz, 1H), 3.01 (dd, *J* = 14.1, 7.1 Hz, 1H), 2.38 (s, 3H). ^{13}C NMR (126 MHz, $CDCl_3$) δ ppm: 197.4, 168.3, 156.7, 137.9, 130.9, 129.5, 128.8, 127.2, 114.3, 121.8, 55.7, 48.7, 36.1, 30.7. MS (ESI$^+$) *m/z* 330.08 [M+H]$^+$, 288.08 [M–Ac+2H]$^+$.

4-(2-chloro-3-Phenylpropanamido)phenyl acetate (13b).

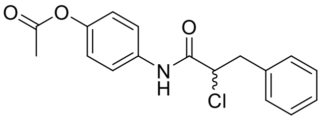

Compound **13b** was prepared according to general procedure **C**, using **13a** (264 mg, 0.96 mmol), Et_3N (266 µL, 1.92 mmol), 4-dimethyl aminopyridine (3.5 mg, 0.03 mmol) and acetic anhydride (181 µL, 1.92 mmol). Purification was done by flash chromatography (Hex/EtOAc, 100:0 to 0:100). The product was obtained as yellow solid (300 mg, 94%). ^{1}H NMR (500 MHz, acetone-d_6) δ ppm: 9.48 (br s, 1H), 7.64 (d, *J* = 8.9 Hz, 2H), 7.31 (m, 2H), 7.28 (m, 2H), 7.23 (m, 1H), 7.07 (d, *J* = 8.9 Hz, 2H), 4.70 (t, *J* = 7.3 Hz, 1H), 3.50 (dd, *J* = 13.9, 7.5 Hz, 1H), 3.19 (dd, *J* = 13.8, 7.2 Hz, 1H), 2.23 (s, 3H). ^{13}C NMR (126 MHz, acetone-d_6) δ ppm: 169.8, 167.3, 148.1, 137.8, 131.0, 130.4, 129.4, 128.0, 124.0, 123.0, 121.4, 60.6, 41.5, 21.0. MS (ESI$^+$) *m/z* 318.07 [M+H]$^+$.

***S*-(1-Oxo-1-(phenylamino)-3-(p-tolyl)propan-2-yl) ethanethioate (14b).**

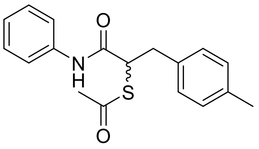

14b was prepared according to general procedure **D**, using **14a** (169 mg, 0.62 mmol) and potassium thioacetate (106 mg, 0.93 mmol). Purification was done via flash chromatography (Hexane/EtOAc, 100:0 to 0:100). The product was obtained as yellow oil (122 mg, 63 %). ^{1}H NMR (500 MHz, $CDCl_3$) δ ppm : 7.96 (br s, 1H), 7.48–7.44 (m, 2H), 7.32–7.28 (m, 2H), 7.18–7.14 (m, 2H), 7.13–7.09 (m, 3H), 4.28 (dd, *J*=8.3, 7.2 Hz, 1H), 3.41 (dd, *J*=14.1, 8.3 Hz, 1H), 2.97 (dd, *J*=14.2, 7.2 Hz, 1H), 2.38 (s, 3H), 2.32 (s, 3H). ^{13}C NMR (126 MHz, $CDCl_3$) δ ppm: 197.4, 168.4, 137.7, 136.6, 134.5, 129.3, 129.1, 129.0, 124.4, 119.8, 48.6, 35.3, 30.4, 21.1. MS (ESI$^+$) *m/z* 314.10 (M+H)$^+$, 272.03 (M–Ac+2H)$^+$.

4-(2-Chloro-3-oxo-3-(phenylamino)propyl)phenyl acetate (15b).

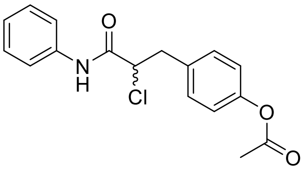

15b was prepared according to general procedure **C**, using **15a** (180 mg, 0.65 mmol), Et_3N (180 µL, 1.30 mmol), 4-dimethylaminopyridine (2.4 mg, 0.02 mmol) and acetic anhydride (123 µL, 1.30 mmol). Purification was done by flash chromatography (Hex/EtOAc, 100:0 to 0:100). The final product was obtained as white solid (114 mg, 55%). ^{1}H NMR (500 MHz, acetone-d_6) δ ppm: 9.44 (br s, 1H), 7.62 (d, *J* = 8.9 Hz, 2H), 7.35 (d, *J* = 8.4 Hz, 2H), 7.31 (t, *J* = 7.9 Hz, 2H), 7.10 (m, 1H), 7.05 (d, *J* = 8.4 Hz, 2H), 4.71 (t, *J* = 7.3 Hz, 1H), 3.50 (dd, *J* = 13.9, 7.5 Hz, 1H), 3.20 (dd, *J* = 13.8, 7.2 Hz, 1H), 2.23 (s, 3H). ^{13}C NMR (126 MHz, acetone-d_6) δ ppm: 169.7, 167.3, 151.1, 139.5, 135.2, 131.4, 129.7, 128.1, 122.7, 120.7, 60.6, 40.8, 21.0. MS (ESI$^+$) *m/z* 318.07 [M+H]$^+$.

4-(2-Chloro-3-oxo-3-(phenylamino)propyl)-2-nitrophenyl acetate (16b).

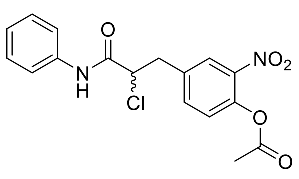

16b was prepared according to general procedure **C**, using **16a** (189 mg, 0.59 mmol), Et_3N (164 µL, 1.18 mmol), 4-dimethylaminopyridine (2.0 mg, 0.02 mmol) and acetic anhydride (112 µL, 1.18 mmol). Purification was done by flash chromatography (Hex/EtOAc, 100:0 to 0:100). The final product was obtained as yellow solid (200 mg, 93%). ^{1}H NMR (500 MHz, acetone-d_6) δ ppm: 9.50 (br s, 1H), 8.15 (d, *J* = 2.0 Hz, 1H), 7.77 (dd, *J* = 8.4, 2.0 Hz, 1H), 7.62 (d, *J* = 8.2 Hz, 2H), 7.36 (d, *J* = 8.2 Hz, 1H), 7.32 (t, *J* = 7.9 Hz, 2H), 7.11 (t, *J* = 7.4 Hz, 1H), 4.84 (t, *J* = 7.2 Hz, 1H), 3.63 (dd, *J* =

14.1, 6.6 Hz, 1H), 3.37 (dd, *J* = 14.1, 7.9 Hz, 1H), 2.31 (s, 3H). ^{13}C NMR (126 MHz, acetone-*d*$_6$) δ ppm: 169.1, 166.9, 143.8, 142.7, 139.3, 137.3, 130.6, 129.8, 127.3, 126.2, 125.2, 120.7, 60.1, 40.2, 20.8. MS (ESI$^+$) *m/z* 362.12 [M+H]$^+$, 321.06 [M–Ac+2H]$^+$.

S-(3-(4-Chlorophenyl)-1-oxo-1-(phenylamino)propan-2-yl) ethanethioate (17b).

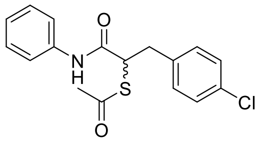

Compound **17b** was prepared according to general procedure **D**, using compound **17a** (200 mg, 0.68 mmol) and potassium thioacetate (233 mg, 2.04 mmol) in acetone (7 mL). Purification was done by column chromatography (Hex/EtOAc, 100:0 to 0:100). The final product was obtained as yellow solid (115 mg, 51%). ^{1}H NMR (500 MHz, $CDCl_3$) δ ppm: 7.99 (s, 1H), 7.44 (d, *J* = 7.7 Hz, 2H), 7.31–7.17 (m, 6H), 7.09 (t, *J* = 7.4 Hz, 1H), 4.27-4.22 (m, 1H), 3.40 (dd, *J* = 14.1, 8.5 Hz, 1H), 2.95 (dd, *J* = 14.2, 7.0 Hz, 1H), 2.36 (s, 3H). ^{13}C NMR (125 MHz, $CDCl_3$) δ ppm: 197.2, 168.2, 137.6, 136.2, 133.0, 130.8, 129.1, 128.8, 124.7, 120.0, 48.3, 35.2, 30.5. MS (ESI$^+$) *m/z* 334.07 [M+H]$^+$.

***S*-(1-Oxo-3-phenyl-1-(thiazol-2-ylamino)propan-2-yl) ethanethioate (18b).**

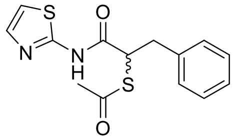

Compound **18b** was prepared according to general procedure **D**, using compound **18a** (336 mg, 1.26 mmol) and potassium thioacetate (215 mg, 1.90 mmol) in acetone (13 mL). Purification was done by flash chromatography (Hex/EtOAc, 3:1). The final product was obtained as white powder (300 mg, 77%). ^{1}H NMR (500 MHz, acetone-*d*$_6$) δ ppm: 11.41 (s, 1H), 7.46–7.37 (m, 1H), 7.3–7.23 (m, 4H), 7.22–7.17 (m, 1H), 7.15 (d, *J* = 3.5 Hz, 1H), 4.66 (dd, *J* = 8.8, 6.7 Hz, 1H), 3.38 (dd, *J* = 13.7, 8.9 Hz, 1H), 3.02 (dd, *J* = 13.7, 6.7 Hz, 1H), 2.05 (d, *J* = 2.2 Hz, 3H). ^{13}C NMR (126 MHz, acetone-*d*$_6$) δ ppm: 194.6, 169.2, 158.5, 138.6 138.4, 130.0, 129.2, 127.7, 114.5, 61.7, 49.1, 38.6. MS (ESI$^+$) *m/z* 306.90 [M+H]$^+$, 264.90 [M–Ac+H]$^+$.

Methyl 2-(2-(acetylthio)-3-phenylpropanamido)thiophene-3-carboxylate (19b).

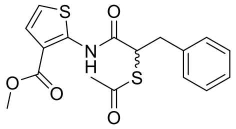

Compound **19b** was prepared according to general procedure **D**, using compound **19a** (172 mg, 0.53 mmol) and potassium thioacetate (112 mg, 0.79 mmol) in acetone (12 mL). Purification was done by column chromatography (Hex/EtOAc, 6:1). The final product was obtained as yellow solid (129 mg, 67%). ^{1}H NMR (500 MHz, $CDCl_3$) δ ppm: 10.46 (s, 1H), 8.10 (d, *J* = 5.5 Hz, 1H), 7.44 (d, *J* = 5.4 Hz, 1H), 7.30–7.26 (m, 2H), 7.26–7.20 (m, 3H), 4.45 (t, *J* = 7.7 Hz, 1H), 3.89 (s, 3H), 3.43 (dd, *J* = 14.2, 7.6 Hz, 1H), 3.07 (dd, *J* = 14.2, 7.7 Hz, 1H), 2.37 (s, 3H). ^{13}C NMR (126 MHz, $CDCl_3$) δ ppm: 194.6, 168.3, 164.4, 143.9, 137.5, 131.5, 129.3, 128.7, 127.1, 122.7, 111.5, 52.2, 49.1, 36.8, 30.5. MS (ESI$^+$) *m/z* 364.05 [M+H]$^+$, 322.03 [M–Ac+H]$^+$.

***S*-(1-Oxo-3-phenyl-1-(pyridin-3-ylamino)propan-2-yl) ethanethioate (20b).**

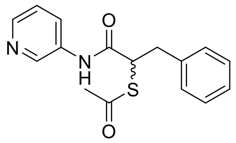

Compound **20b** was prepared according to general procedure **D**, using compound **20a** (326 mg, 1.25 mmol) and potassium thioacetate (264 mg, 1.88 mmol) in acetone (12 mL). Purification was done by column chromatography (DCM/MeOH, 98:2). The final product was obtained as yellow solid (179 mg, 48%). ^{1}H NMR (500 MHz, $CDCl_3$) δ ppm: 8.59 (s, 1H), 8.53–8.21 (m, 2H), 8.17 (d, *J* = 8.5 Hz, 1H), 7.39–7.27 (m, 5H), 7.25–7.20 (m, 1H), 4.37–4.30 (m, 1H), 3.43 (dd, *J* = 14.1, 8.5 Hz, 1H), 3.01 (dd, *J* = 14.1, 7.0 Hz, 1H), 2.39 (s, 3H). ^{13}C NMR (126 MHz, $CDCl_3$) δ ppm: 197.5, 169.3, 144.0, 140.0, 137.4, 129.8, 129.3, 128.8, 128.7, 128.4, 127.3, 124.3, 48.5, 35.9, 30.6. MS (ESI$^+$) *m/z* 301.06 [M+H]$^+$, 260.98 [M–Ac+2H]$^+$.

***S*-(1-Oxo-3-phenyl-1-(pyridin-2-ylamino)propan-2-yl) ethanethioate (21b).**

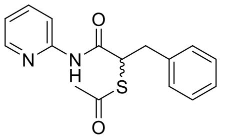

Compound **21b** was prepared according to general procedure **D**, using compound **21a** (170 mg, 0.65 mmol) and potassium thioacetate (115 mg, 1.01 mmol) in acetone (5 mL). Purification was done by column chromatography (DCM/MeOH, 98:2). The final product was obtained as yellow solid (179 mg, 48%). ^{1}H NMR (500 MHz, $CDCl_3$) δ ppm: 8.59 (s, 1H), 8.53–8.21 (m, 2H), 8.17 (d, *J* = 8.5 Hz, 1H), 7.39–7.27 (m, 5H), 7.25–7.20 (m, 1H), 4.37–4.30 (m, 1H), 3.43 (dd, *J* = 14.1, 8.5 Hz, 1H), 3.01 (dd, *J* = 14.1, 7.0 Hz, 1H), 2.39 (s, 3H). ^{13}C NMR (126 MHz, $CDCl_3$) δ ppm: 197.5, 169.3, 144.0, 140.0, 137.4, 129.8, 129.3, 128.8, 128.7, 128.4, 127.3, 124.3, 48.5, 35.9, 30.6. MS (ESI$^+$) *m/z* 301.09 $[M+H]^+$, 260.98 $[M–Ac+2H]^+$.

S-(1-Oxo-3-phenyl-1-(pyridin-4-ylamino)propan-2-yl) ethanethioate (22b).

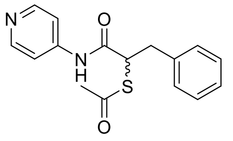

Compound **22b** was prepared according to general procedure **D**, using compound **22a** (120 mg, 0.46 mmol) and potassium thioacetate (158 mg, 1.38 mmol) in acetone (5 mL). Purification was done by column chromatography (DCM/MeOH, 98:2). The final product was obtained as yellow solid (62 mg, 45%). ^{1}H NMR (500 MHz, $CDCl_3$) δ ppm: 8.42 (d, *J* = 3.7 Hz, 2H), 7.44 (d, *J* = 6.4 Hz, 1H), 7.31–7.20 (m, 5H), 4.34–4.29 (m, 1H), 3.41 (dd, *J* = 14.1, 8.5 Hz, 1H), 2.98 (dd, *J* = 14.1, 7.0 Hz, 1H), 2.37 (s, 3H). ^{13}C NMR (126 MHz, $CDCl_3$) δ ppm: 197.5, 169.3, 149.9, 145.3, 137.4, 129.6, 129.2, 128.7, 127.2, 113.9, 48.5, 35.7, 30.4. MS (ESI$^+$) *m/z* 301.09 $[M+H]^+$.

***S*-(1-(Benzo[d]thiazol-2-ylamino)-1-oxo-3-phenylpropan-2-yl) ethanethioate (23b).**

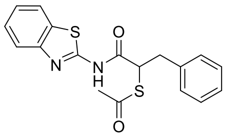

Compound **23b** was prepared according to general procedure **D**, using compound **23a** (323 mg, 1.02 mmol) and potassium thioacetate (174 mg, 1.53 mmol) in acetone (10 mL). Purification was done by flash chromatography (Hex/DCM, 100:0 to 0:100). The final product was obtained as yellow solid (257, 71%). ^{1}H NMR (500 MHz, acetone-d_6) δ ppm: 11.24 (s, 1H), 8.02–7.88 (m, 1H), 7.69 (d, *J* = 8.1 Hz, 1H), 7.43 (m, 1H), 7.35–7.26 (m, 5H), 7.24–7.17 (m, 1H), 4.72 (dd, *J* = 8.7, 6.9 Hz, 1H), 3.41 (dd, *J* = 13.8, 8.7 Hz, 1H), 3.06 (dd, *J* = 13.8, 6.9 Hz, 1H), 2.36 (s, 3H). ^{13}C NMR (126 MHz, $CDCl_3$) δ ppm: 196.2, 169.1, 157.6, 148.4, 136.9, 132.3, 129.3, 128.8, 127.4, 126.5, 124.3, 121.5, 121.2, 47.9, 35.9, 30.5. MS (ESI$^+$) *m/z* 357.01 $[M+H]^+$, 314.90 $[M–Ac+H]^+$.

***S*-(1-((6-Methoxybenzo[d]thiazol-2-yl)amino)-1-oxo-3-phenylpropan-2-yl) ethanethioate (24b).**

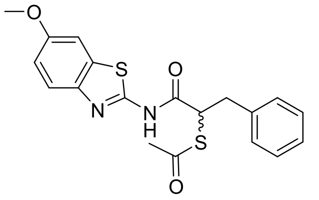

Compound **24b** was prepared according to general procedure **D**, using compound **24a** (377 mg, 1.08 mmol) and potassium thioacetate (186 mg, 1.63 mmol) in acetone (10 mL). Purification was done by flash chromatography (Hex/DCM, 100:0 to 0:100). The final product was obtained as yellow solid (250 mg, 59%). ^{1}H NMR (500 MHz, $CDCl_3$) δ ppm: 7.64 (d, *J* = 8.9 Hz, 1H), 7.32–7.26 (m, 3H), 7.26–7.18 (m, 3H), 7.03 (dd, *J* = 8.9, 2.5 Hz, 1H), 4.44 (t, *J* = 7.7 Hz, 1H), 3.88–3.85 (m, 3H), 3.46 (dd, *J* = 14.2, 7.9 Hz, 1H), 3.06 (dd, *J* = 14.2, 7.6 Hz, 1H), 2.38 (s, 3H). ^{13}C NMR (126 MHz, $CDCl_3$) δ ppm: 196.3, 168.8, 157.04 155.5, 142.5, 136.9, 133.5, 129.3, 128.82, 127.4, 121.8, 115.5, 104.2, 56.0, 47.8, 35.8, 30.5. MS (ESI$^+$) *m/z* 386.88 $[M+H]^+$, 345.00 $[M–Ac+H]^+$.

***S*-(1-((6-Chlorobenzo[d]thiazol-2-yl)amino)-1-oxo-3-phenylpropan-2-yl) ethanethioate (25b).**

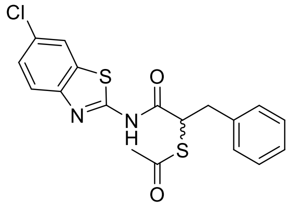

Compound **25b** was prepared according to general procedure **D**, using compound **25a** (658 mg, 1.87 mmol) and potassium thioacetate (325 mg, 2.80 mmol) in acetone (5 mL). Purification was done by flash chromatography (Hex/EtOAc, 7:3). The final product was obtained as yellow solid (300 mg, 41%). ^{1}H NMR (500 MHz, $CDCl_3$) δ ppm: 9.61 (s, 1H), 7.79 (d, J = 2.1 Hz, 1H), 7.69 (d, J = 8.6 Hz, 1H), 7.40 (dd, J = 8.7, 2.1 Hz, 1H), 7.35–7.29 (m, 2H), 7.25–7.22 (m, 3H), 4.45 (t, J = 7.7 Hz, 1H), 3.49 (dd, J = 14.2, 8.0 Hz, 1H), 3.08 (dd, J = 14.2, 7.5 Hz, 1H), 2.42 (s, 3H). ^{13}C NMR (126 MHz, $CDCl_3$) δ ppm: 196.8, 171.3, 169.0, 157.4, 147.3, 136.8, 129.3, 128.9, 127.5, 127.1, 122.2, 121.1, 47.6, 35.4, 30.5. MS (ESI^+) *m/z* 390.93 $[M+H]^+$, 348.98 $[M–Ac+H]^+$.

S-(1-((1*H*-Benzo[d]imidazol-2-yl)amino)-1-oxo-3-phenylpropan-2-yl) ethanethioate (26b).

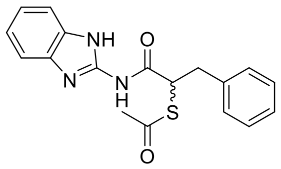

Compound **26b** was prepared according to general procedure **D**, using compound **26a** (172 mg, 0.53 mmol) and potassium thioacetate (112 mg, 0.79 mmol) in acetone (12 mL). Purification was done by column chromatography (Hex/EtOAc 6:1). The final product was obtained as yellow solid (136 mg, 85%). ^{1}H NMR (500 MHz, $CDCl_3$) δ ppm: 12.06 (s, 1H), 11.62 (s, 1H), 7.42 (br s, 2H), 7.33–7.14 (m, 5H), 7.07 (dd, J = 5.9, 3.0 Hz, 2H), 3.93 (d, J = 6.3 Hz, 1H), 3.34 (s, 3H), 3.27 (d, J = 9.0 Hz, 1H), 2.99 (dd, J = 13.7, 6.4 Hz, 1H). MS (ESI^+) *m/z* 340.08 $[M+H]^+$, 297.03 $[M–Ac+H]^+$.

4-(2-(Acetylthio)-3-phenylpropanamido)phenyl acetate (13c).

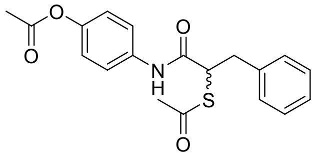

Compound **13c** was prepared according to general procedure **D**, using compound **13b** (280 mg, 0.88 mmol) and potassium thioacetate (151 mg, 1.32 mmol) in acetone (10 mL). Purification was done by flash chromatography (Hex/EtOAc, 100:0 to 0:100). The final product was obtained as yellow solid (244 mg, 77%). ^{1}H NMR (500 MHz, acetone-d_6) δ ppm: 9.32 (br s, 1H), 7.58 (d, J = 8.8 Hz, 2H), 7.30–7.24 (m, 3H), 7.22–7.18 (m, 2H), 7.03 (d, J = 8.9 Hz, 2H), 4.41 (dd, J = 9.3, 6.0 Hz, 1H), 3.35 (dd, J = 13.6, 9.3 Hz, 1H), 2.95 (dd, J = 13.6, 6.0 Hz, 1H), 2.34 (s, 3H), 2.22 (s, 3H). ^{13}C NMR (126 MHz, $CDCl_3$) δ ppm: 195.0, 169.8, 169.0, 147.9, 139.1, 137.2, 130.1, 129.3, 127.7, 122.9, 121.2, 50.4, 39.3. MS (ESI^+) *m/z* 358.13 $[M+H]^+$, 282.03 $[M–HSAc+H]^+$.

4-(2-(Acetylthio)-3-oxo-3-(phenylamino)propyl)phenyl acetate (15c).

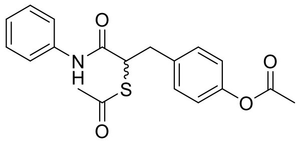

Compound **14c** was prepared according to general procedure **D**, using compound **14b** (130 mg, 0.41 mmol) and potassium thioacetate (70 mg, 0.61 mmol). Purification was done via flash chromatography (Hex/EtOAc, 100:0 to 0:100). The product was obtained as yellow oil (125 mg, 87%). ^{1}H NMR (126 MHz, $CDCl_3$) δ ppm: 7.97 (s, 1H), 7.46 (d, J = 7.9 Hz, 2H), 7.33–7.26 (m, 4H), 7.11 (br d, J = 7.3 Hz, 1H), 7.02 (d, J = 8.4 Hz, 2H), 4.29–4.25 (m, 1H), 3.45 (dd, J = 14.2, 8.5 Hz, 1H), 2.99 (dd, J = 14.2, 7.0 Hz, 1H), 2.39 (s, 3H), 2.29 (s, 3H). MS (ESI^+) *m/z* 358.10.08 $[M+H]^+$, 316.10 $[M–Ac+2H]^+$.

4-(2-(Acetylthio)-3-oxo-3-(phenylamino)propyl)-2-nitrophenyl acetate (16c).

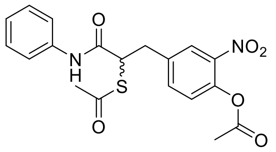

Compound **16c** was prepared according to general procedure **D**, using compound **16b** (185 mg, 0.51 mmol) and potassium thioacetate (87 mg, 0.76 mmol). Purification was done by flash chromatography (Hex/EtOAc, 100:0 to 0:100). The product was obtained as yellow oil (166 mg, 81%). ^{1}H NMR (500 MHz, acetone-d_6) δ ppm: 9.36 (br s, 1H),

8.09 (d, *J* = 1.7 Hz, 1H), 7.71 (dd, *J* = 8.2, 1.8 Hz, 1H), 7.56 (d, *J* = 8.2 Hz, 2H), 7.32 (d, *J* = 8.4 Hz, 1H), 7.28 (t, *J* = 7.9 Hz, 2H), 7.07 (t, *J* = 7.4 Hz, 1H), 4.51 (dd, *J* = 8.7, 6.6 Hz, 1H), 3.50 (dd, *J* = 13.8, 8.8 Hz, 1H), 3.12 (dd, *J* = 13.7, 6.4 Hz, 1H), 2.35 (s, 3H), 2.30 (s, 3H). ^{13}C NMR (126 MHz, acetone-d_6) δ ppm: 194.8, 169.1, 168.5, 143.6, 142.6, 139.6, 138.5, 137.0, 129.7, 127.1, 126.1, 124.9, 120.5, 49.8, 38.0, 20.7. MS (ESI$^+$) *m/z* 402.09 [M+H]$^+$.

2-Mercapto-*N*-(4-nitrophenyl)-3-phenylpropanamide (11).

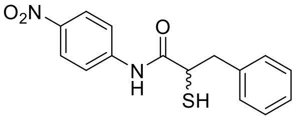

Compound **11** was prepared according to general procedure **E**, using compound **11b** (95 mg, 0.28 mmol) and 2 M NaOH aq. solution (280 µL, 0.56 mmol) in MeOH (2 mL). Purification was done by flash chromatography (Hex/EtOAc, 100:0 to 0:100). The final product was obtained as colorless oil (52 mg, 61%). ^{1}H NMR (500 MHz, $CDCl_3$) δ ppm: 8.35 (br s, 1H), 8.24–8.20 (m, 2H), 7.69–7.64 (m, 2H), 7.34–7.27 (m, 3H), 7.25–7.22 (m, 2H), 3.77 (dt, *J* = 9.1, 6.6 Hz, 1H), 3.37 (dd, *J* = 13.9, 6.4 Hz, 1H), 3.28 (dd, *J* = 13.9, 6.9 Hz, 1H), 2.15 (d, *J* = 9.0 Hz, 1H). ^{13}C NMR (126 MHz, $CDCl_3$) δ ppm: 170.2, 144.1, 143.1, 137.0, 129.6, 128.9, 127.5, 125.2, 119.4, 46.0, 41.4. HRMS (ESI$^-$) m/z calcd. for $C_{15}H_{13}N_2O_3S$ [M–H]$^-$ 301.06523, found 301.06518.

2-Mercapto-*N*-(4-methoxyphenyl)-3-phenylpropanamide (12).

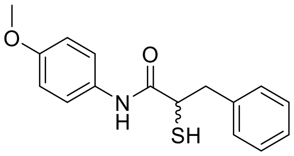

Compound **12** was prepared according to general procedure **E**, using compound **12b (**95 mg, 0.29mmol) and 2 M NaOH aq. solution (290 µL, 0.58 mmol) in MeOH (2 mL). Purification was done by flash chromatography (Hex/EtOAc, 100:0 to 0:100). The final product was obtained as white solid (45 mg, 54%). ^{1}H NMR (500 MHz, $CDCl_3$) δ ppm: 7.90 (br s, 1H), 7.37–7.33 (m, 2H), 7.31 (d, *J* = 7.5 Hz, 2H), 7.28–7.23 (m, 3H), 6.89–6.84 (m, 2H), 3.80 (s, 3H), 3.70 (dt, *J* = 8.9, 6.6 Hz, 1H), 3.36 (dd, *J* = 13.7, 6.7 Hz, 1H), 3.24 (dd, *J* = 14.0, 6.4 Hz, 1H), 2.10 (d, *J* = 8.9 Hz, 1H). ^{13}C NMR (126 MHz, $CDCl_3$) δ ppm: 169.5, 156.9, 137.5, 130.4, 129.6, 128.7, 127.3, 122.1, 114.3, 55.6, 45.9, 41.7. HRMS (ESI$^+$) m/z calcd. for $C_{16}H_{18}NO_2S$ [M+H]$^+$ 288.10527, found 288.10453.

***N*-(4-Hydroxyphenyl)-2-mercapto-3-phenylpropanamide (13).**

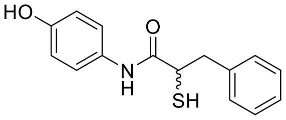

Compound **13** was prepared according to general procedure **E**, using compound **13c** (240 mg, 0.67 mmol) and 2M NaOH aq. solution (1.05 mL, 2.1 mmol) in MeOH (2 mL). Purification was done by flash chromatography (Hex/EtOAc, 100:0 to 0:100). The final product was obtained as white solid (68 mg, 37%). ^{1}H NMR (500 MHz, acetone-d_6) δ ppm: 9.05 (br s, 1H), 8.22 (s, 1H), 7.38 (d, *J* = 8.8 Hz, 2H), 7.32–7.17 (m, 5H), 6.75 (d, *J* = 9.3 Hz, 2H), 3.70 (td, *J* = 8.9, 6.5 Hz, 1H), 3.32 (dd, *J* =13.5, 8.8 Hz, 1H), 2.99 (dd, *J* = 13.6, 6.3 Hz, 1H), 2.50 (d, *J* = 9.5 Hz, 1H). ^{13}C NMR (126 MHz, acetone-d_6) δ ppm:170.6, 154.7, 139.8, 132.0, 130.1, 129.1, 127.4, 122.1, 116.0, 45.2, 43.3. HRMS (ESI$^-$) m/z calcd. for $C_{15}H_{14}NO_2S$ [M–H]$^-$ 272.07507, found 272.07520.

2-Mercapto-*N*-phenyl-3-(*p*-tolyl)propenamide (14).

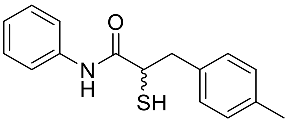

Compound **14** was prepared according to general procedure **E**, using compound **14b** (90 mg, 0.29 mmol) and 2 M NaOH aq. solution (290 µL, 0.58 mmol) in MeOH (5 mL). Purification was done by flash chromatography (Hex/EtOAc, 100:0 to 0:100). The product was obtained as white solid (55 mg, 70%). ^{1}H NMR (500 MHz, $CDCl_3$) δ ppm: 8.02 (br s, 1H), 7.48 (br d, *J* = 7.8 Hz, 2H), 7.34 (t, *J* = 7.9 Hz, 2H), 7.17–7.09 (m, 5H), 3.70 (dt, *J* = 8.6, 6.7 Hz,

1H), 3.33 (dd, J = 13.9, 6.7 Hz, 1H), 3.21 (dd, J = 13.8, 6.8 Hz, 1H), 2.33 (s, 3H), 2.09 (d, J = 8.9 Hz, 1H). ^{13}C NMR (126 MHz, $CDCl_3$) δ ppm: 169.6, 137.3, 136.8, 134.1, 129.3, 129.3, 129.0, 124.7, 120.0, 46.0, 41.0, 21.1. HRMS (ESI^+) m/z calcd. for $C_{16}H_{18}NOS$ $[M+H]^+$ 272.11036, found 272.10971.

3-(4-Hydroxyphenyl)-2-mercapto-*N*-phenylpropanamide (15).

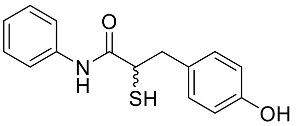

Compound **15** was prepared according to general procedure **E**, using compound **15c** (120 mg, 0.34 mmol) and 2 M NaOH aq. solution (340 µL, 0.68 mmol) in MeOH (2 mL). Purification was done by flash chromatography (Hex/EtOAc, 100:0 to 0:100). The final product was obtained as white solid (41 mg, 44%). ^{1}H NMR (500 MHz, Acetone-d_6) δ ppm: 9.21 (br s, 1H), 8.16 (br s, 1H), 7.59 (d, J = 8.2 Hz, 2H), 7.09 (d, J = 8.4 Hz, 2H), 7.27 (t, J = 7.9 Hz, 2H), 7.05 (t, J = 7.3 Hz, 1H), 6.72 (d, J = 8.4 Hz, 2H), 3.67 (dd, J = 8.7, 6.1 Hz, 1H), 3.24 (dd, J = 13.7, 8.9 Hz, 1H), 2.91 (dd, J = 13.7, 6.1 Hz, 1H), 2.48 (br s, 1H). ^{13}C NMR (126 MHz, Acetone-d_6) δ ppm: 171.5, 157.1, 140.1, 131.1, 130.4, 129.6, 124.5, 120.3 116.0, 45.6, 42.5. HRMS (ESI^+) m/z calcd. for $C_{15}H_{16}NO_2S$ $[M+H]^+$ 274.08962, found 274.08893.

3-(4-Hydroxy-3-nitrophenyl)-2-mercapto-*N*-phenylpropanamide (16).

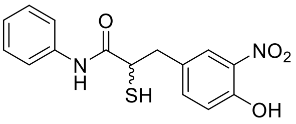

Compound **16** was prepared according to general procedure **E**, using compound **16c** (160mg, 0.40 mmol) and 2 M NaOH aq. solution (600 µL, 1.2 mmol) in MeOH (10 mL). Purification was done by flash chromatography (Hex/EtOAc, 100:0 to 0:100). The final product was obtained as light green solid (70 mg, 50%). ^{1}H NMR (500 MHz, acetone-d_6) δ ppm: 10.33 (s, 1H), 9.33 (br s, 1H), 8.06 (d, J = 2.0 Hz, 1H), 7.63 (dd, J = 8.5, 2.1 Hz, 1H), 7.58 (d, J = 8.1 Hz, 2H), 7.28 (t, J=7.9 Hz, 2H), 7.12 (d, J = 8.5 Hz, 1H), 7.06 (t, J = 7.3 Hz, 1H), 3.79 (dd, J = 8.9, 7.0 Hz, 1H), 3.36 (dd, J = 13.7, 8.4 Hz, 1H), 3.07 (dd, J = 13.8, 6.6 Hz, 1H), 2.60 (d, J = 9.8 Hz, 1H). ^{13}C NMR (126 MHz, acetone-d_6) δ ppm: 170.9, 154.2, 139.8, 139.6, 134.7, 132.1, 129.6, 126.1, 124.6, 120.5, 120.3, 44.9, 41.4. HRMS (ESI^-) m/z calcd. for $C_{15}H_{13}N_2O_4S$ $[M–H]^-$ 317.06015, found 317.06003.

3-(4-Chlorophenyl)-2-mercapto-*N*-phenylpropanamide (17).

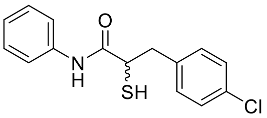

Compound **17** was prepared according to general procedure **E**, using compound **17b** (28 mg, 0.08 mmol) and 2 M NaOH aq. solution (17 µL, 0.17 mmol) in MeOH (2 mL). Purification was done by preparative HPLC. The final product was obtained as white solid (7 mg, 29%). ^{1}H NMR (500 MHz, acetone-d_6) δ ppm: 9.26 (s, 1H), 7.57 (d, J = 8.2 Hz, 2H), 7.32–7.24 (m, 6H), 7.05 (t, J = 7.4 Hz, 1H), 3.77–3.70 (m, 1H), 3.33 (dd, J = 13.6, 8.6 Hz, 1H), 3.00 (dd, J = 13.7, 6.4 Hz, 1H), 2.56 (d, J = 9.6 Hz, 1H). ^{13}C NMR (125 MHz, acetone-d_6) δ ppm: 171.0, 139.9, 138.6, 132.8, 132.0, 129.5, 129.1, 124.6, 120.2, 45.0, 42.2. HRMS (ESI^+) m/z calcd. for $C_{15}H_{15}ClNOS^+$ $[M+H]^+$ 292.0557, found 292.0554.

2-Mercapto-3-phenyl-*N*-(thiazol-2-yl)propenamide (18).

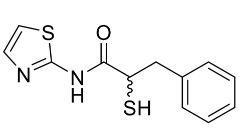

Compound **18** was prepared according to general procedure **E**, using compound **18b** (219 mg, 0.71 mmol) and 2 M NaOH aq. solution (714 µL, 1.43 mmol) in MeOH (5 mL). Purification was done by column chromatography (Hex/EtOAc, 7:3). The final product was obtained as white solid (25 mg, 15%). ^{1}H NMR (500 MHz, DMSO-d_6) δ ppm:12.18 (s, 1H), 7.46 (d, J = 3.5 Hz, 1H), 7.30–7.24 (m, 2H), 7.24–7.16 (m, 4H), 3.90 (t, J = 7.6 Hz, 1H), 3.26 (dd, J = 13.7, 8.8 Hz, 1H), 2.97 (dd, J = 13.7, 6.6 Hz, 1H), 2.53–2.51 (m, 1H). ^{13}C NMR (126 MHz,

DMSO-d_6) δ ppm: 170.6, 157.7, 138.3, 137.8, 129.0, 128.3, 126.6, 113.8, 41.7, 40.6. HRMS (ESI^{-}) m/z calcd. for $C_{12}H_{11}N_2OS_2$ $[M–H]^{-}$ 263.03182, found 263.03189.

Methyl 2-(2-mercapto-3-phenylpropanamido)thiophene-3-carboxylate (19).

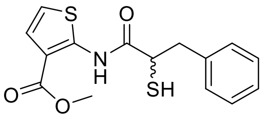

Compound **19** was prepared according to general procedure **E**, using compound **19b** (129 mg, 0.52 mmol) and solid NaOH (40 mg, 1.03 mmol) in MeOH (3 mL). Purification was done by preparative HPLC. The final product was obtained as white solid (94 mg, 82%). ^{1}H NMR (500 MHz, DMSO-d_6) δ ppm: 10.45 (s, 1H), 7.91 (dd, *J* = 13.1, 5.4 Hz, 2H), 7.36–7.09 (m, 5H), 4.06 (t, *J* = 7.4 Hz, 1H), 3.82 (s, 3H), 3.27 (dd, *J* = 13.9, 7.4 Hz, 1H), 2.97 (dd, *J* = 13.9, 7.4 Hz, 1H), 2.52–2.51 (m, 1H). ^{13}C NMR (126 MHz, DMSO-d_6) δ ppm: 170.0, 163.2, 143.2, 138.2, 133.1, 129.2, 128.3, 126.6, 122.2, 111.0, 52.2, 43.6, 40.7. HRMS (ESI^{+}) m/z calcd. for $C_{15}H_{16}NO_3S_2$ $[M+H]^{+}$ 322.05661, found 322.05664.

2-Mercapto-3-phenyl-*N*-(pyridin-3-yl)propenamide (20).

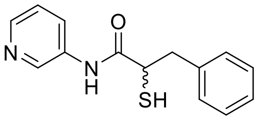

Compound **20** was prepared according to general procedure **E**, using compound **20b** (108 mg, 0.35 mmol) and solid NaOH (28 mg, 0.71 mmol) in MeOH (2 mL). Purification was done by preparative HPLC. The final product was obtained as white solid (24 mg, 26%). ^{1}H NMR (500 MHz, DMSO-d_6) δ ppm: 10.25 (s, 1H), 8.65 (d, *J* = 2.4 Hz, 1H), 8.25 (dd, *J* = 4.7, 1.5 Hz, 1H), 7.97 (dd, *J* = 8.3, 1.0 Hz, 1H), 7.33 (dd, *J* = 8.3, 4.7 Hz, 1H), 7.30–7.17 (m, 5H), 3.79–3.72 (m, 1H), 3.25 (dd, *J* = 13.7, 8.7 Hz, 1H), 3.17 (d, *J* = 5.2 Hz, 1H), 2.98–2.93 (m, 1H). ^{13}C NMR (126 MHz, DMSO-d_6) δ ppm: 171.1, 144.5, 140.8, 138.5, 135.5, 129.0, 128.3, 126.6, 126.2, 123.7, 43.1, 41.1. HRMS (ESI^{-}) m/z calcd. for $C_{14}H_{13}N_2OS$ $[M–H]^{-}$ 257.07540, found 257.07547.

2-Mercapto-3-phenyl-*N*-(pyridin-2-yl)propenamide (21).

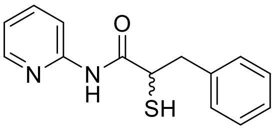

Compound **21** was prepared according to general procedure **E**, using compound **21b** (20 mg, 0.07 mmol), acetyl chloride (94 µL, 1.33 mmol) in MeOH (3 mL). Purification was done by preparative HPLC. The final product was obtained as white solid (5 mg, 29%). ^{1}H NMR (500 MHz, acetone-d_6) δ ppm: 9.53 (s, 1H), 8.23 (dd, *J* = 4.8, 0.9 Hz, 1H), 8.18 (d, *J* = 8.3 Hz, 1H), 7.75 (td, *J* = 8.8, 1.8 Hz, 1H), 7.32–7.24 (m, 4H), 7.21–7.16 (m, 1H), 7.09–7.04 (m, 1H), 4.06–3.97 (m, 1H), 3.38 (dd, *J* = 13.7, 8.5 Hz, 1H), 3.04 (dd, *J* = 13.7, 6.4 Hz, 1H), 2.58 (d, *J* = 9.7 Hz, 1H). ^{13}C NMR (126 MHz, acetone-d_6) δ ppm: 172.0, 152.9, 148.9, 139.6, 138.8, 130.1, 129.1, 127.4, 120.4, 114.3, 44.7, 42.7. HRMS (ESI^{+}) m/z calcd. for $C_{14}H_{15}N_2OS^{+}$ $[M+H]^{+}$ 259.0900, found 259.0905.

2-Mercapto-3-phenyl-*N*-(pyridin-4-yl)propanamide (22).

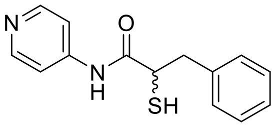

Compound **22** was prepared according to general procedure **E**, using compound **22b** (13 mg, 0.04 mmol) and acetyl chloride (49 µL, 0.69 mmol) in MeOH (2 mL). Once the conversion was complete, the solvent was removed under reduced pressure to obtain the crude product. Purification was done by preparative HPLC. The final product was obtained as white solid (4 mg, 35%). ^{1}H NMR (500 MHz, acetone-d_6) δ ppm: 9.76 (s, 1H), 8.42 (m, 2H), 7.58 (d, *J* = 4.6 Hz, 2H), 7.32–7.16 (m, 5H), 3.85–3.76 (m, 1H), 3.35 (dd, *J* = 13.7, 8.7 Hz, 1H), 3.02 (dd, *J* = 13.7, 6.4 Hz, 1H), 2.66 (d, *J* = 7.4 Hz, 1H). ^{13}C NMR (126 MHz, acetone-d_6) δ ppm: 172.5, 151.1, 146.8, 139.5, 130.1, 129.2, 127.5, 114.2, 45.0, 42.5. HRMS (ESI^{+}) m/z calcd. for $C_{14}H_{15}N_2OS^{+}$ $[M+H]^{+}$ 259.0900, found 259.0896.

***N*-(Benzo[d]thiazol-2-yl)-2-mercapto-3-phenylpropanamide (23).**

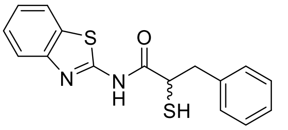

Compound **23** was prepared according to general procedure **E**, using compound **23b** (128 mg, 0.36 mmol) and 2 M NaOH aq. solution (359 µL, 0.72 mmol) in MeOH (3 mL). Purification was done by flash chromatography (Hex/EtOAc, 7:3). The final product was obtained as white solid (30 mg, 28%).^{1}H NMR (500 MHz, $CDCl_3$) δ ppm: 7.85 (d, *J* = 7.8 Hz 1H), 7.76 (d, *J* = 8.1, 1H), 7.49–7.44 (m, 1H), 7.39–7.36 (m, 1H), 7.29–7.27 (m, 1H), 7.25–7.15 (m, 4H), 3.87–3.80 (m, 1H), 3.40 (dd, *J* = 14.0, 7.0 Hz, 1H), 3.24 (dd, *J* = 14.0, 6.8 Hz, 1H), 2.26–2.17 (m, 1H).^{13}C NMR (126 MHz, $CDCl_3$) δ ppm: 170.7, 159.0, 145.5, 136.7, 131.0, 129.4, 128.9, 127.5, 127.2, 125.0, 121.9, 120.2, 44.7, 41.1. HRMS (ESI$^+$) m/z calcd. for $C_{16}H_{15}N_2OS_2$ $[M+H]^+$ 315.06203, found 315.06178.

2-Mercapto-*N*-(6-methoxybenzo[d]thiazol-2-yl)-3-phenylpropanamide (24).

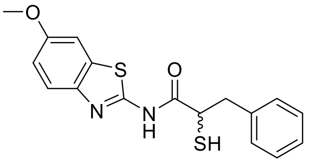

Compound **24** was prepared according to general procedure **E**, using compound **24b** (225 mg, 0.58 mmol) and 2 M NaOH aq. solution (582 µL, 1.64 mmol) in MeOH (3 mL). Purification was done by column chromatography (Hex/EtOAc, 3:1). The final product was obtained as white solid (104 mg, 52%). ^{1}H NMR (500 MHz, DMSO-*d$_6$)* δ ppm: 12.33 (s, 1H), 7.62 (d, *J* = 8.8 Hz, 1H), 7.57 (d, *J* = 2.6 Hz, 1H), 7.31–7.13 (m, 5H), 7.02 (dd, *J* = 8.8, 2.6 Hz, 1H), 3.91 (dd, *J* = 8.5, 6.8 Hz, 1H), 3.80 (s, 3H), 3.37 (br s, 1H), 3.28 (dd, *J* = 13.8, 8.5 Hz, 1H), 2.98 (dd, *J* = 13.8, 6.7 Hz, 1H). ^{13}C NMR (126 MHz, DMSO-*d$_6$)* δ ppm: 171.4, 156.2 155.7, 142.6 138.3, 132.8, 129.1, 128.3, 126.7, 121.3, 115.0, 104.7, 55.7, 41.9, 40.5. HRMS (ESI$^-$) m/z calcd. for $C_{17}H_{15}N_2O_2S_2$ $[M–H]^-$ 343.05804, found 343.05835.

***N*-(6-Chlorobenzo[d]thiazol-2-yl)-2-mercapto-3-phenylpropanamide (25).**

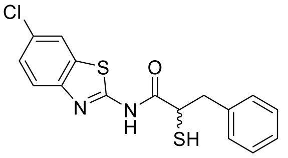

Compound **25** was prepared according to general procedure **E**, using compound **25b** (105 mg, 0.27 mmol) and 2 M NaOH aq. solution (270 µL, 0.54 mmol) in MeOH (2 mL). Purification was done by preparative HPLC. The final product was obtained as white solid (78 mg, 84%). ^{1}H NMR (500 MHz, DMSO-*d$_6$)* δ ppm: 12.54 (br s, 1H), 8.13 (d, *J* = 2.2 Hz, 1H), 7.72 (d, *J* = 8.6 Hz, 1H), 7.45 (dd, *J* = 8.6, 2.2 Hz, 1H), 7.34–7.13 (m, 5H), 3.95–3.91 (m, 1H), 3.30–3.25 (m, 1H), 3.00 (dd, *J* = 13.8, 6.9 Hz, 1H), 2.52–2.51 (m, 1H). ^{13}C NMR (126 MHz, DMSO-*d$_6$)* δ ppm: 171.9, 158.6, 147.4, 138.2, 133.2, 129.03, 128.3, 127.7, 126.7, 126.6, 121.8, 121.5, 41.9, 40.3. HRMS (ESI$^-$) m/z calcd. for $C_{16}H_{12}ClN_2OS_2$ $[M–H]^-$ 349.02305, found 349.02304.

***N*-(1*H*-Benzo[d]imidazol-2-yl)-2-mercapto-3-phenylpropanamide (26).**

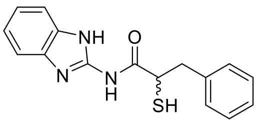

Compound **26** was prepared according to general procedure **E**, using compound **26b** (120 mg, 0.35 mmol) and solid NaOH (27 mg, 0.70 mmol) in MeOH (2 mL). Purification was done by preparative HPLC. The final product was obtained as white solid (85 mg, 80%). ^{1}H NMR (500 MHz, DMSO-*d$_6$)* δ ppm: 12.06 (s, 1H), 11.64 (s, 1H), 7.42 (s, 2H), 7.33–7.14 (m, 5H), 7.13–7.04 (m, 2H), 3.93 (d, *J* = 5.9 Hz, 1H), 3.31–3.26 (m, 1H), 3.26 (br s, 1H), 2.99 (dd, *J* = 13.7, 6.4 Hz, 1H).^{13}C NMR (126 MHz, DMSO-*d$_6$*). HRMS (ESI$^-$) m/z calcd. for $C_{16}H_{16}N_3OS$ $[M–H]^-$ 298.10085, found 298.10069.

Tables and Figures

Table S1. K_i values for six selected compounds against ColH-PD and % inhibition of ColH-PD at 1 μM concentration of six selected compounds. K_i values and residual activities are determined as described previously.[3]

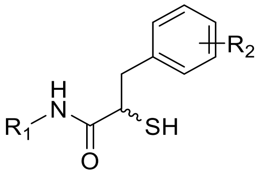

Compound	R_1	R_2	K_i (μM)
**3**	Ph	4-Me	0.05 ± 0.01
**5**	Ph	H	0.4 ± 0.04
**12**	4-OMe-Ph	H	0.04 ± 0.01
**13**	4-OH-Ph	H	0.1 ± 0.02
**23**	benzothiazolyl	H	0.1 ± 0.01
**24**	6-methoxybenzothiazolyl	H	28 ± 1
**Compound**	**R_1**	**R_2**	**% inh. of ColH-PD @1µM**
**11**	4-NO_2-Ph	H	88 ± 2
**14**	Ph	4-Me-Ph	69 ± 3
**15**	Ph	4-OH	63 ± 2
**16**	Ph	3-NO_2-4-OH	74 ± 5
**18**	thiazolyl	H	34 ± 3
**24**	6-chlorobenzothiazolyl	H	73 ± 2

Table S2. Zebrafish embryotoxicity results for compounds **12** and **23**.

Compound	Concentration (*µ*M)	2 dpf	3 dpf	4 dpf	5 dpf	Survival rate %
**12**	100	all dead	-	-	-	0
	50	imp. dev., turbid body	all dead	-	-	0
	30	imp. dev.	all dead	-	-	0
	2	OK	OK	OK	OK	100
**23**	100	imp. dev.	5 imp. dev.	5 imp. dev.	5 imp. dev.	0
	50	imp. dev.	5 imp. dev.	5 imp. dev.	5 imp. dev.	50
	30	OK	OK, 3 imp. dev.	OK, 3 imp. dev.	OK, 3 imp. dev.	70
	2	OK	OK	OK	OK	100
Danieau's ctrl	-	OK	OK, 1 malf., 1 dead	OK	OK	80
DMSO ctrl	1%	OK	OK, 1 malf.	OK	OK	100

malf. = body curvature

impaired dev. = impaired development, pericardial edema

No toxicity signs were observed for compound **12** at a concentration of 2 µM. However, all concentrations above (30 µM, 50 µM, and 100 µM) have led to a toxicity of 100%.

30% of larvae showed toxicity signs, such as impaired body development and pericardial edema, when incubated with compound **23** at a concentration of 30 µM. The two highest concentrations (50 µM and 100 µM) were lethal for all larvae resulting in a survival rate of 0%.

A comparable ratio of malformation was also found in the control groups (with only Danieau’s medium or 1% DMSO). Therefore, observed body malformation in larvae incubated in compound can be considered as not related to compound treatment.

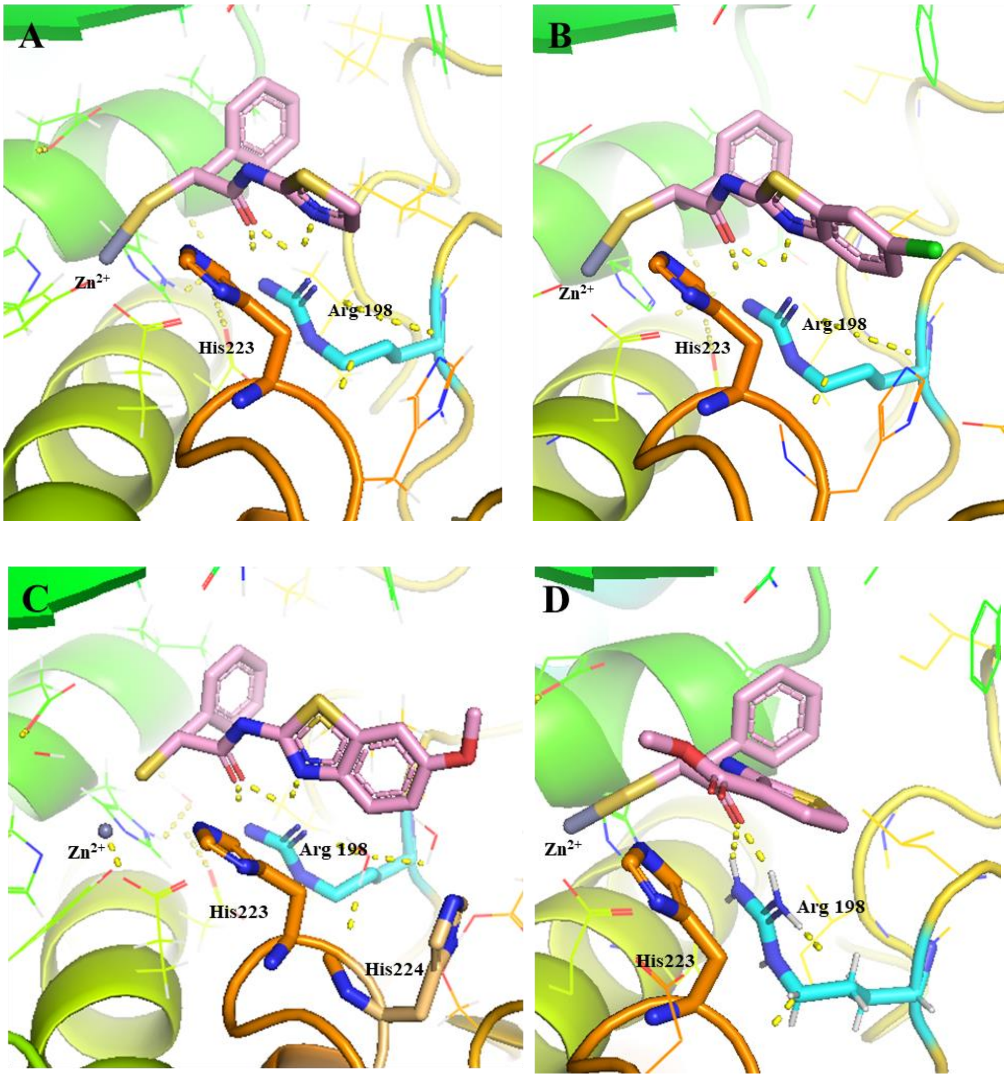

Figure S1. Docking poses for **A)** Thiazole, **B)** 6-chlorobenzothiazolyl, **C)** 6-methoxybenzothiazolyl and **D)** Methyl thiophenyl 3-carboxylate replacement in the LasB ligand binding pocket. The interactions in the binding pocket of LasB are predicted by SeeSAR V.11.1 and visualized using PyMOL V.2.5 softwares..[10] The dashed lines represent H-bonds of less than 2.15 Å .

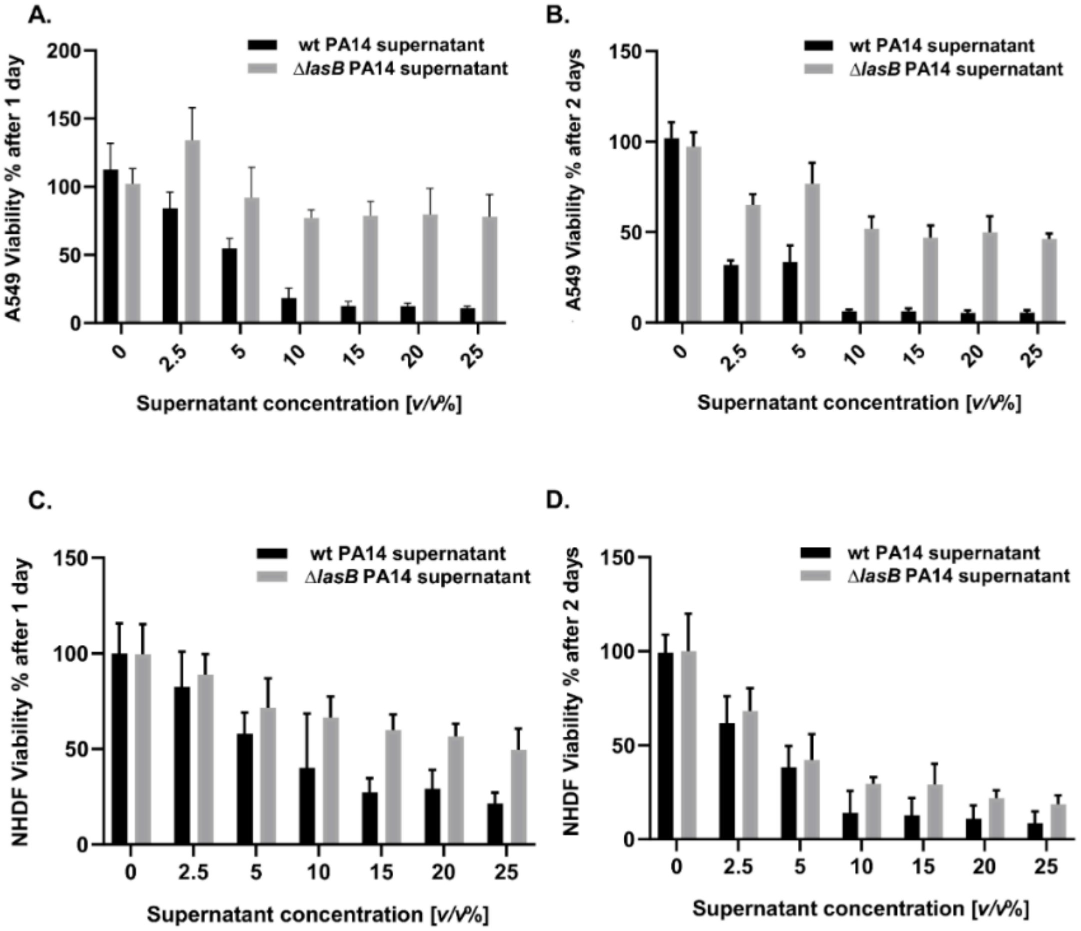

Figure S2. Illustration of the dose-dependent cytotoxic effect of wt PA14 and Δ*lasB* PA14 supernatant on normal human dermal fibroblast (NHDF) and adenocarcinomic human alveolar basal epithelial (A549) cells. **A)** wt PA14 supernatant reduces the viability of A549 cells after 24 h incubation compared with Δ*lasB* PA14 supernatant. **B)** wt PA14 supernatant effect on the cell viability after 48 h incubation with A549 cells, the viability is further minimized. **C)** wt PA14 supernatant effect on NHDF cell after 24 h incubation, its cytotoxic effect on NHDF cells is less that on A549 cells **D)** The cytotoxic effect of wt supernatant after 48 h incubation with NHDF cells is improved. This confirms that LasB is one of the major virulence factors present in the supernatant. The low cytotoxic effect observed with the Δ*lasB* PA14 supernatant might be due to effect of other extracellular toxins than LasB such as phospholipase, LasA, phytotoxic factors and exotoxins.[19] Each graph is a representation of three independent experiments, mean ± SD. The percentage shows the amount of supernatant in the whole volume of Dulbecco's Modified Eagle Medium (DMEM) and cells. PA14: wild-type *Pseudomonas aeruginosa*, ΔPA14: LasB knockout *P. aeruginosa*.

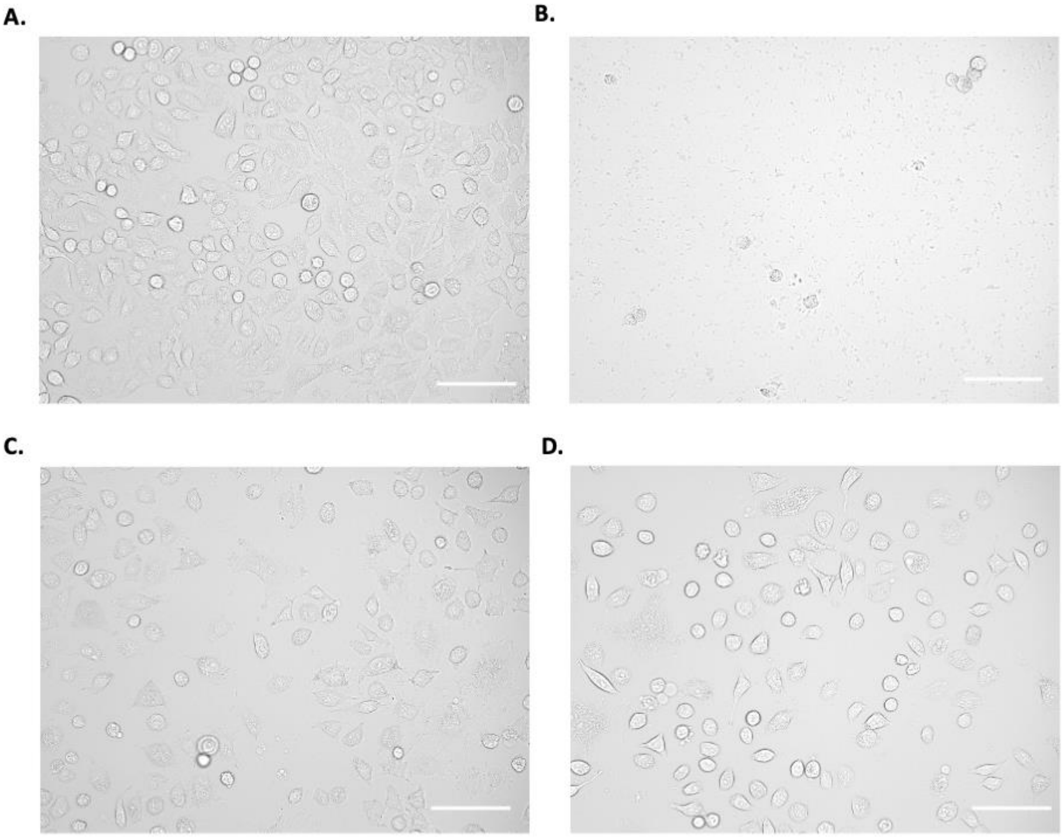

Figure S3. Visualization of differently treated adenocarcinomic human alveolar basal epithelial (A549) cells. **A)** Untreated cells; **B)** Cells treated with 15% (*v/v*) wt PA14 supernatant, cell density significantly reduced compared with untreated cells; **C)** A549 cells treated with 15% (*v/v*) Δ*lasB* PA14 supernatant; cell density is still high, and the morphology of the cells did not change; **D)** Cells challenged with wt PA14 supernatant and treated with Pam; their cell integrity and morphology were maintained. Images were generated with 20X objective by Leica Las X and modified with the software Fiji ImageJ (Scale bar: 100 μm). wt PA14: wild-type *P. aeruginosa*, Δ*lasB* PA14: LasB knockout *P. aeruginosa*. Pam: phosphoramidon.

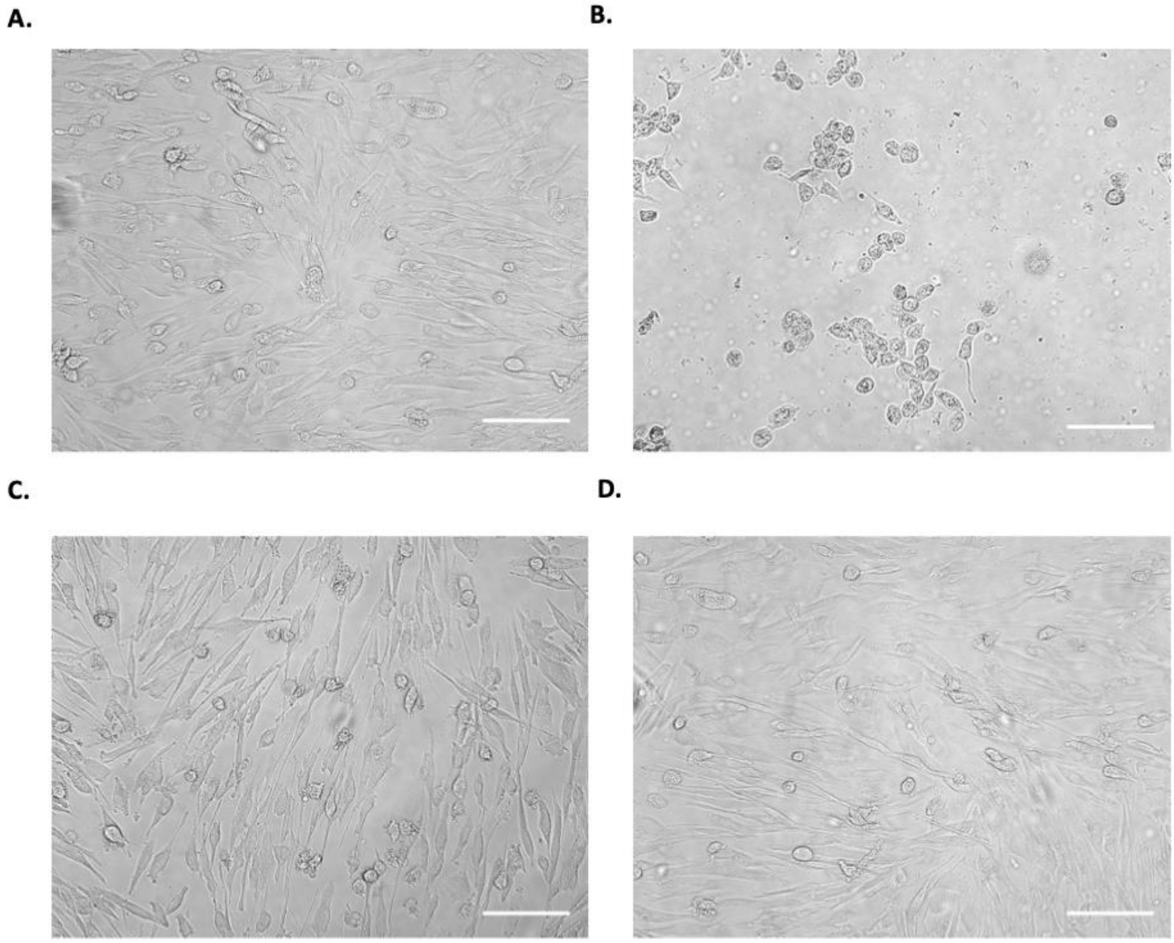

Figure S4. Visualization of differently treated normal human dermal fibroblast (NHDF) cells. **A)** Untreated cells; **B)** Cells treated with 15% (*v/v*) wt PA14 supernatant, cell density significantly reduced compared with untreated cells; **C)** Cells treated with 15% (*v/v*) Δ*lasB* PA14 supernatant; the cell density is still high, and the morphology of the cells did not change; **D)** Cells challenged with wt PA14 supernatant and treated with Pam; their cell integrity and morphology were maintained. Images were generated with 20X objective by Leica Las X and modified with the software Fiji ImageJ (Scale bar: 100 μm). wt PA14: wild-type *P. aeruginosa*, Δ*lasB* PA14: LasB knockout *P. aeruginosa*, Pam: phosphoramidon.

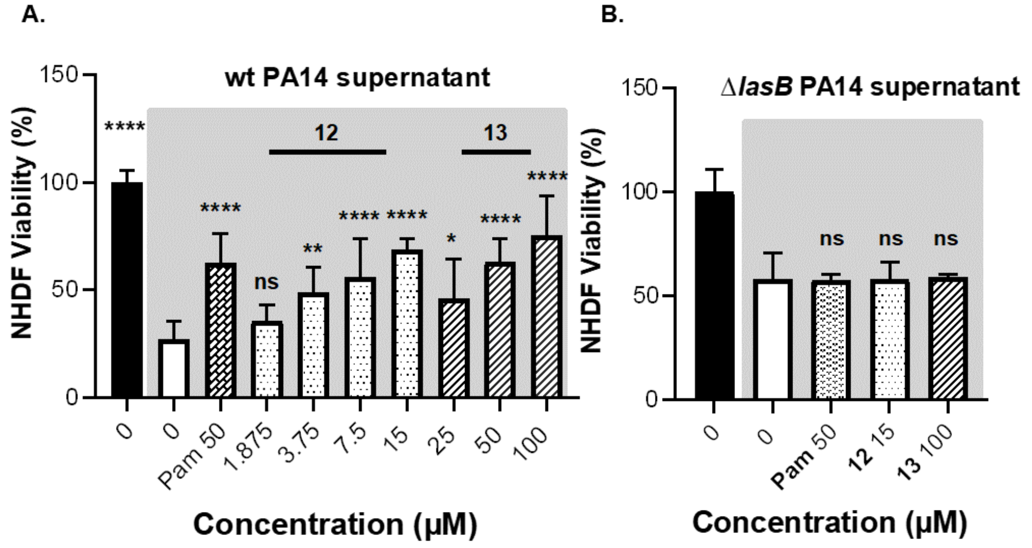

Figure S5. Viability of normal human dermal fibroblast (NHDF) cells treated with **12** and **13** and 15% (*v/v*) wt PA14 or Δ*lasB* PA14 supernatant. **A)** Concentrations-dependent effects of compounds on the viability of NHDF cells treated with wt PA14 supernatant;(c) **B)** Viability of NHDF cells treated with Δ*lasB* PA14 supernatantand the highest tested concentration of compound that was used with PA14 supernatant. Each graph is a representation of three independent experiments ± SD. One-way ANOVA was performed for each experiment following Dunnett's multiple comparisons test and moreover, the mean of each column was compared with the mean of the negative control (ns: not significant, *: $p \leq 0.05$, **: $p \leq 0.01$, ****: $p \leq 0.0001$). wt PA14: wild-type *Pseudomonas aeruginosa*, Δ*lasB* PA14: LasB knockout *P. aeruginosa*, Pam: phosphoramidon.

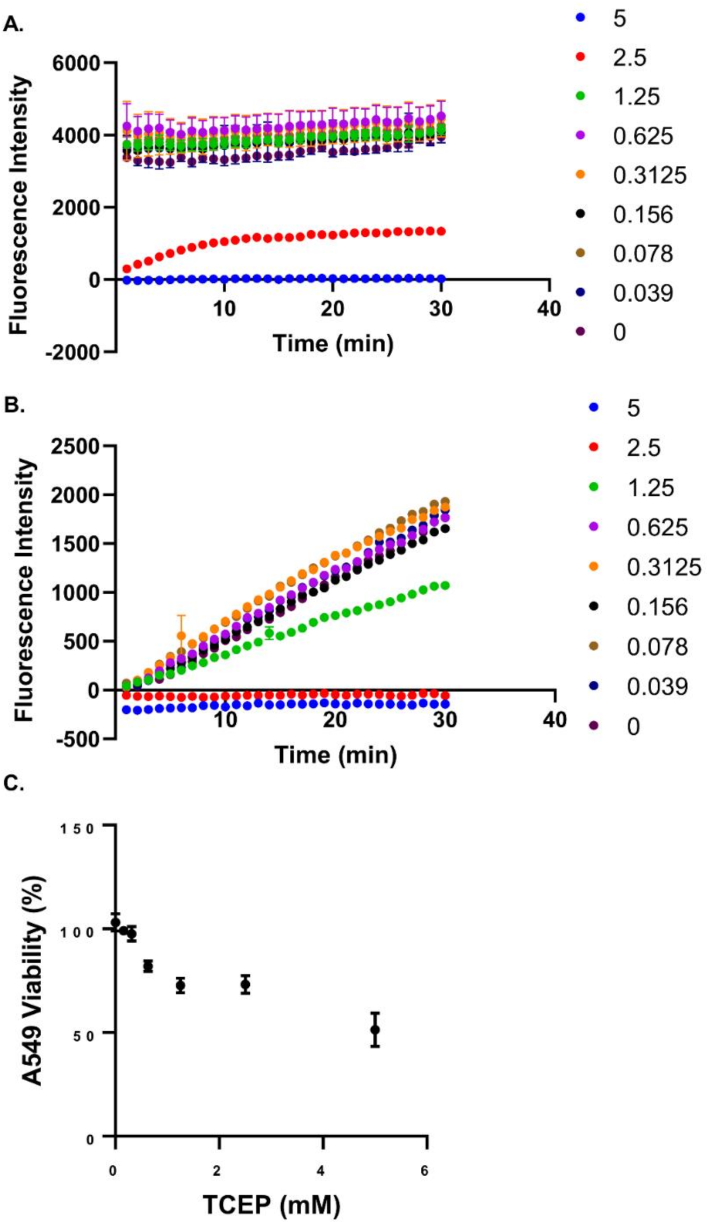

Figure S6. Effect of a reducing agent on LasB activity. **A)** Activity of LasB presented in 10% (*v/v*) wt PA14 supernatant incubated with different concentrations (mM) of TCEP. Similar to pure LasB high concentration of TCEP (*i.e.*, 5 and 2.5 mM) inhibited the activity of LasB in the supernatant. **B)** Effect of various concentrations (mM) of TCEP on 0.3 nM pure LasB. The activity was completely lost with 5 mM and 2.5 mM while at 0.6 mM and lower concentrations no inhibition was detected, similar to no TCEP conditions. **C)** Effect of TCEP concentrations on viability of A549 cells. 0.3 mM TCEP showed no effect on cell viability while higher concertations showed a reduction in the cell viability, which was evaluated with MTT assay. Each curve represents a mean ± SD of two independent experiments. wt PA14: wild-type *Pseudomonas aeruginosa*, TCEP: Tris(2-carboxyethyl)phosphine hydrochloride.

.

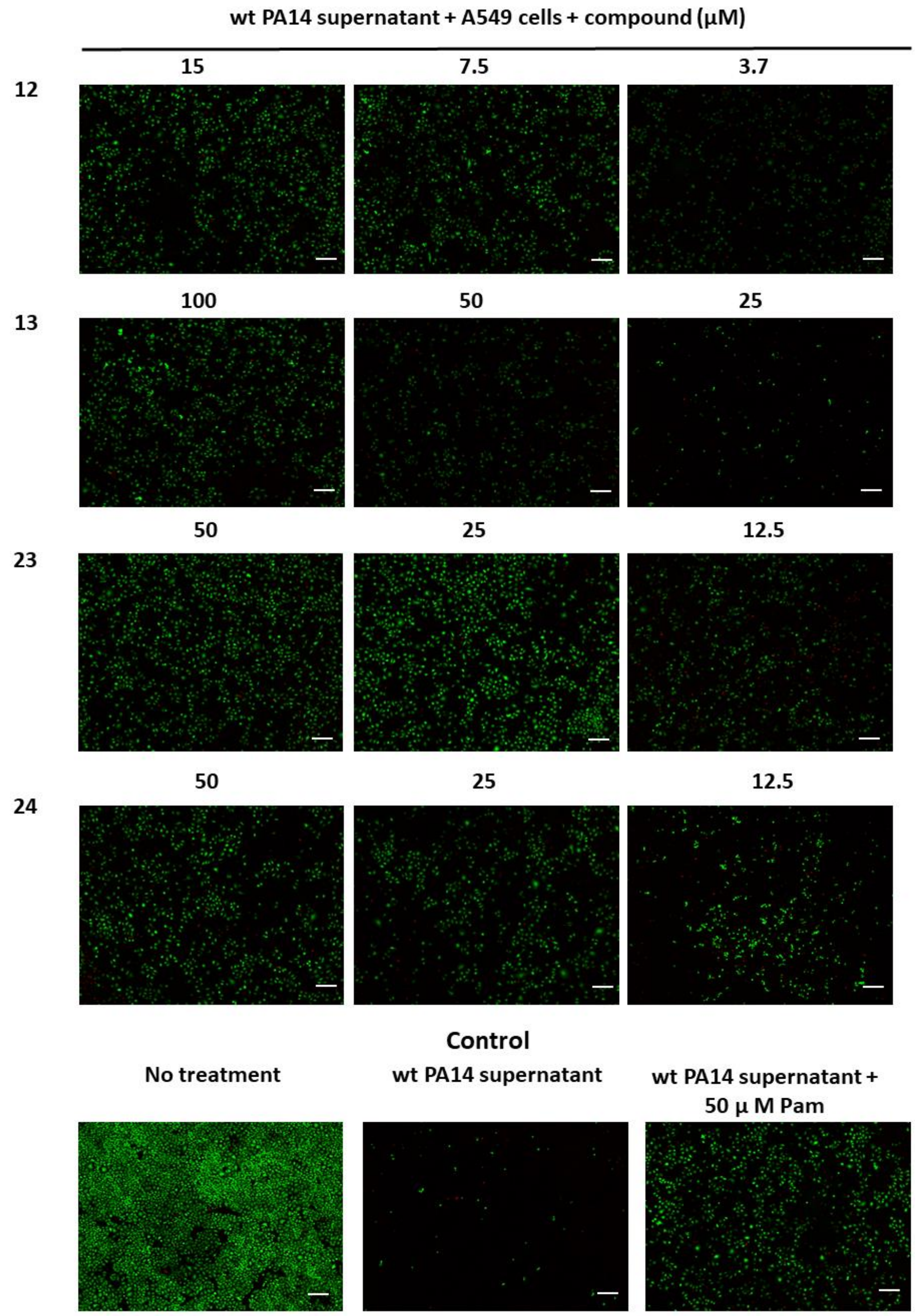

Figure S7. Visualization of the effects of compounds **12**, **13**, **23** and **24** on wt PA14 supernatant treated adenocarcinomic human alveolar basal epithelial (A549) cells. Live/dead staining was carried out with fluoresceine diacetate and propidium iodine. Living cells are shown in green and dead cells in red. Red signal in some cases was lost because the detached cells were washed away after the rinsing step with PBS (scale bar: 200 μm). wt PA14: wild-type *Pseudomonas aeruginosa*, Pam: phosphoramidon.

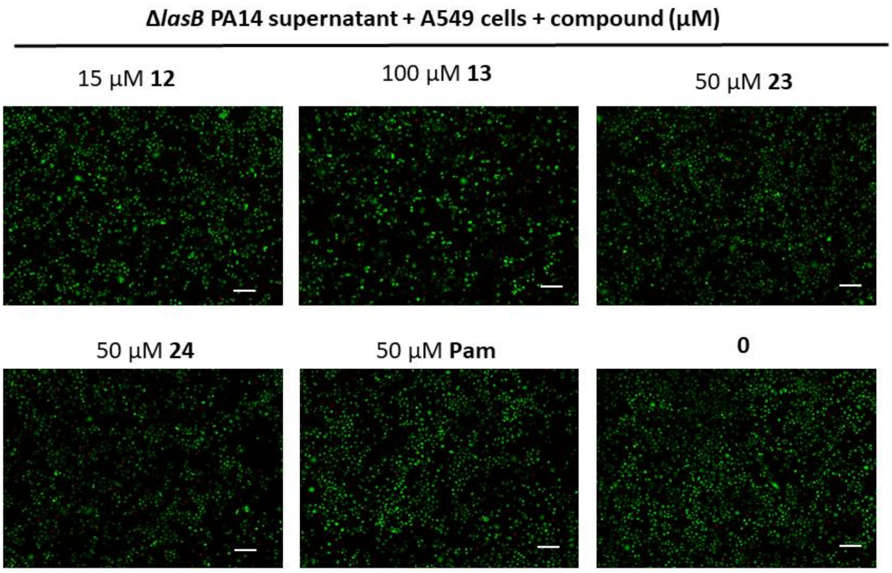

Figure S8. Visualization of effect of compounds **12**, **13**, **23** and **24** on Δ*lasB* PA14 supernatant applied adenocarcinomic human alveolar basal epithelial (A549) cells. Live/dead staining was carried out with fluoresceine diacetate and propidium iodine. Live cells are showed in green and dead cells in red. Scale bar: 200 µm. Red signal in some cases was lost because the detached cells were washed away after the rinsing step with PBS. Δ*lasB* PA14: LasB knockout *Pseudomonas aeruginosa*, Pam: phosphoramidon.

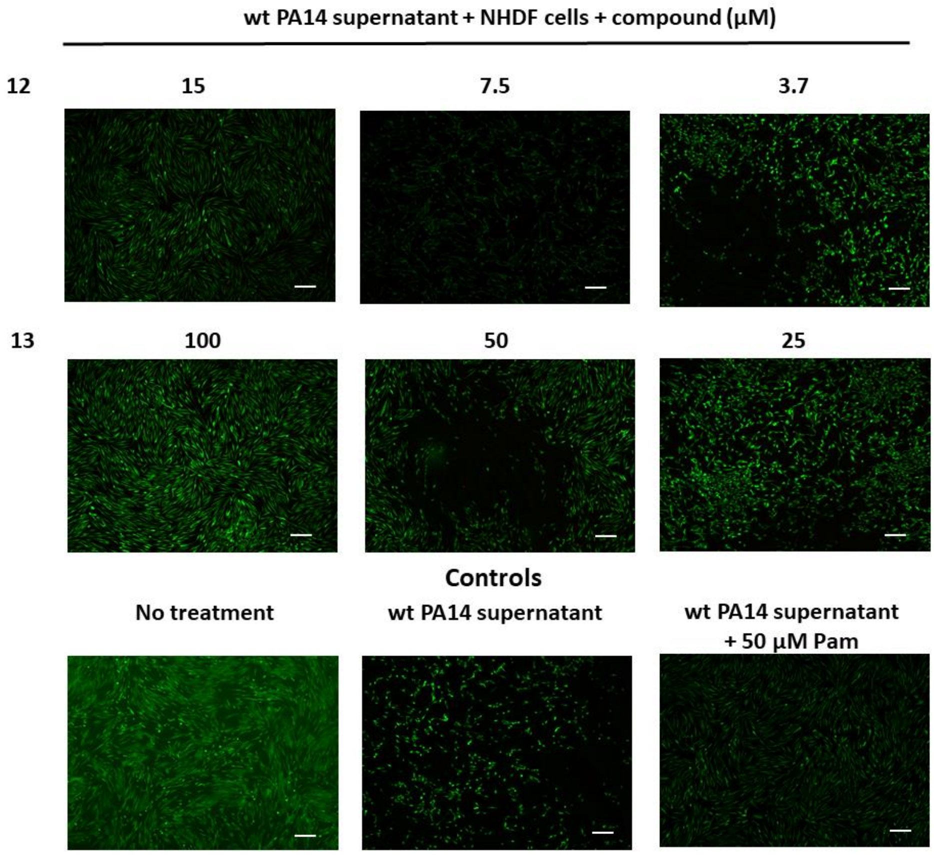

Figure S9. Visualization of the effects of compounds **12** and **13** on wt PA14 supernatant treated human dermal fibroblasts (NHDF) cells. Live/dead staining was carried out with fluoresceine diacetate and propidium iodine. Living cells are shown in green and dead cells in red (Scale bar: 200 µm). Red signal in some cases was lost because the detached cells were washed away after the rinsing step with PBS. wt PA14: wild-type *Pseudomonas aeruginosa*, Pam: phosphoramidon.

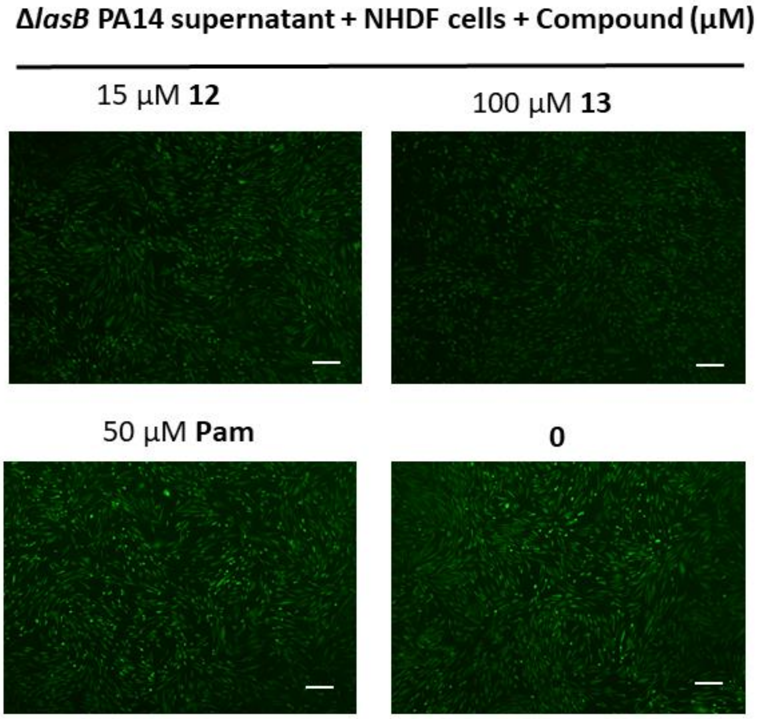

Figure S10. Visualization of effect of compounds **12** and **13** on Δ*lasB* PA14 supernatant applied human dermal fibroblasts (NHDF) cells. Live/dead staining was carried out with fluoresceine diacetate and propidium iodine. Live cells are shown in green and dead cells in red. Scale bar: 200 μm. Red signal in some cases was lost because the detached cells were washed away after the rinsing step with PBS. Δ*lasB* PA14: LasB knockout *Pseudomonas aeruginosa*, Pam: phosphoramidon.

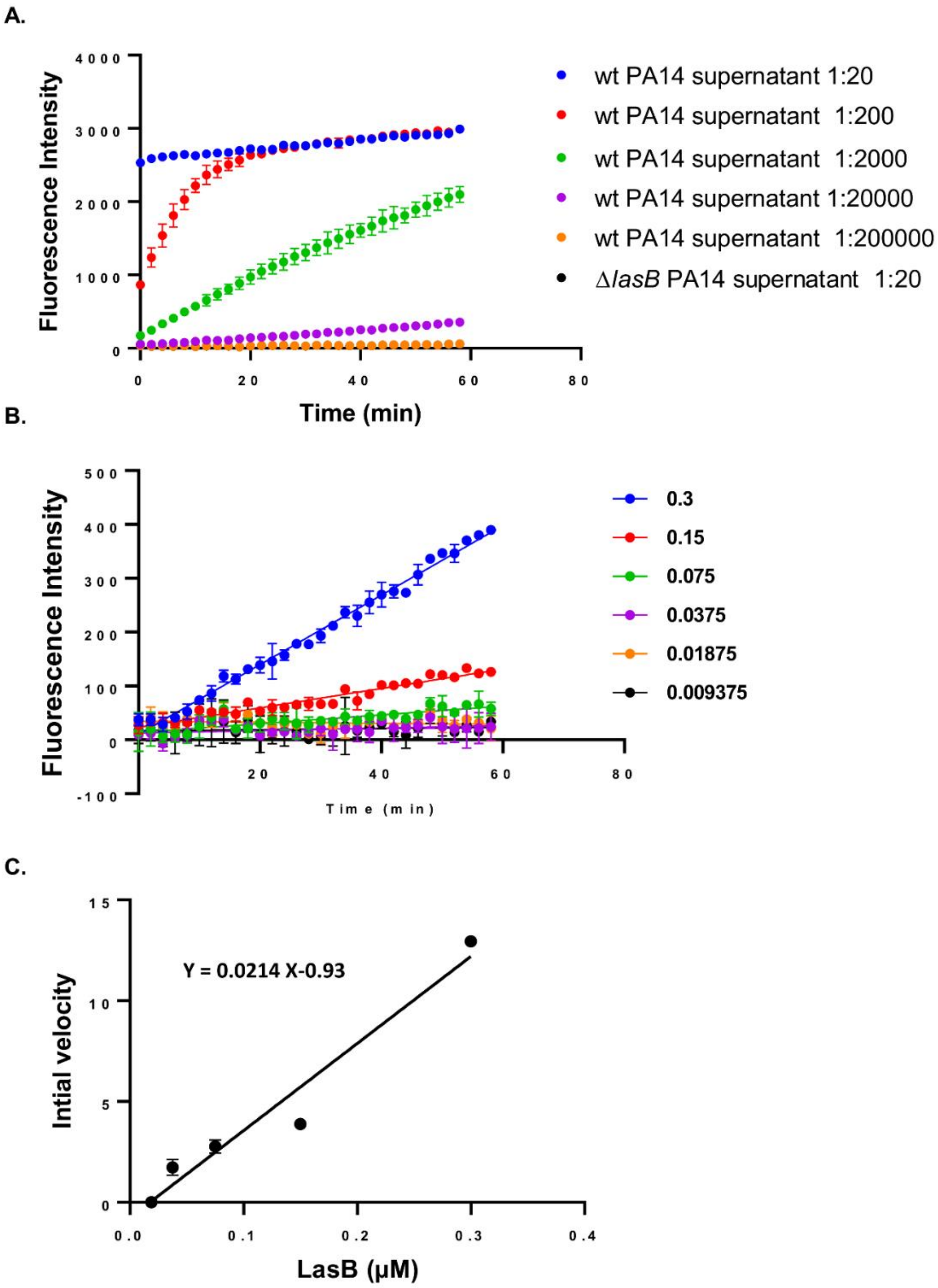

Figure S11. Supernatant evaluation with LasB activity assay. **A)** The activity of serially diluted wt PA14 and Δ*lasB* PA14 supernatants **B)** The activity of various concentrations of pure LasB. **C)** The calibration curve that was created from the initial velocity that we calculated from graph B. The calibration curve estimates that 100% supernatant has 0.88 μM of LasB. wt PA14: wild-type *Pseudomonas aeruginosa*, ΔlasB PA14: LasB knockout *Pseudomonas aeruginosa*.

NMR Spectra of Final Compounds

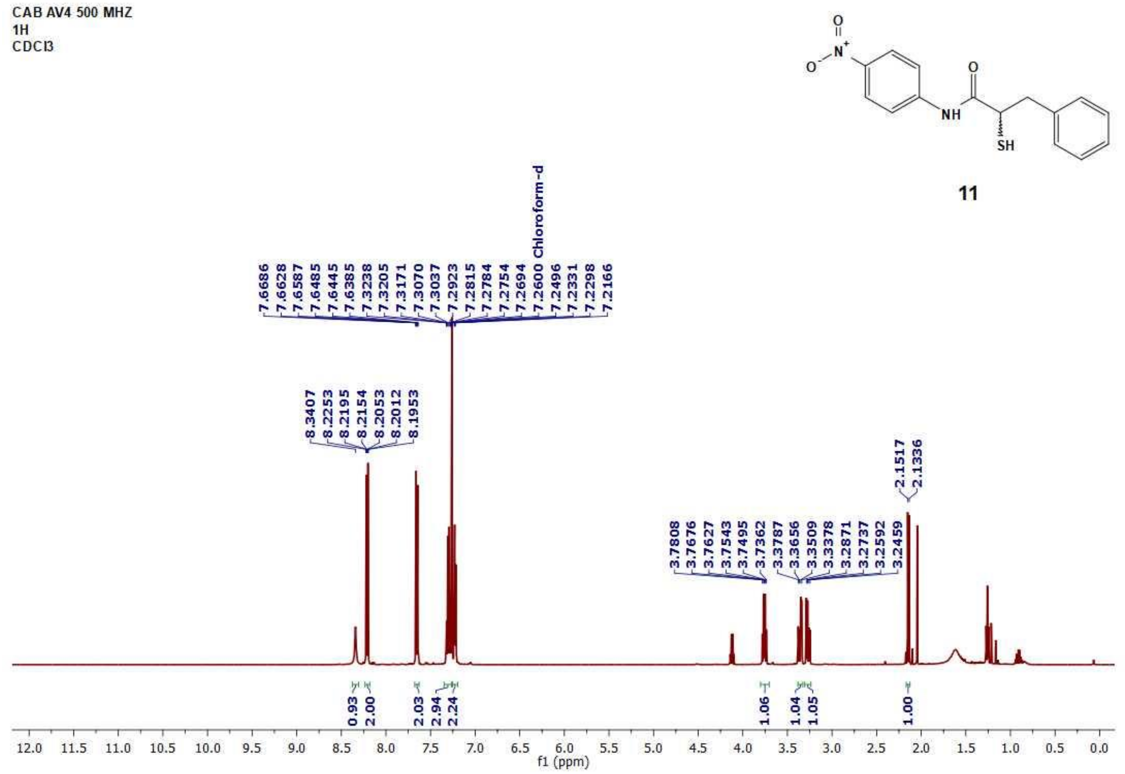

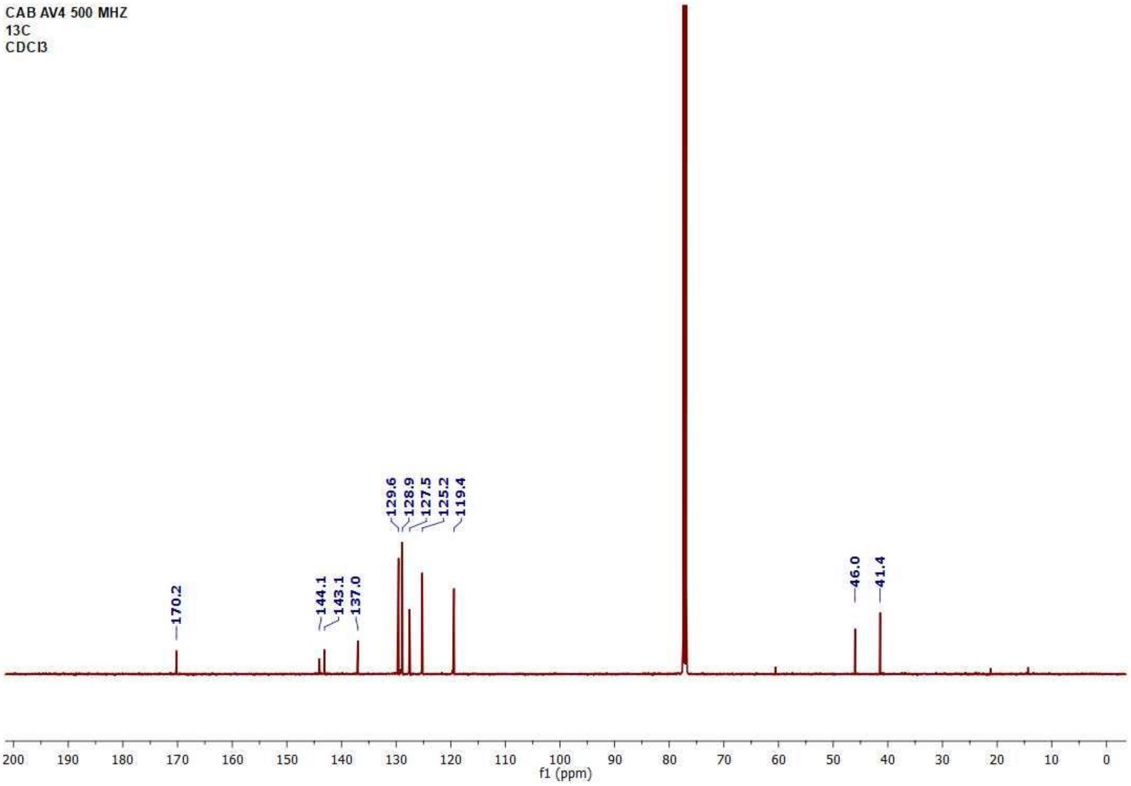

CAB AV4 500 MHZ
1H
CDCl3

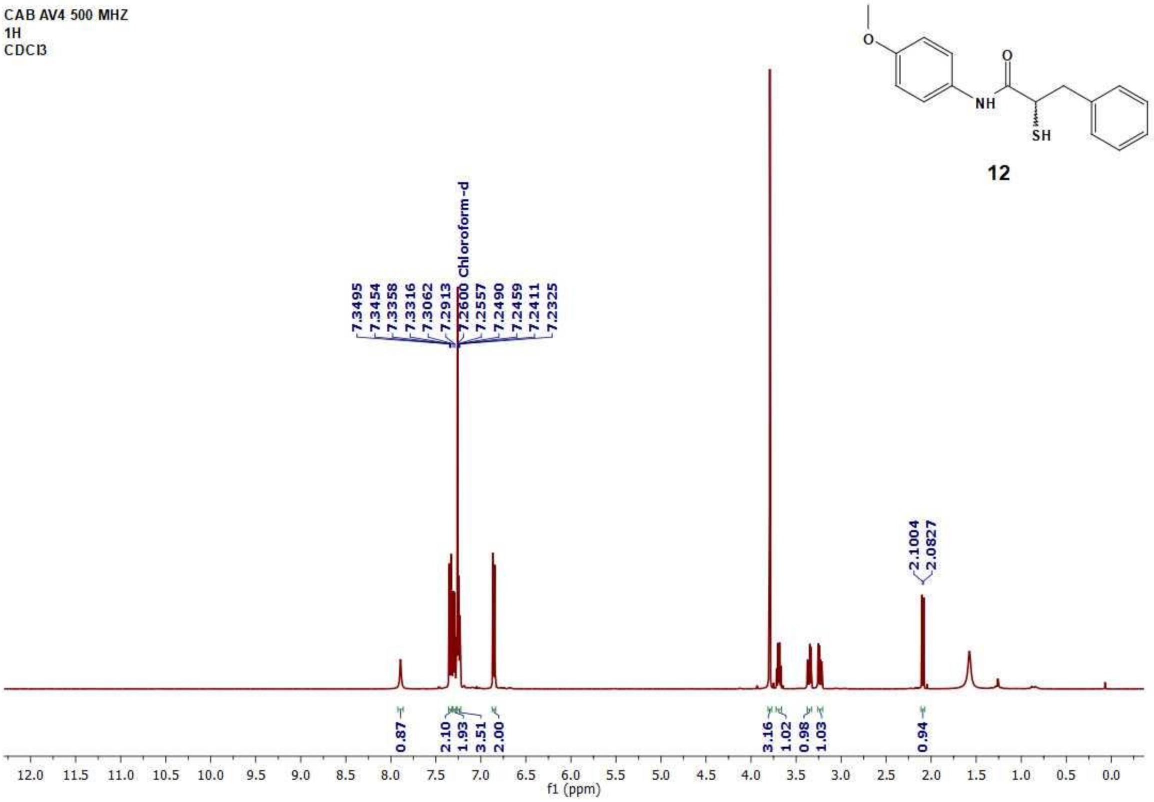

CAB AV4 500 MHZ
13C
CDCl3

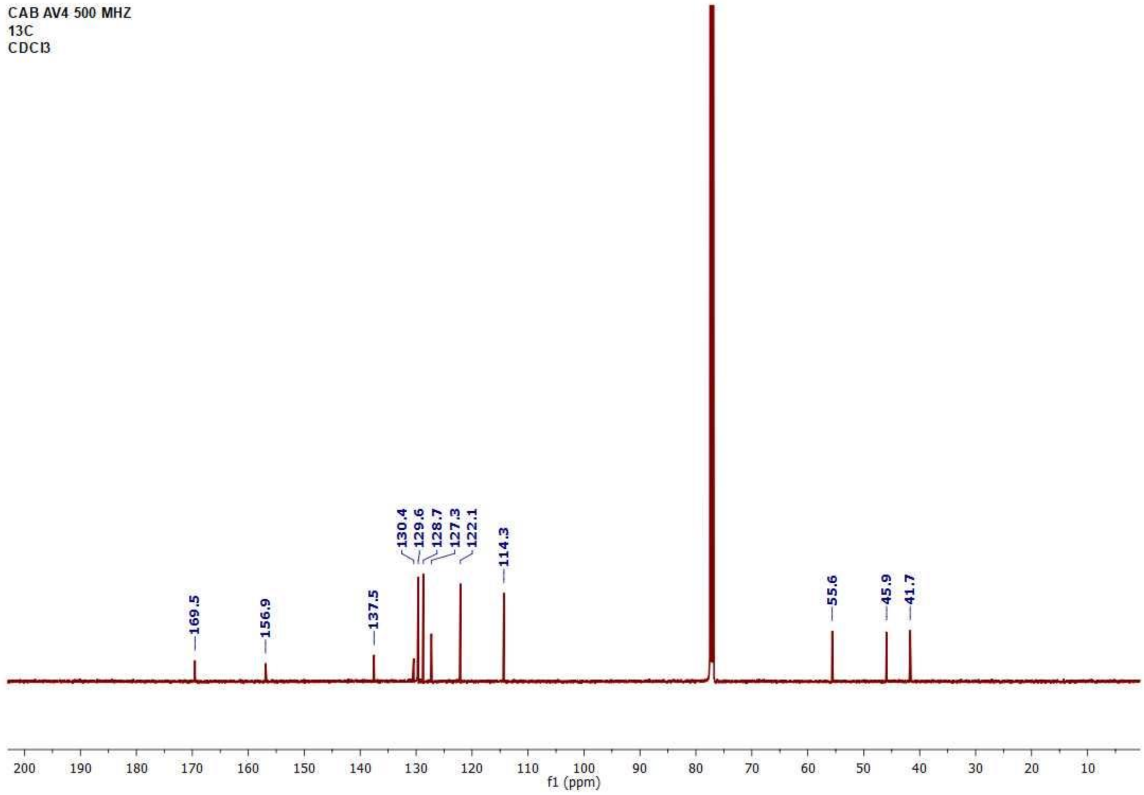

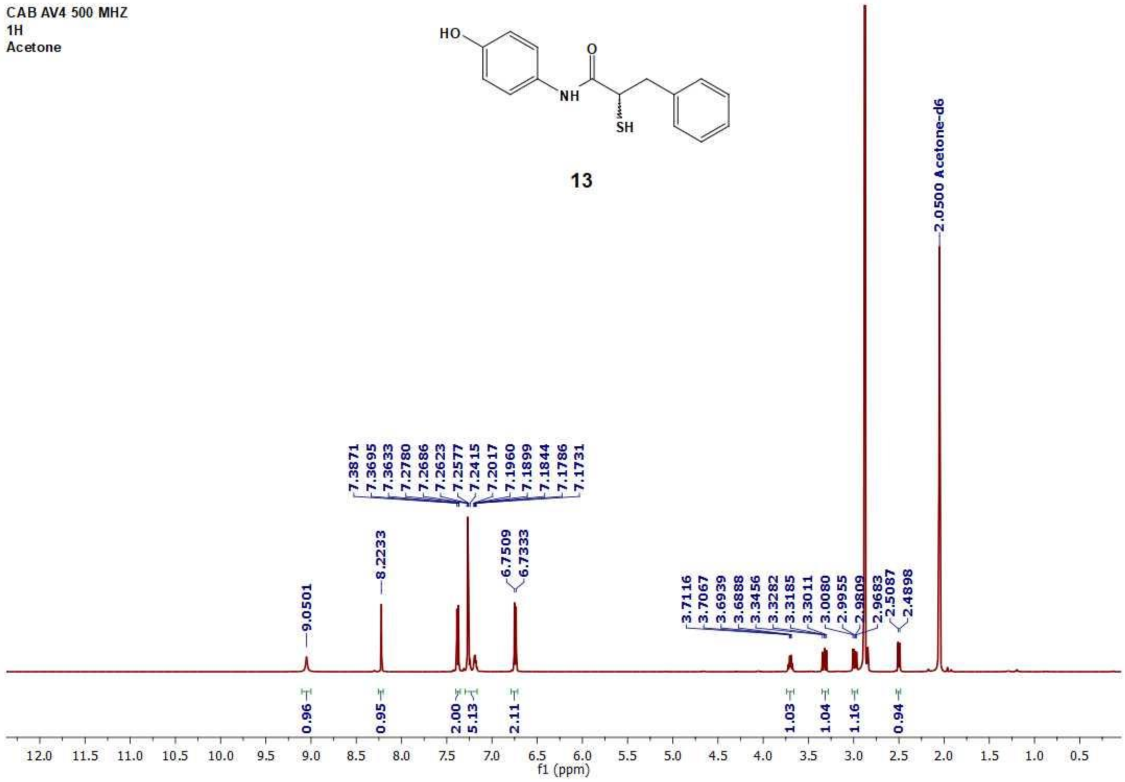
CAB AV4 500 MHZ
1H
Acetone
HO
O
NH
SH
13
2.0500 Acetone-d6
7.3871
7.3695
7.3633
7.2780
7.2686
7.2623
7.2577
7.2415
7.2017
7.1960
7.1899
7.1844
7.1786
7.1731
8.2233
6.7509
6.7333
9.0501
3.7116
3.7067
3.6939
3.6888
3.3456
3.3282
3.3185
3.3011
3.0080
2.9955
2.9809
2.9683
2.5087
2.4898
0.96
0.95
2.00
5.13
2.11
1.03
1.04
1.16
0.94
f1 (ppm)

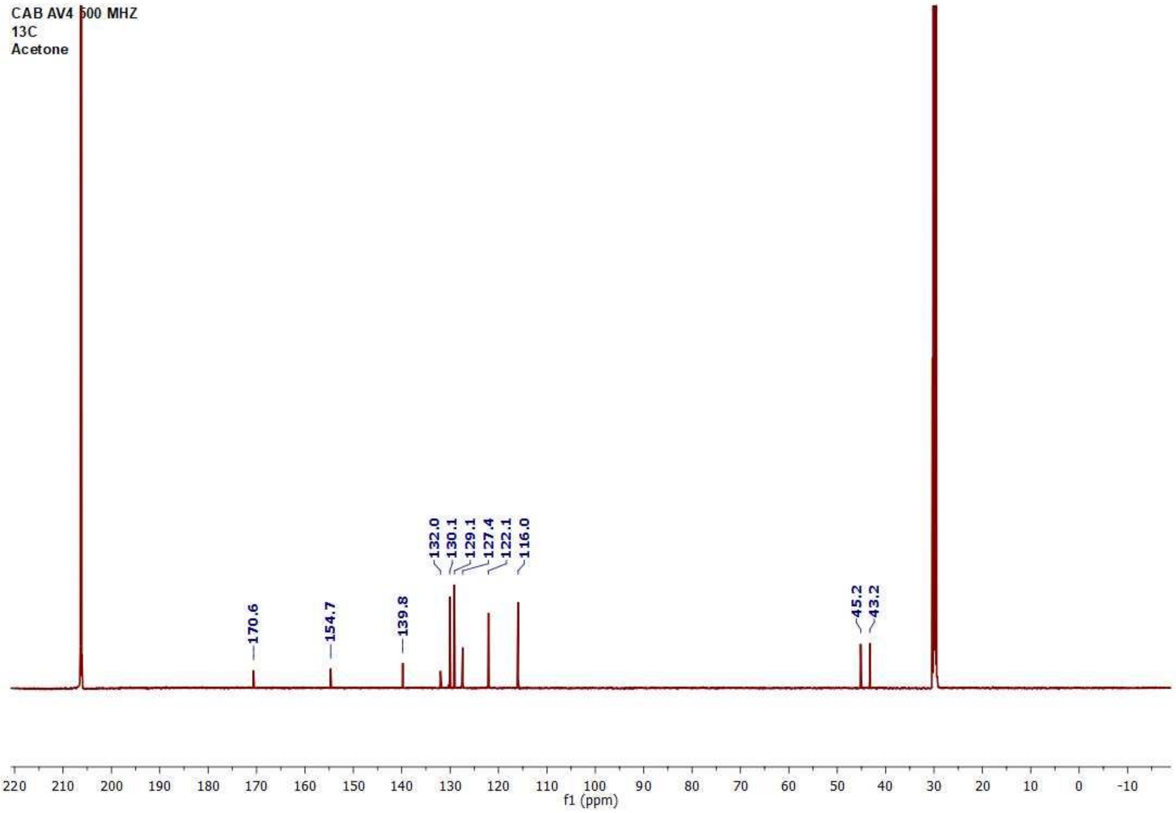
CAB AV4 500 MHZ
13C
Acetone
170.6
154.7
139.8
132.0
130.1
129.1
127.4
122.1
116.0
45.2
43.2
f1 (ppm)

CAB AV4 500 MHZ
1H
CDCl3

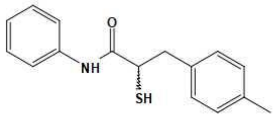

14

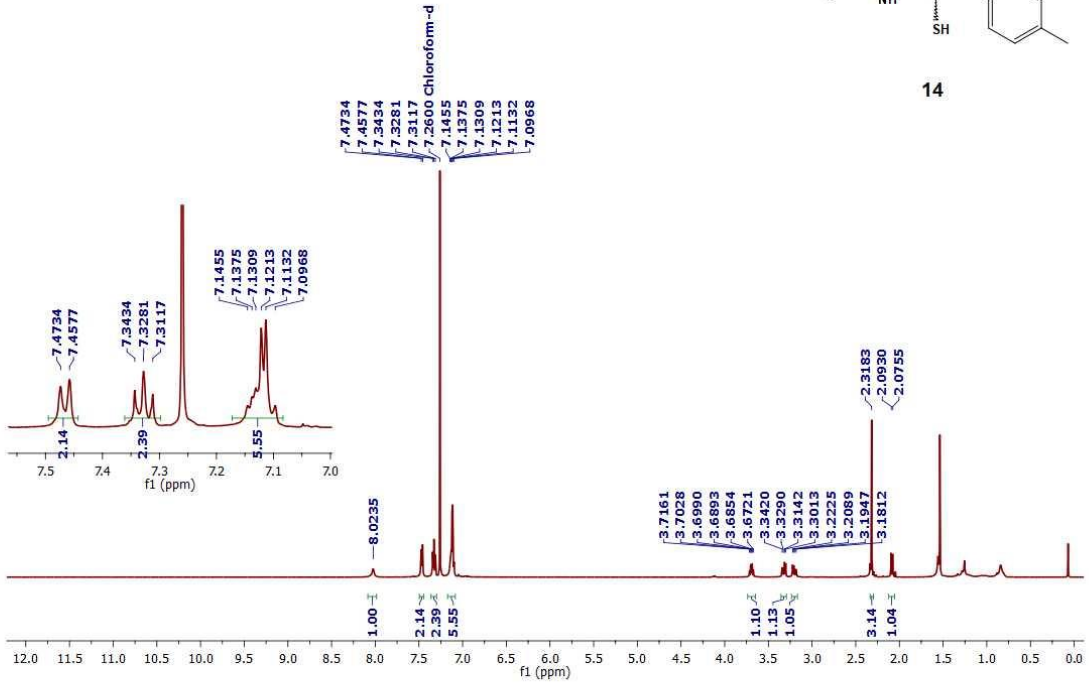

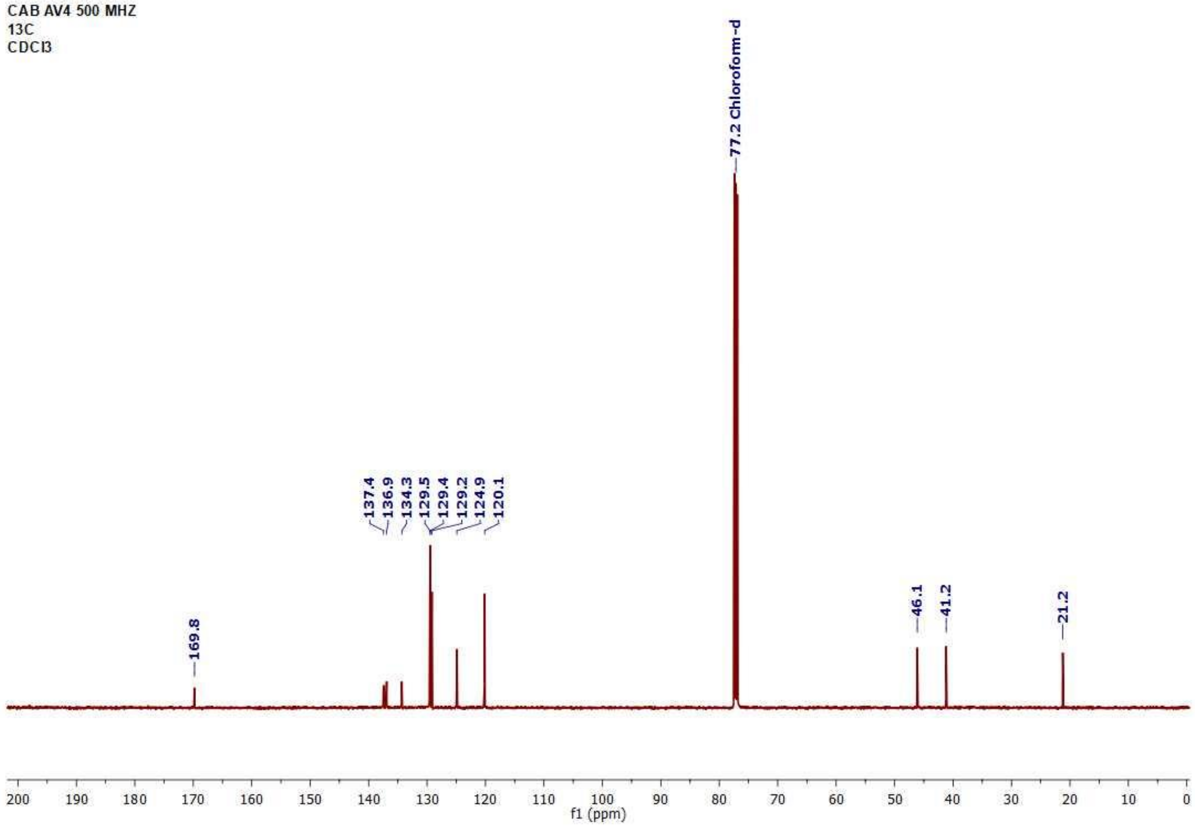

CAB AV4 500 MHZ
1H
Acetone

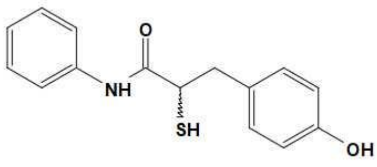

15

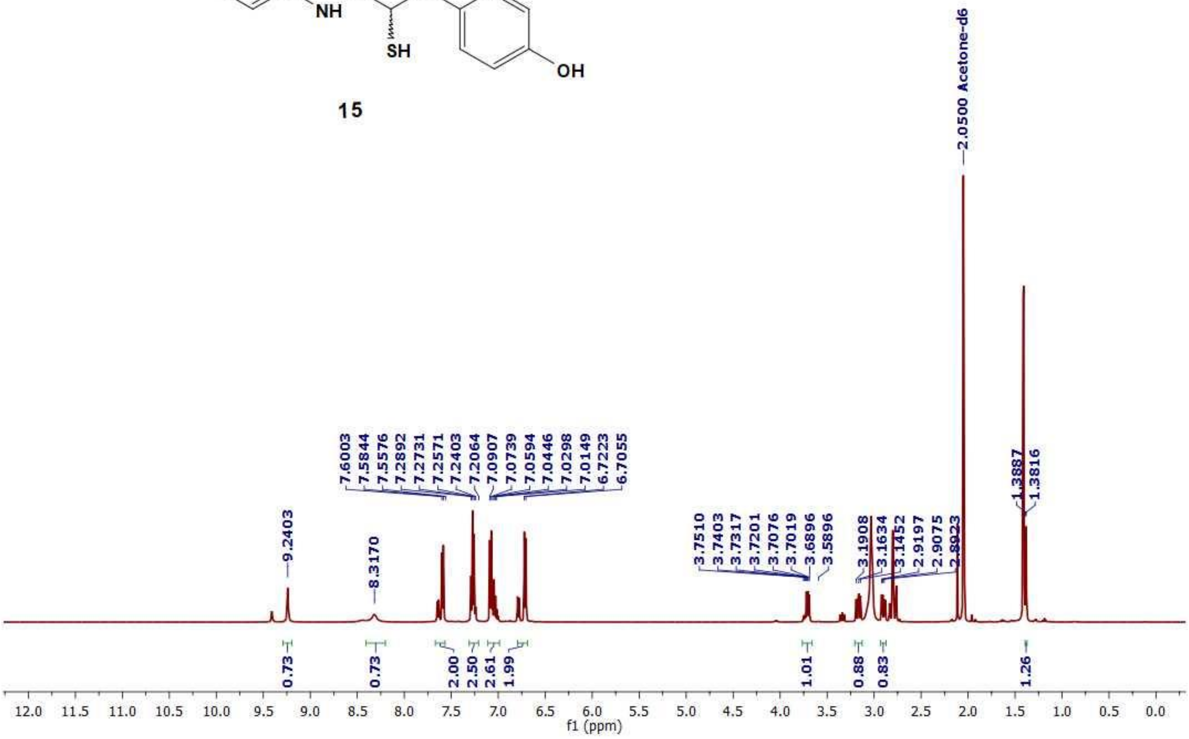

CAB AV4 500 MHZ
13C
DMSO

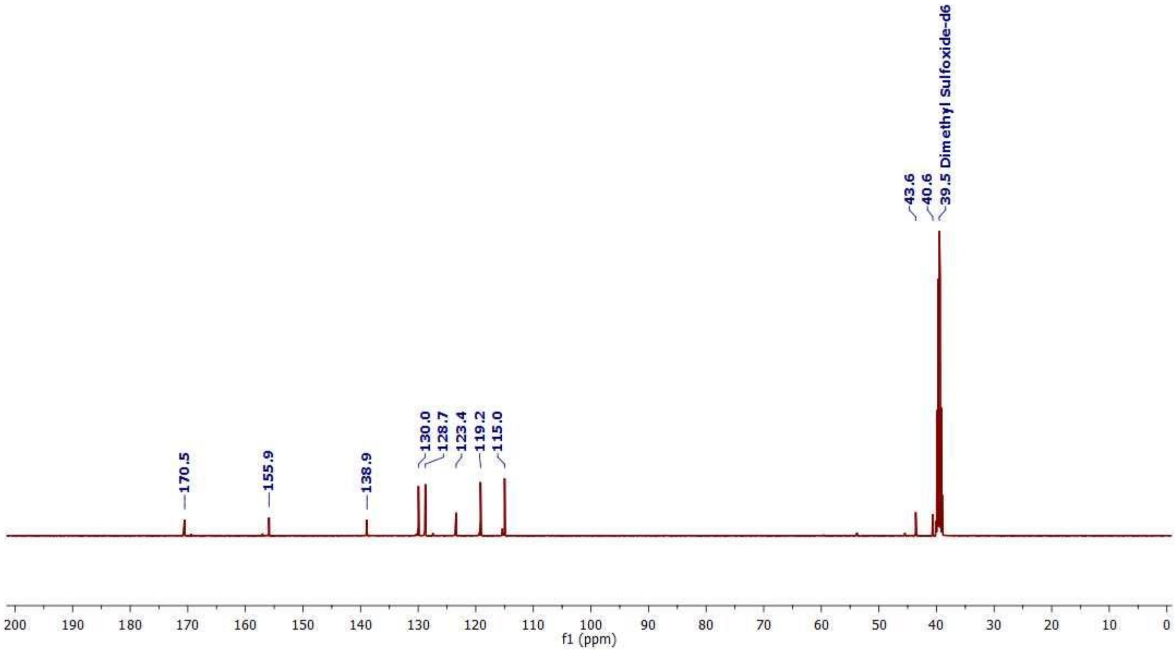

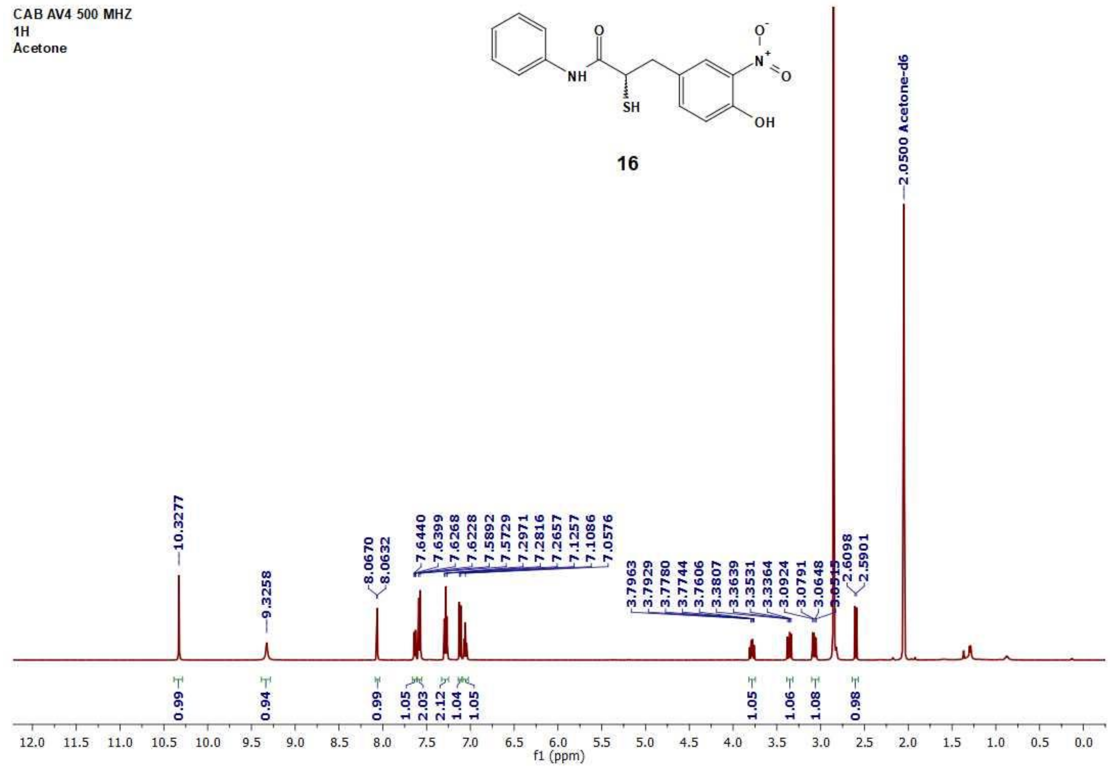
CAB AV4 500 MHZ
1H
Acetone
16
10.3277
9.3258
8.0670
8.0632
7.6440
7.6399
7.6268
7.6228
7.5892
7.5729
7.2971
7.2816
7.2657
7.1257
7.1086
7.0576
3.7963
3.7929
3.7780
3.7744
3.7606
3.3807
3.3639
3.3531
3.3364
3.0924
3.0791
3.0648
2.6098
2.5901
2.0500 Acetone-d6
0.99
0.94
0.99
1.05
2.03
2.12
1.04
1.05
1.05
1.06
1.08
0.98
f1 (ppm)

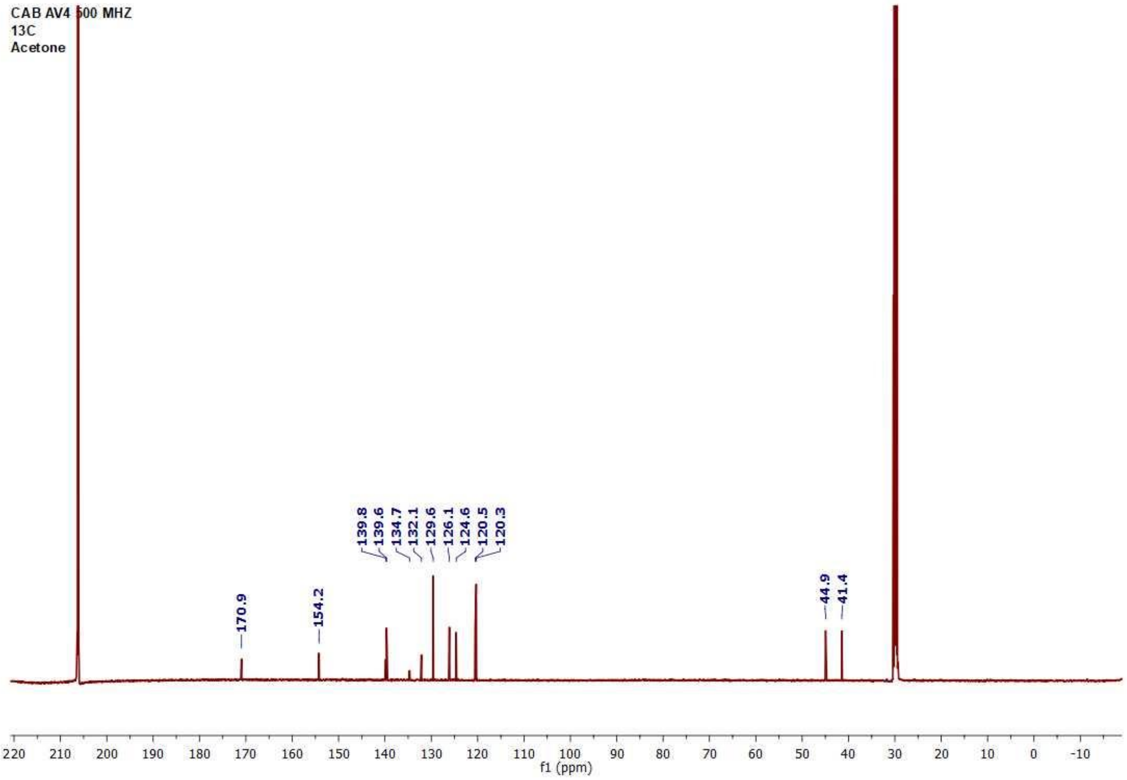
CAB AV4 500 MHZ
13C
Acetone
170.9
154.2
139.8
139.6
134.7
132.1
129.6
126.1
124.6
120.5
120.3
44.9
41.4
f1 (ppm)

CAB AV4 500 MHZ
1H
Acetone

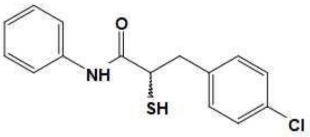

17

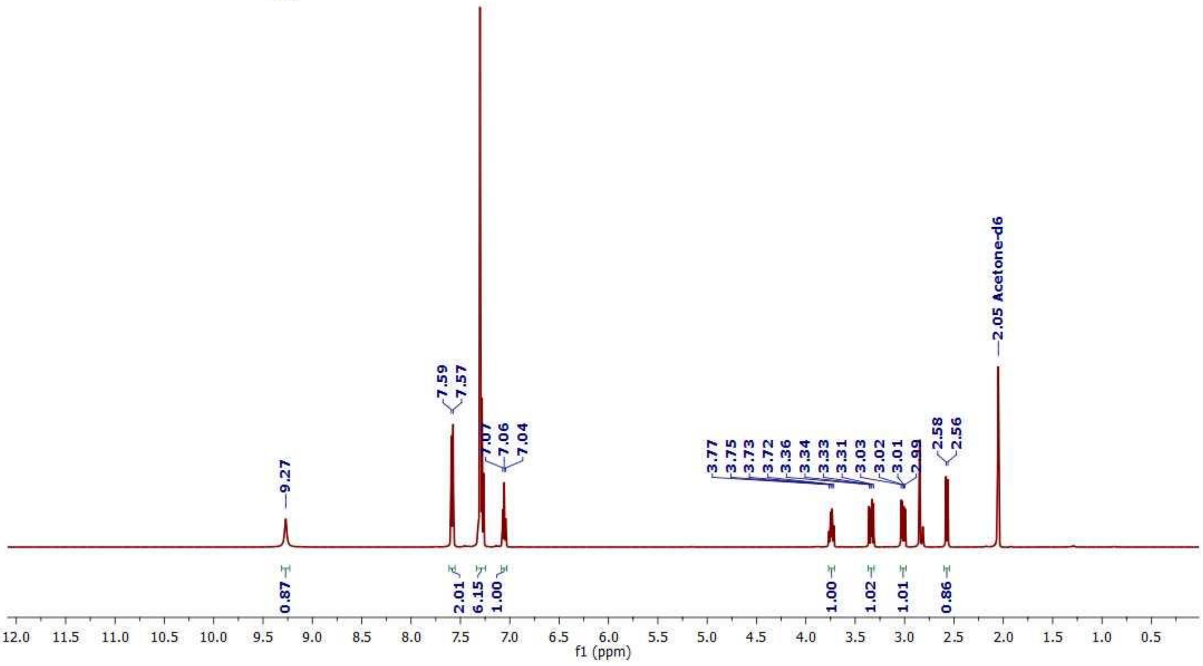

CAB AV4 500 MHZ
13C
DMSO

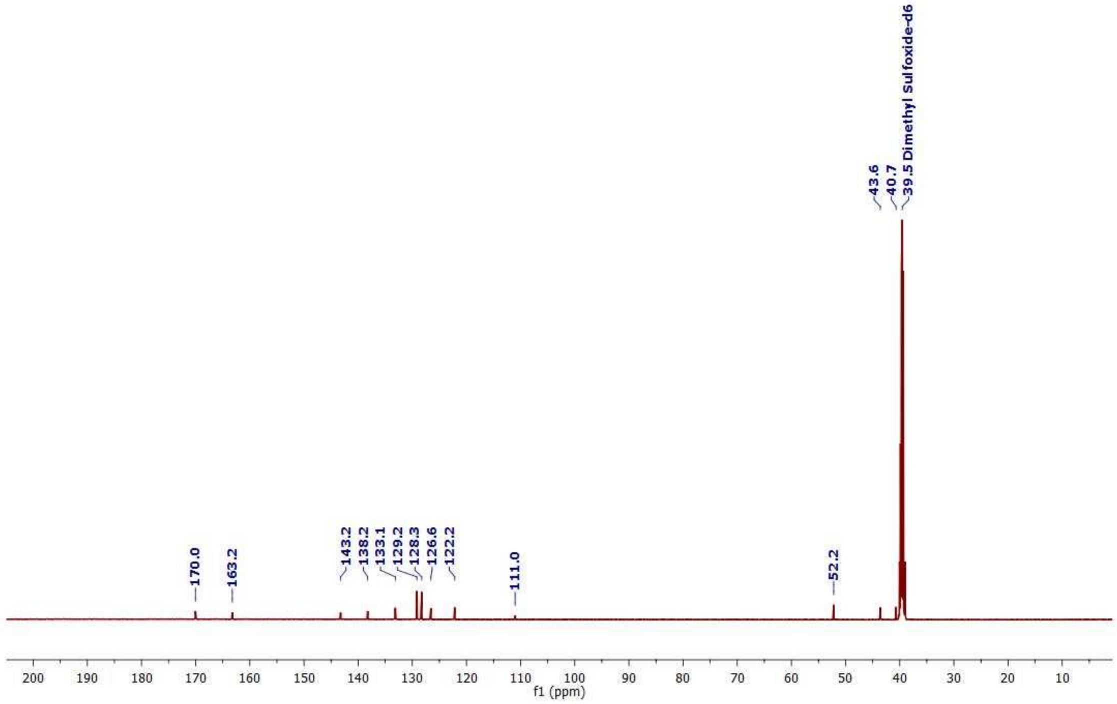

CAB AV4 500 MHZ
1H
DMSO

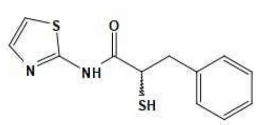

18

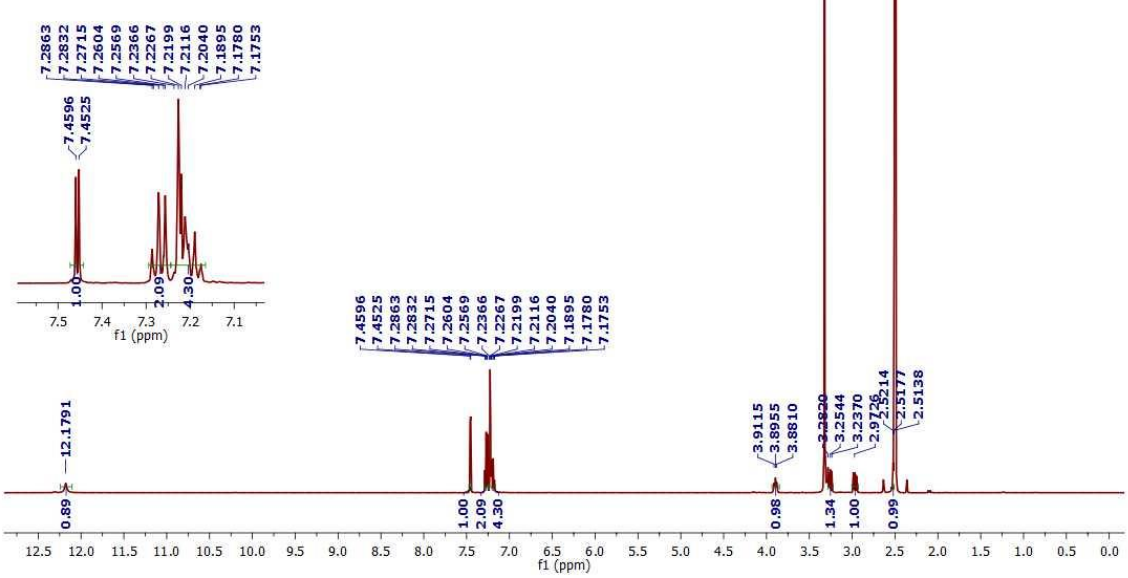

CAB AV4 500 MHZ
13C
DMSO

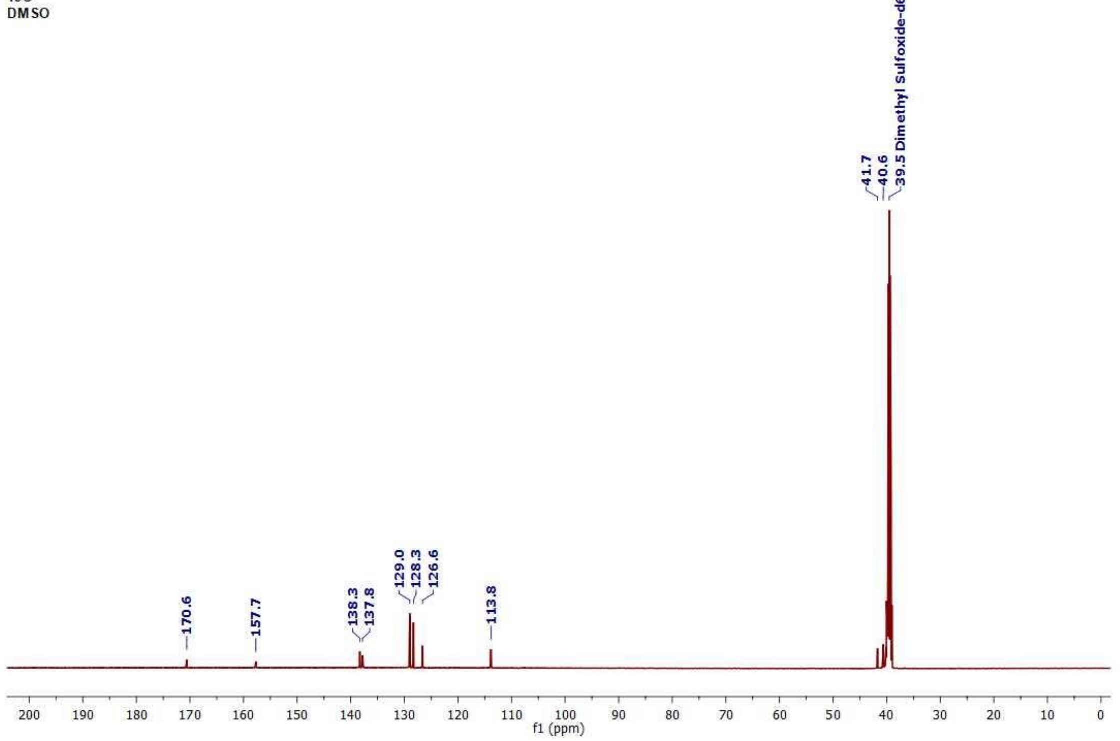

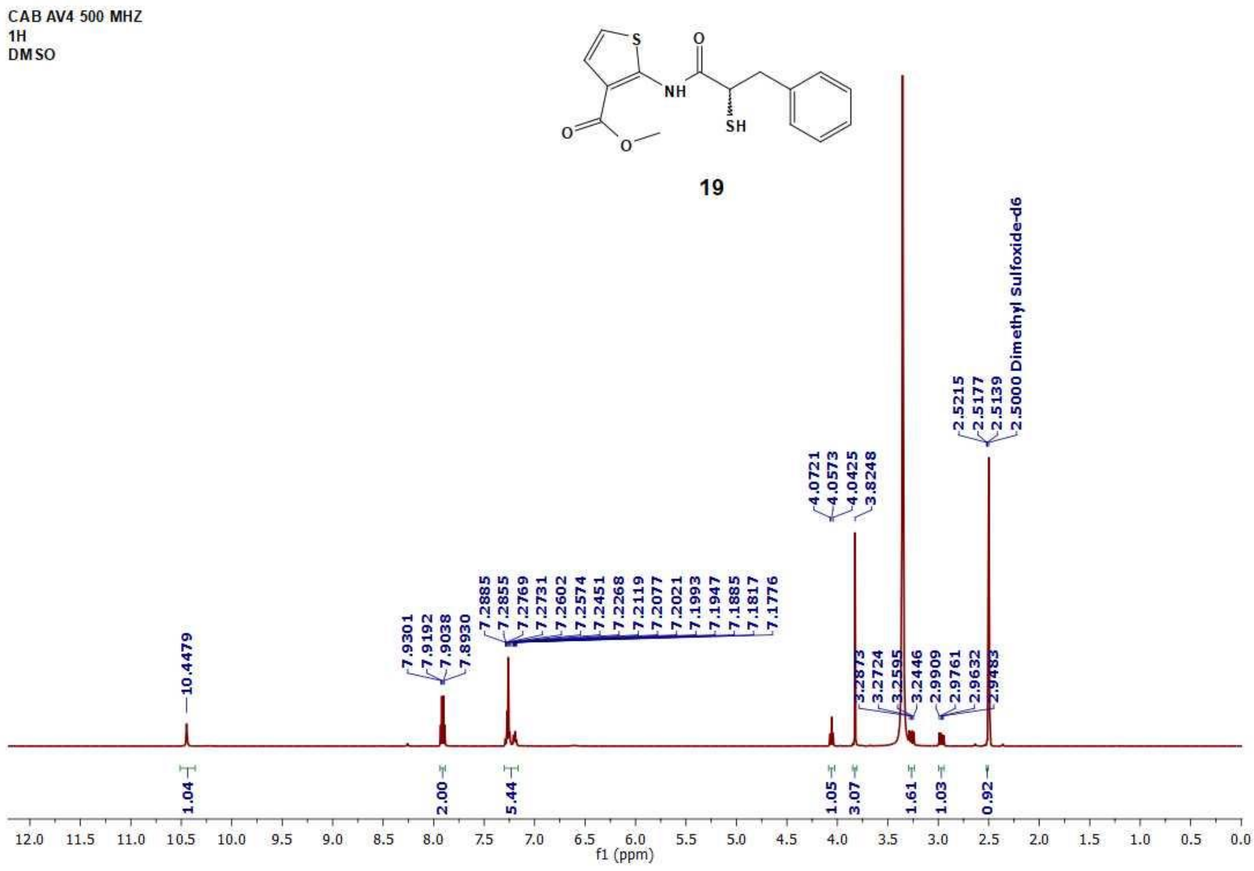
CAB AV4 500 MHZ
1H
DMSO
S
O
NH
SH
O
O
19
10.4479
7.9301
7.9192
7.9038
7.8930
7.2885
7.2855
7.2769
7.2731
7.2602
7.2574
7.2451
7.2268
7.2119
7.2077
7.2021
7.1993
7.1947
7.1885
7.1817
7.1776
4.0721
4.0573
4.0425
3.8248
3.2873
3.2724
3.2595
3.2446
2.9909
2.9761
2.9632
2.9483
2.5215
2.5177
2.5139
2.5000 Dimethyl Sulfoxide-d6
1.04
2.00
5.44
1.05
3.07
1.61
1.03
0.92
12.0 11.5 11.0 10.5 10.0 9.5 9.0 8.5 8.0 7.5 7.0 6.5 6.0 5.5 5.0 4.5 4.0 3.5 3.0 2.5 2.0 1.5 1.0 0.5 0.0
f1 (ppm)

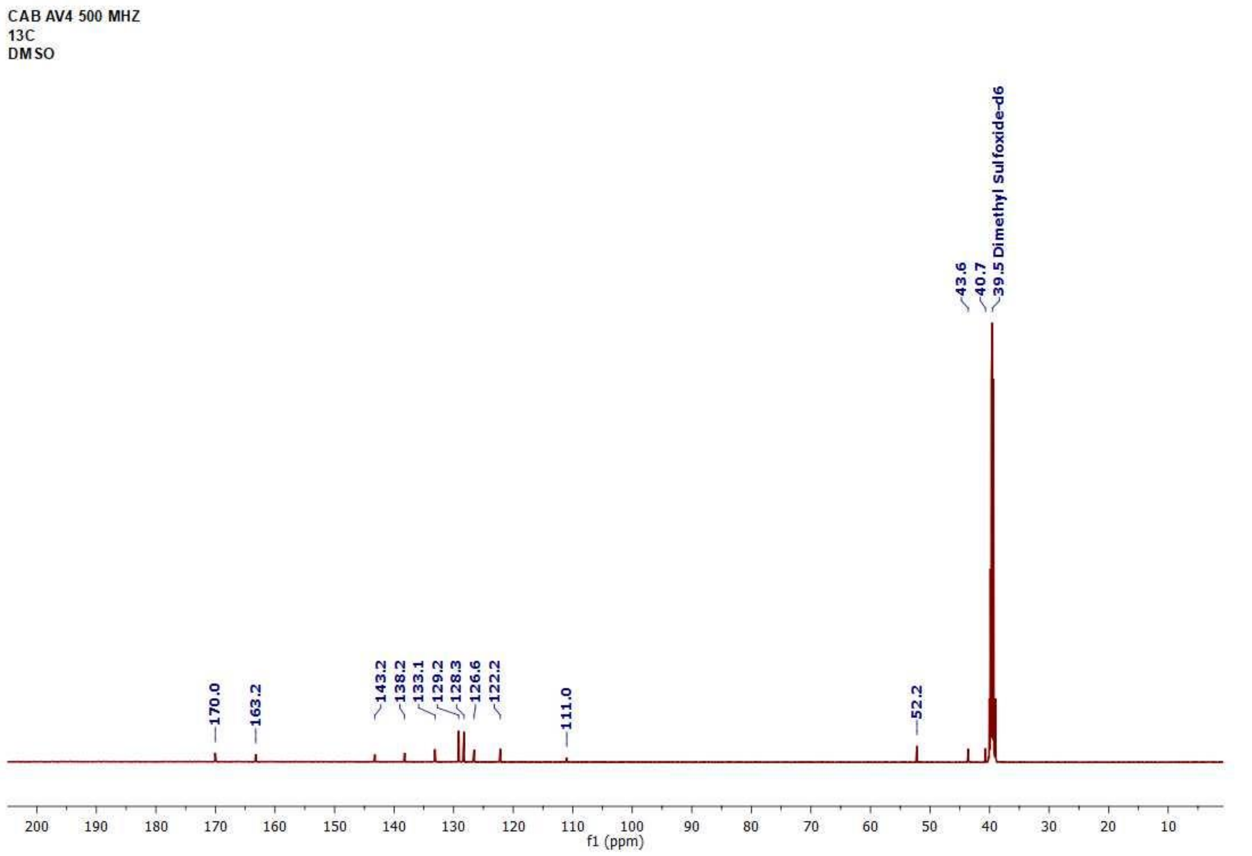
CAB AV4 500 MHZ
13C
DMSO
170.0
163.2
143.2
138.2
133.1
129.2
128.3
126.6
122.2
111.0
52.2
43.6
40.7
39.5 Dimethyl Sulfoxide-d6
200 190 180 170 160 150 140 130 120 110 100 90 80 70 60 50 40 30 20 10
f1 (ppm)

CAB AV4 500 MHZ
1H
DMSO

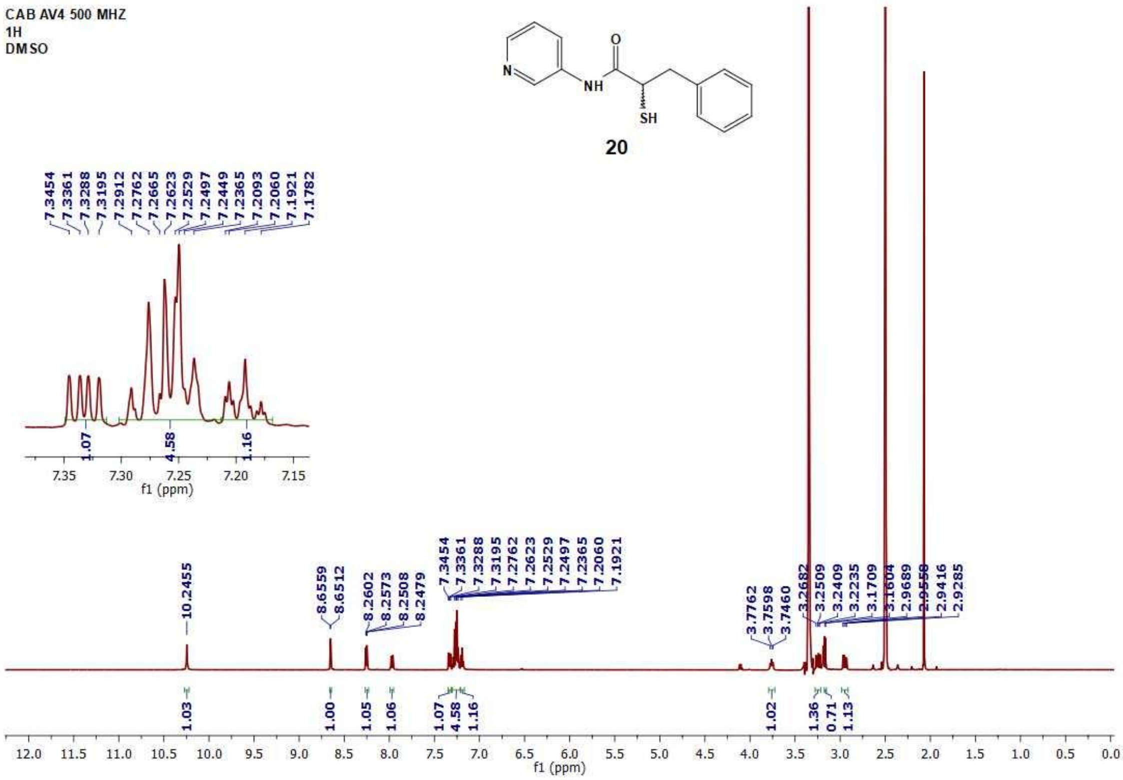

CAB AV4 500 MHZ
13C
DMSO

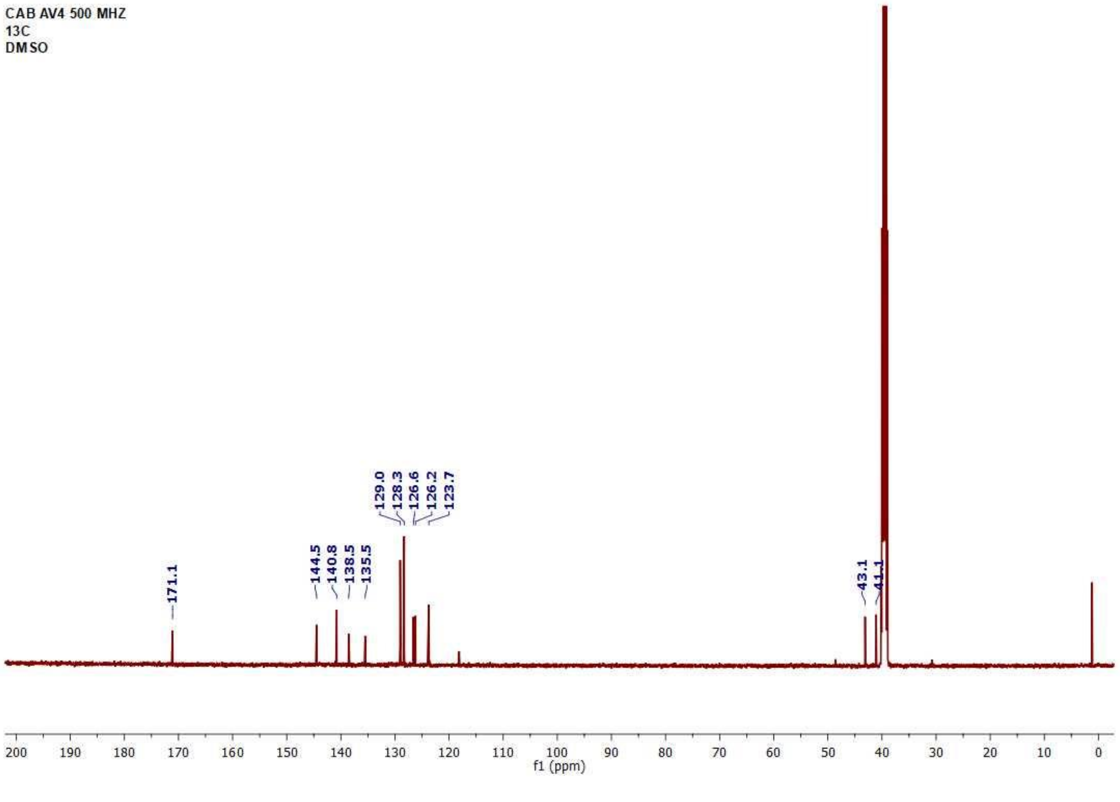

CAB AV4 500 MHZ
1H
Acetone

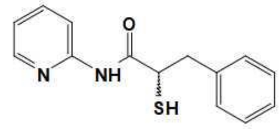

21

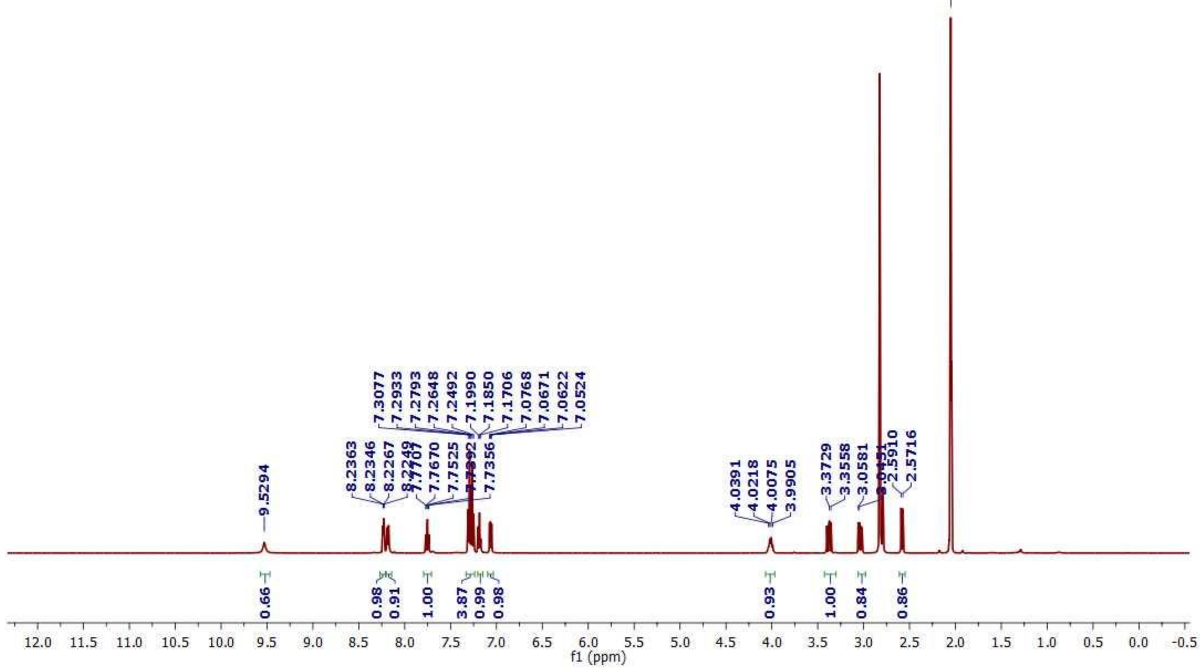

CAB AV4 500 MHZ
13C
Acetone

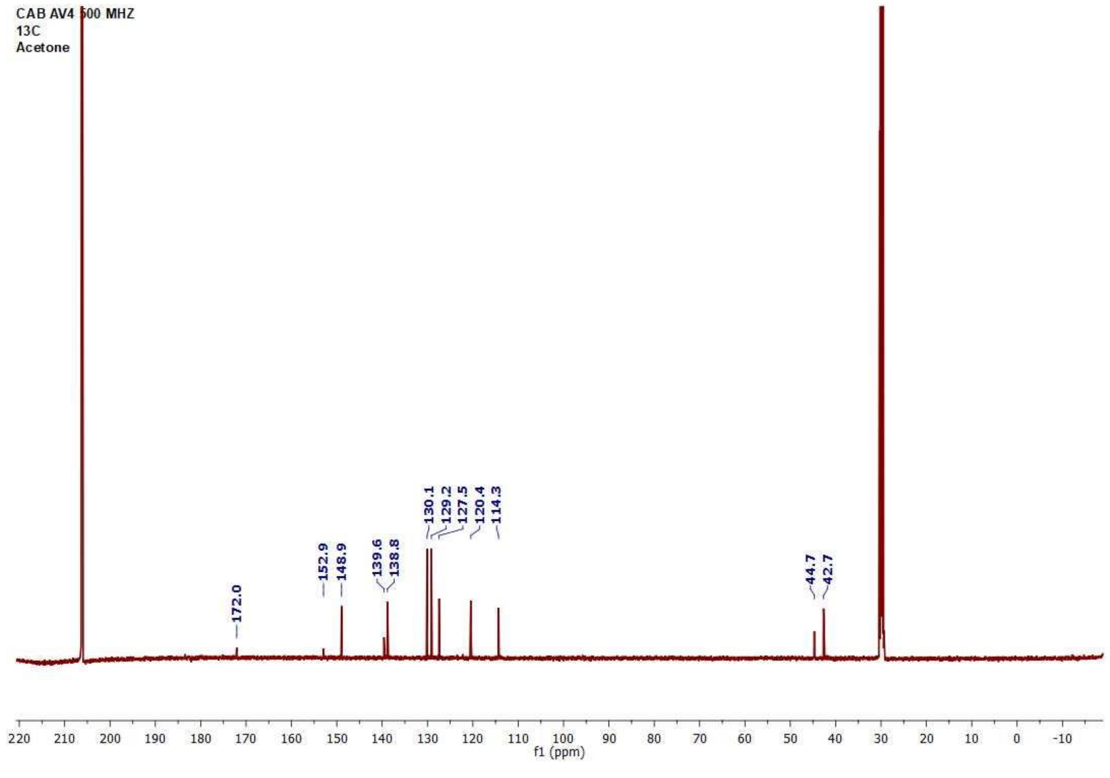

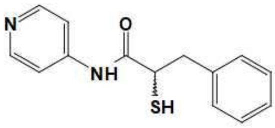

22

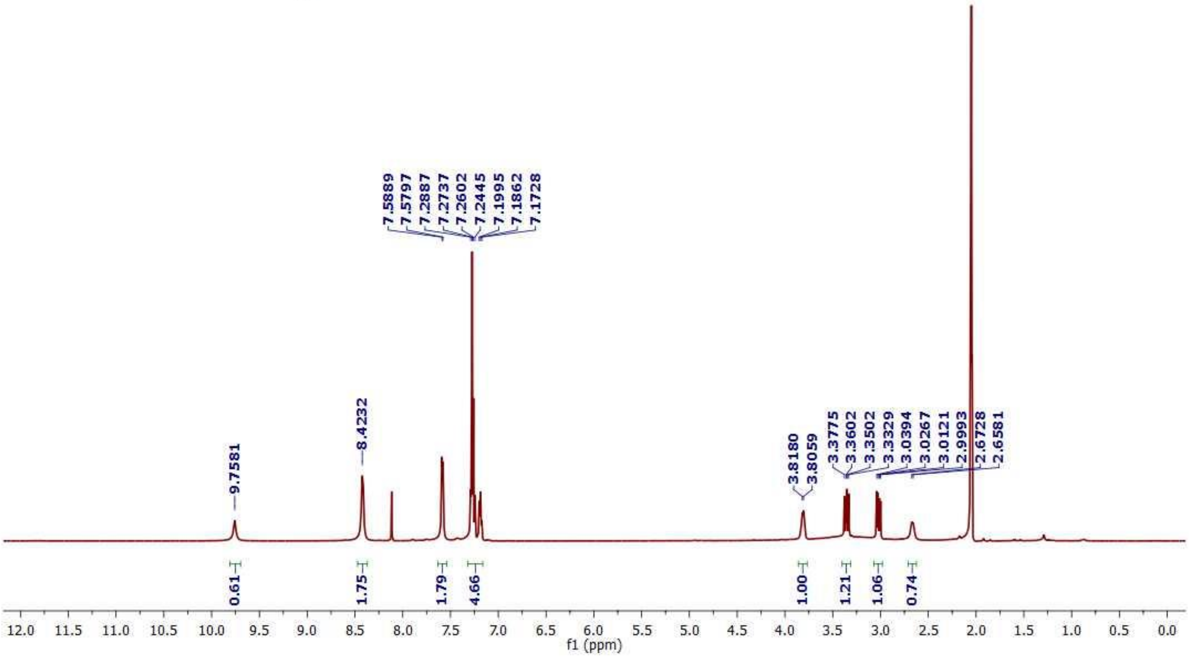

CAB AV4 500 MHZ
1H
Acetone
2.0500 Acetone-d6
7.5889
7.5797
7.2887
7.2737
7.2602
7.2445
7.1995
7.1862
7.1728
9.7581
8.4232
3.8180
3.8059
3.3775
3.3602
3.3502
3.3329
3.0394
3.0267
3.0121
2.9993
2.6728
2.6581
0.61
1.75
1.79
4.66
1.00
1.21
1.06
0.74
f1 (ppm)

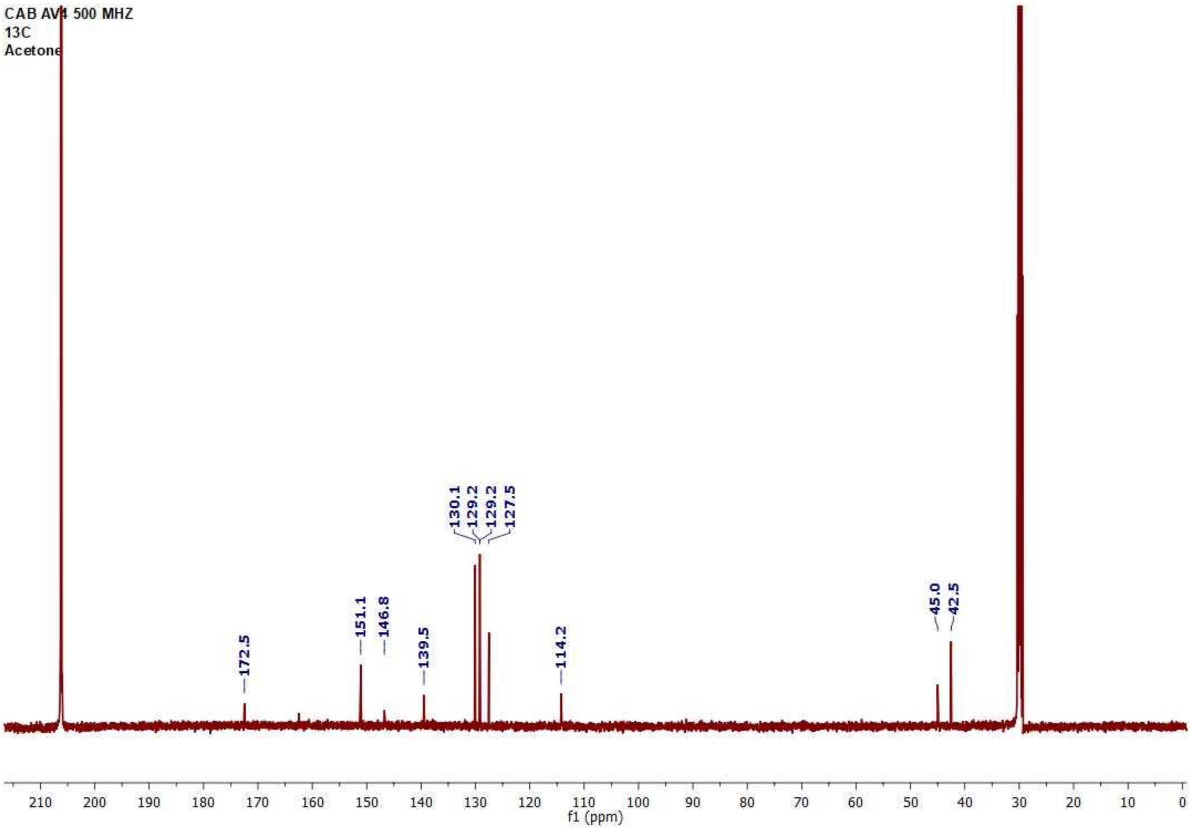

CAB AV4 500 MHZ
13C
Acetone
130.1
129.2
129.2
127.5
172.5
151.1
146.8
139.5
114.2
45.0
42.5
f1 (ppm)

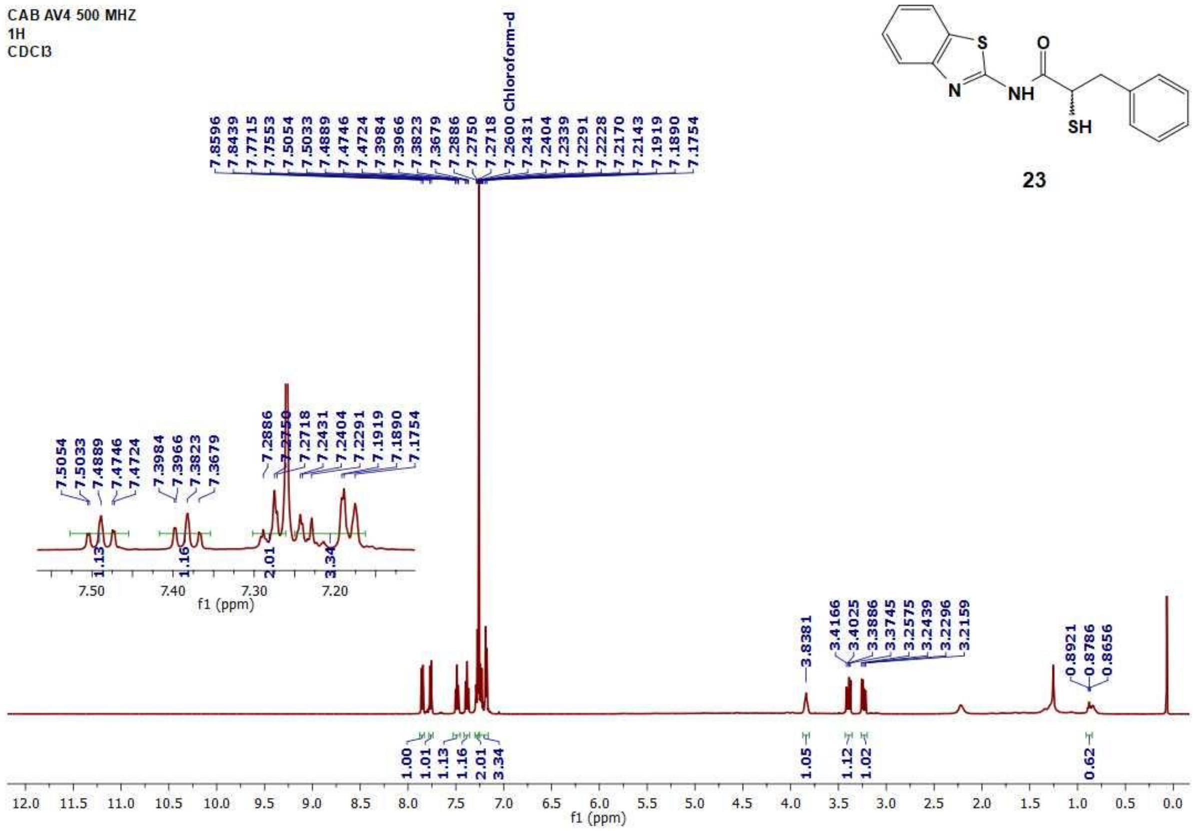
CAB AV4 500 MHZ
1H
CDCl3
7.8596 7.8439 7.7715 7.7553 7.5054 7.5033 7.4889 7.4746 7.4724 7.3984 7.3966 7.3823 7.3679 7.2886 7.2750 7.2718 7.2600 Chloroform-d 7.2431 7.2404 7.2339 7.2291 7.2228 7.2170 7.2143 7.1919 7.1890 7.1754
7.5054 7.5033 7.4889 7.4746 7.4724 7.3984 7.3966 7.3823 7.3679 7.2886 7.2750 7.2718 7.2431 7.2404 7.2291 7.1919 7.1890 7.1754
1.13 1.16 2.01 3.34
f1 (ppm)
3.8381
3.4166 3.4025 3.3886 3.3745 3.2575 3.2439 3.2296 3.2159
0.8921 0.8786 0.8656
1.00 1.01 1.13 1.16 2.01 3.34 1.05 1.12 1.02 0.62
f1 (ppm)
S
N
NH
O
SH

23

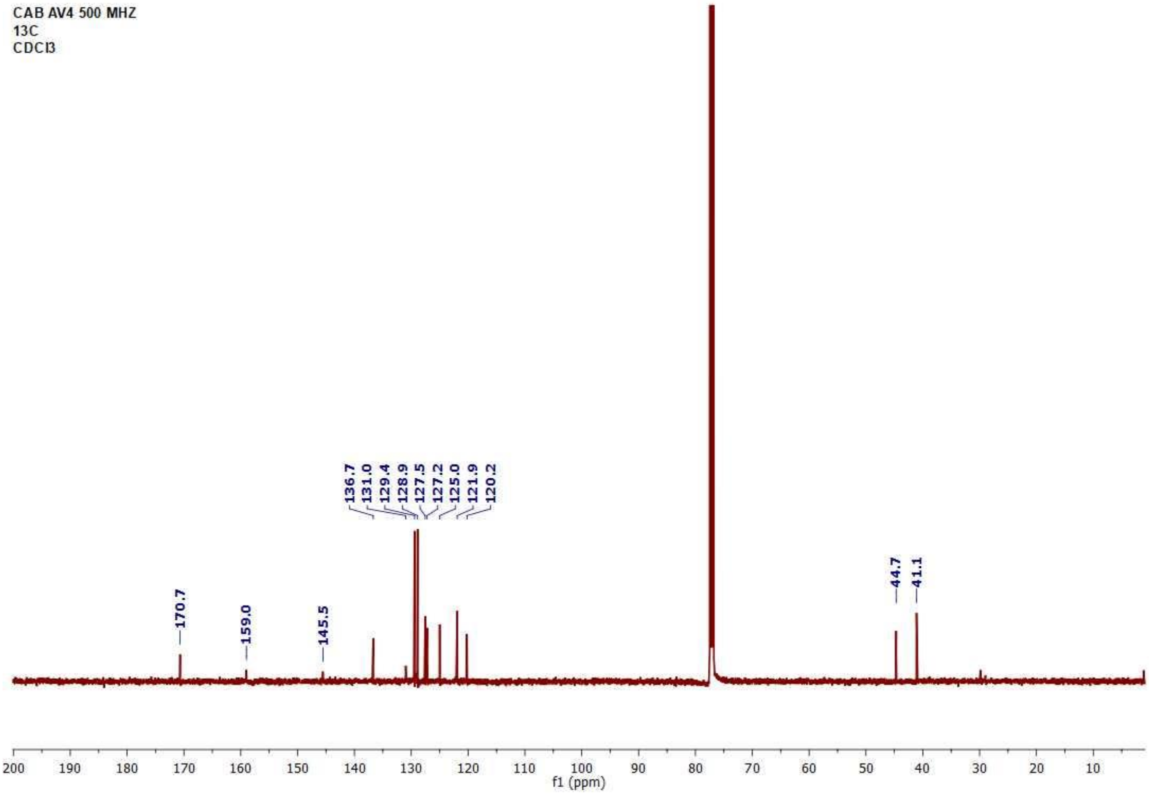
CAB AV4 500 MHZ
13C
CDCl3
170.7
159.0
145.5
136.7 131.0 129.4 128.9 127.5 127.2 125.0 121.9 120.2
44.7
41.1
f1 (ppm)

CAB AV4 500 MHZ
1H
DMSO

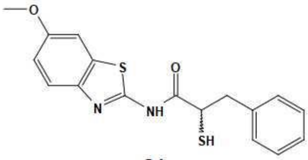

24

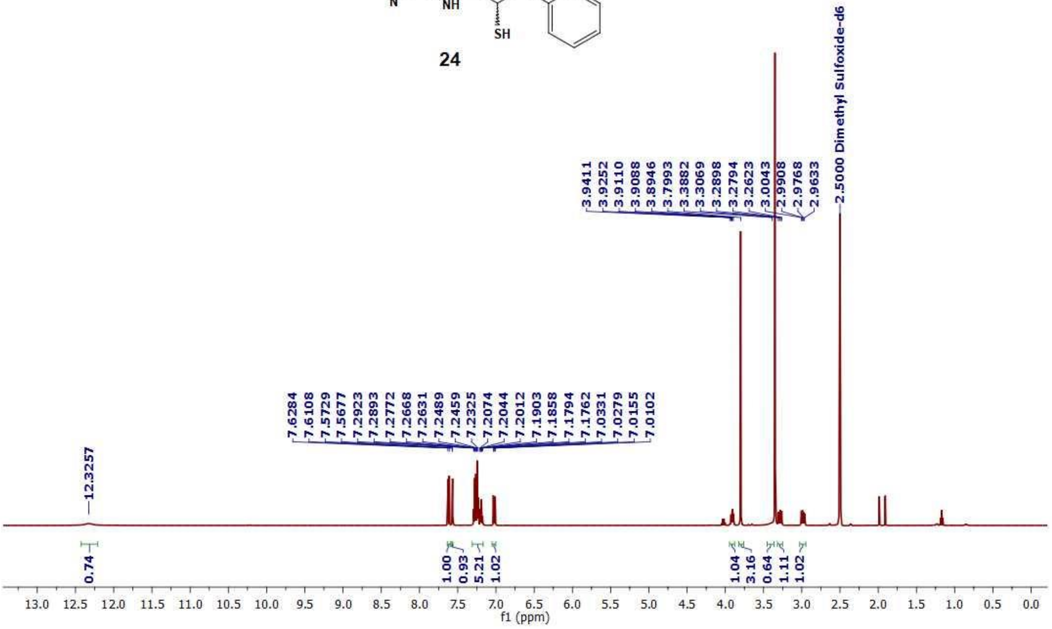

CAB AV4 500 MHZ
13C
DMSO

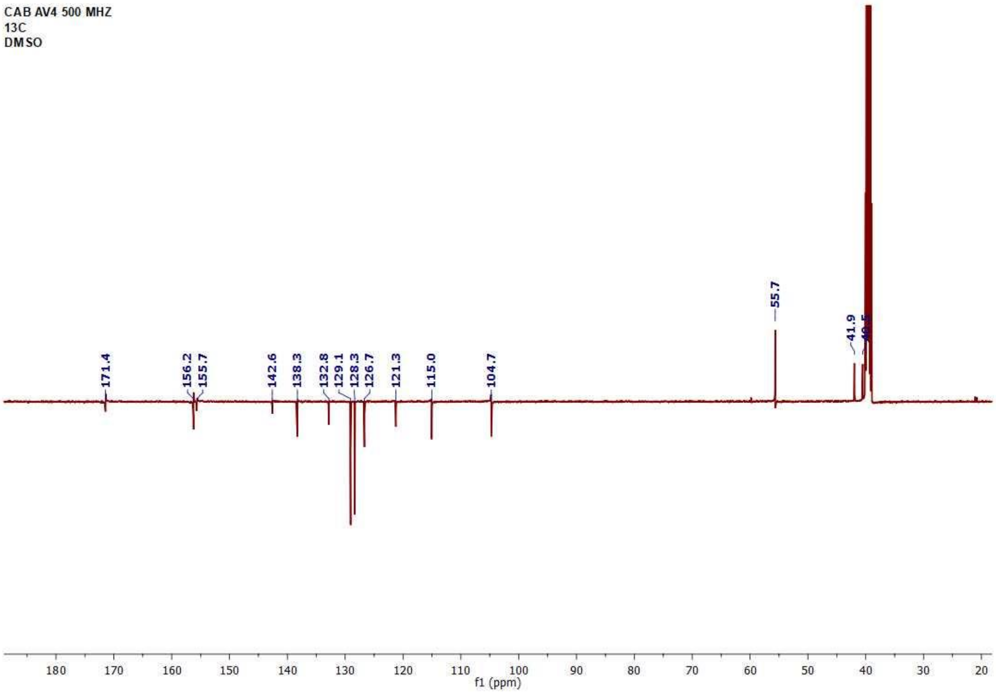

CAB AV4 500 MHZ
1H
DMSO

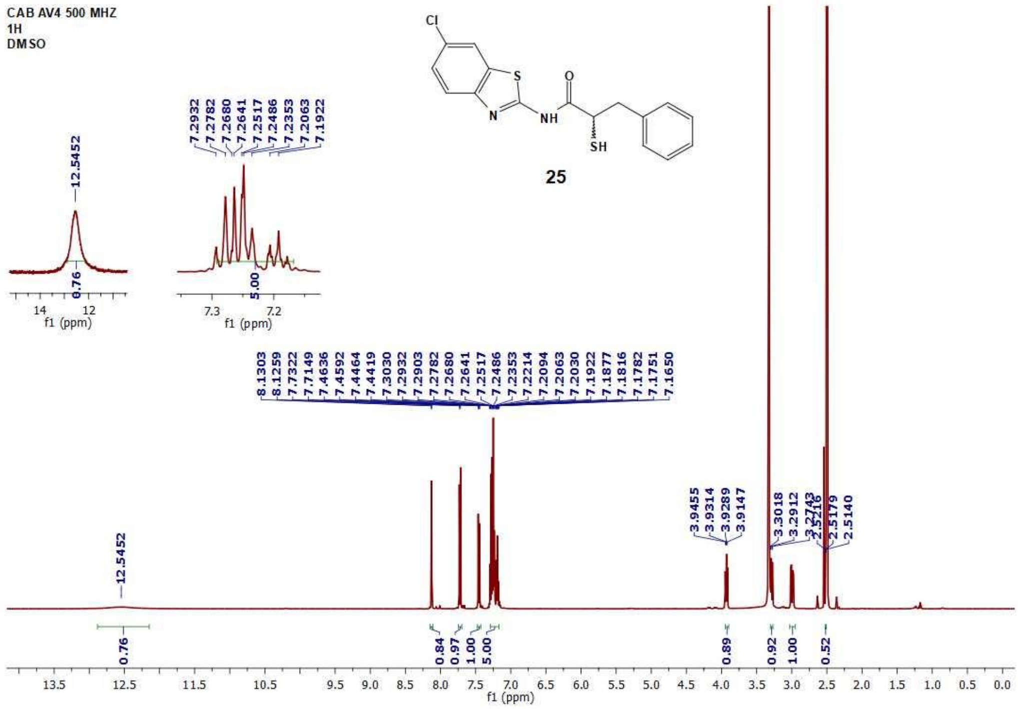

CAB AV4 500 MHZ
13C
DMSO

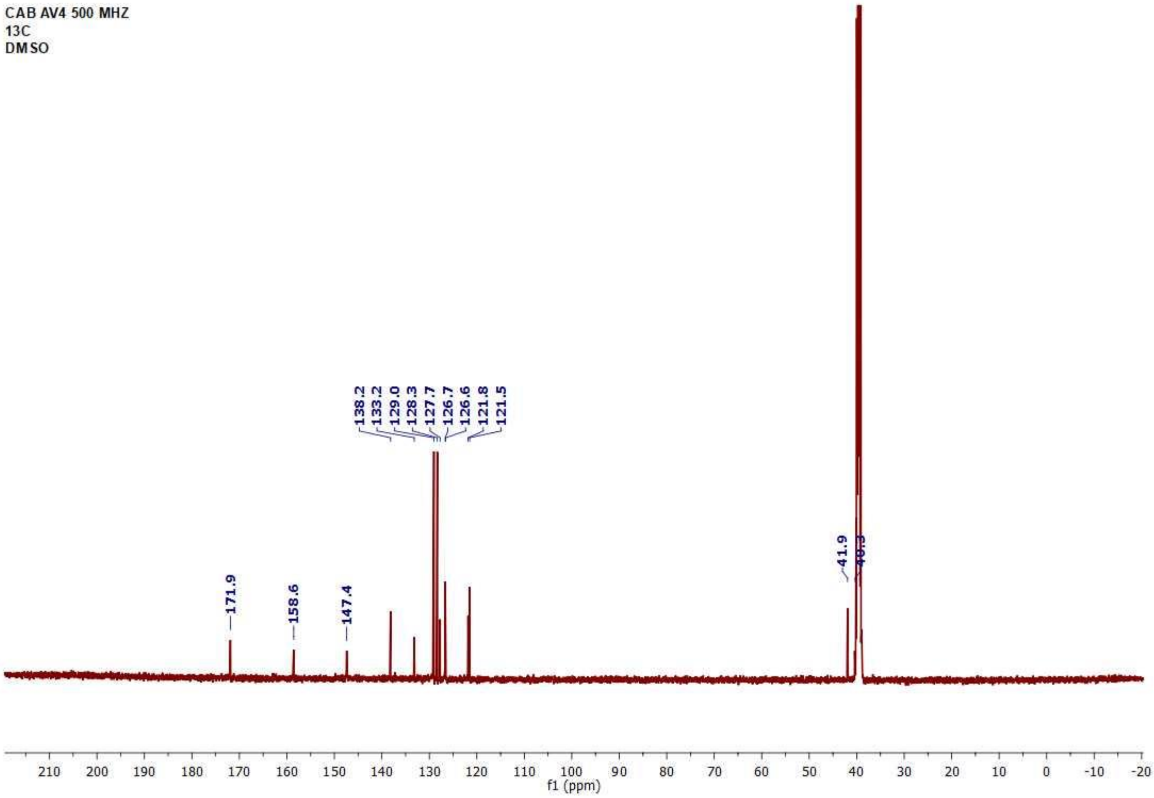

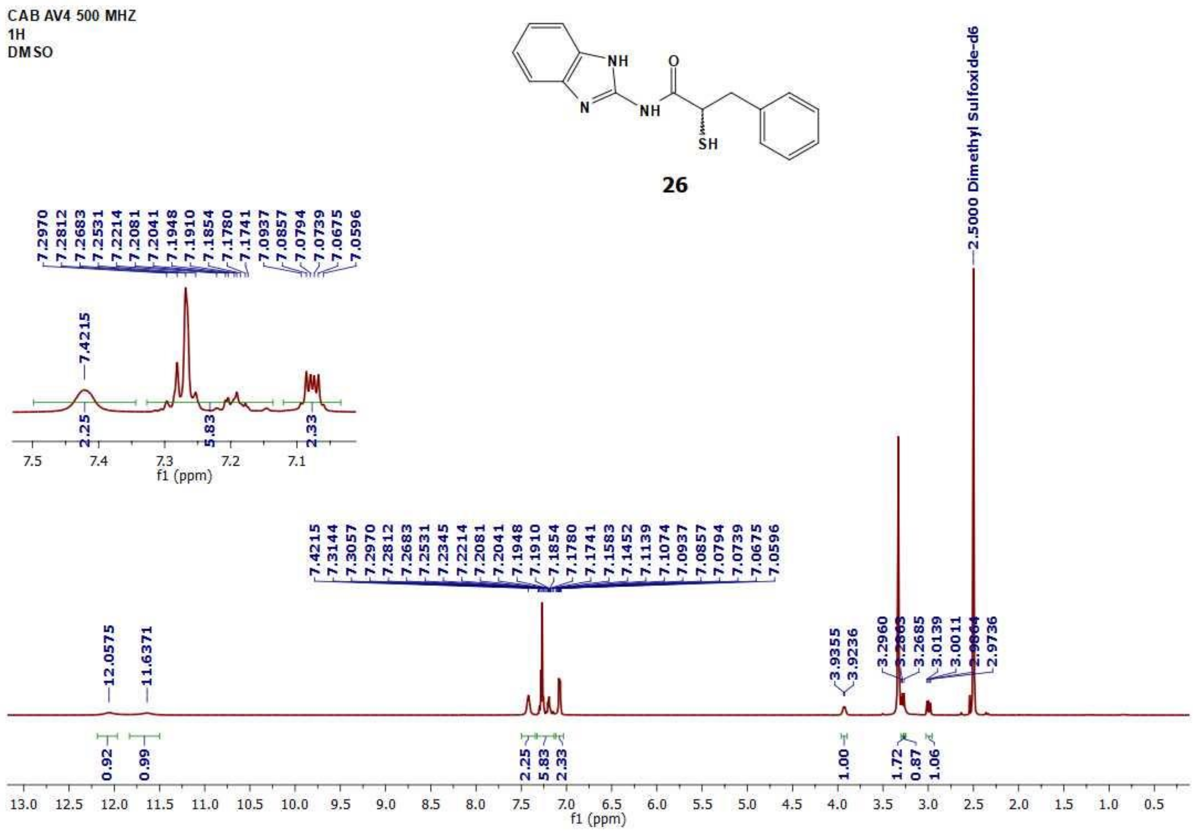

HRMS (ESI$^-$) m/z calcd. for Compound **26**, $C_{16}H_{16}N_3OS$ $[M–H]^-$ 298.10085, found 298.10069.

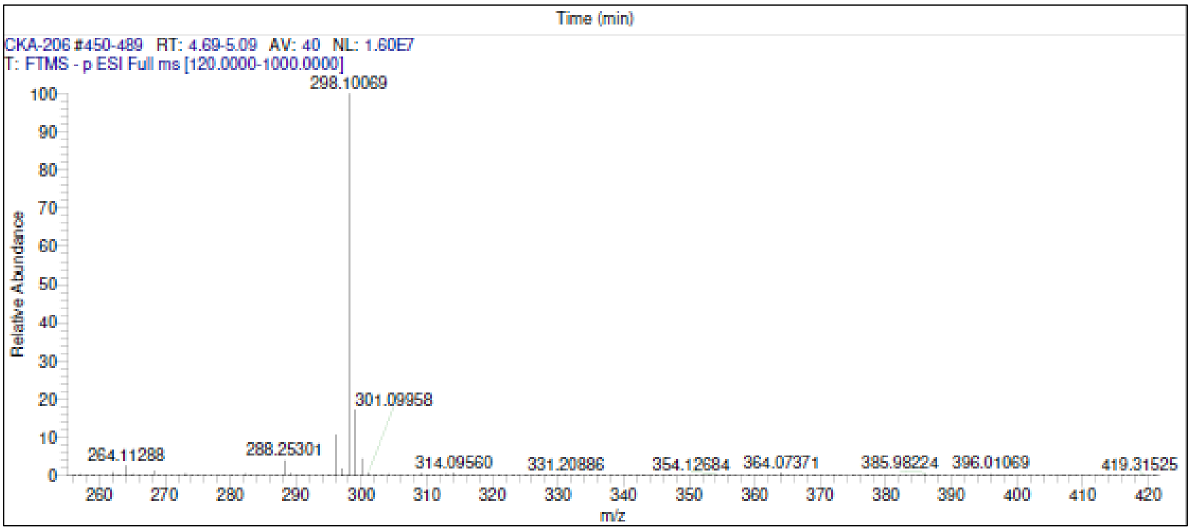

LC-MS Spectra of Final Compounds

Compound 11

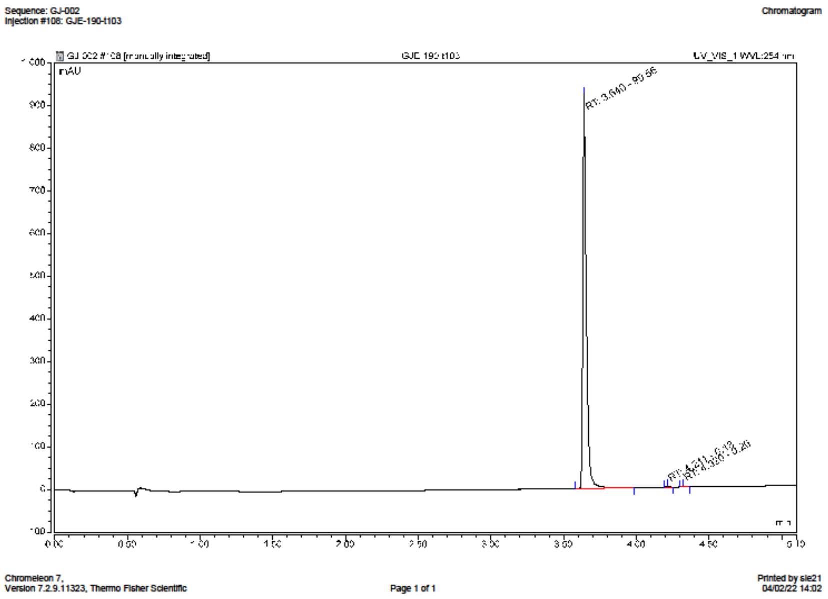

No.	Ret.Time min	Height mAU	Rel.Area %
1	3,64	926,387	99,56
2	4,211	1,932	0,18
3	4,32	2,64	0,26

Compound 12

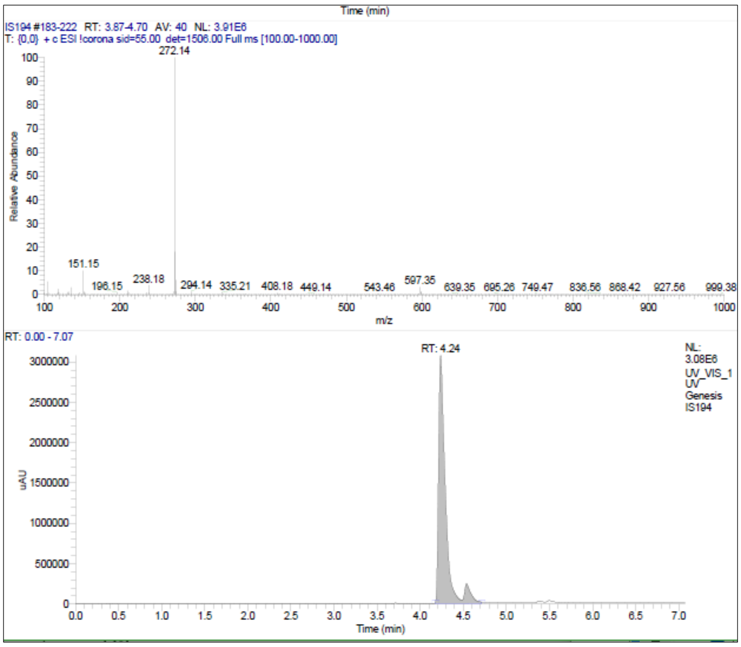

Apex RT	Start RT	End RT	Area	%Area	Height	%Height
4.24	4.19	4.46	15423541.207	91.50	3016184.824	93.52
4.54	4.48	4.70	1431948.365	8.50	250912.630	6.48

Compound 13

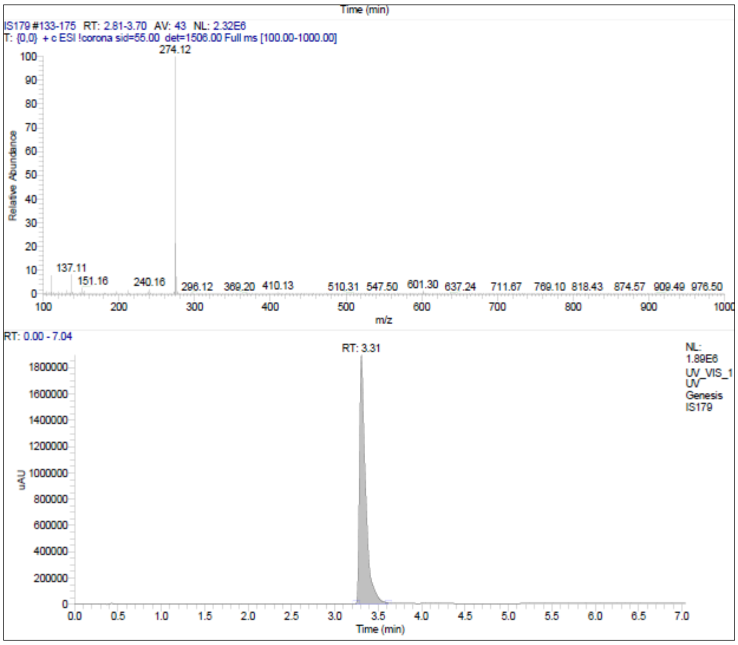

Apex RT	Start RT	End RT	Area	%Area	Height	%Height
3.31	3.25	3.62	9501387.828	100.00	1892528.208	100.00

Compound 14

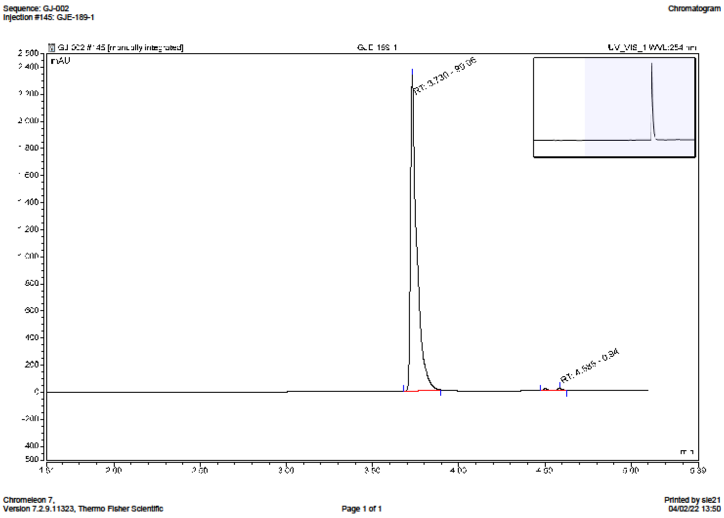

No.	Ret.Time min	Height mAU	Rel.Area %
1	3,73	2332,193	99,06
2	4,501	17,319	0,46
3	4,585	19,042	0,48

Compound 15

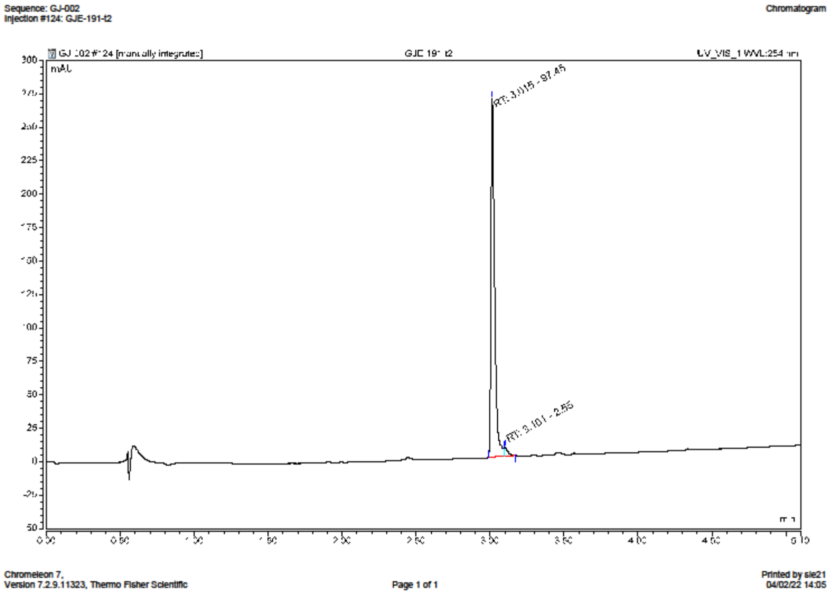

No.	Ret.Time<br>min	Height<br>mAU	Rel.Area<br>%
1	3,015	268,295	97,45
2	3,101	6,753	2,55

Compound 16

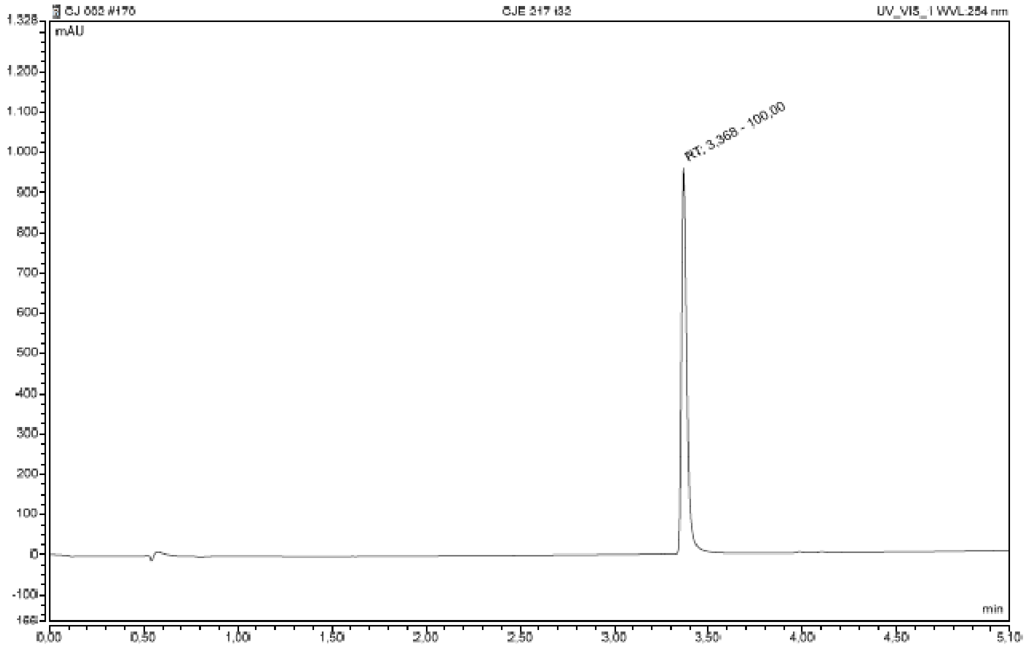

No.	Ret.Time<br>min	Height<br>mAU	Rel.Area<br>%
1	3,368	959,613	100

Compound 17

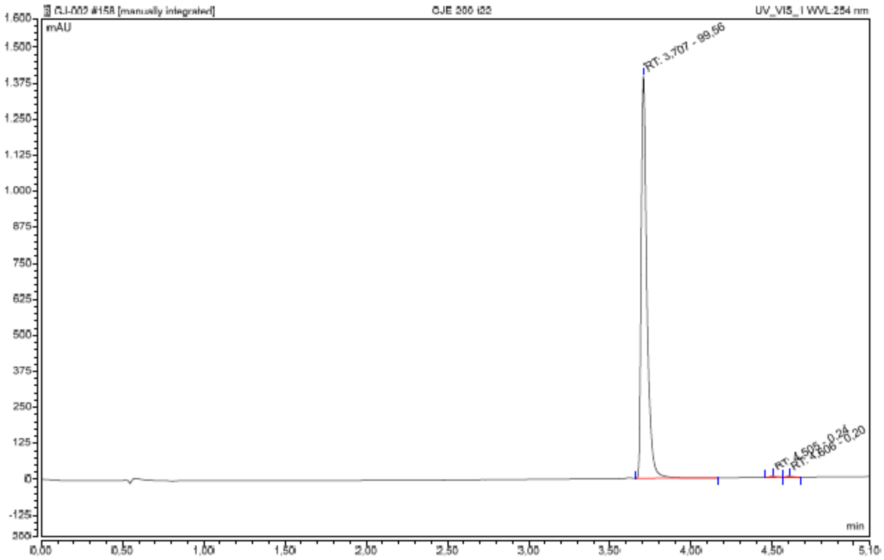

No.	Ret.Time min	Height mAU	Rel.Area %
1	3,707	1395,456	99,56
2	4,505	3,459	0,24
3	4,606	3,373	0,2

Compound 18

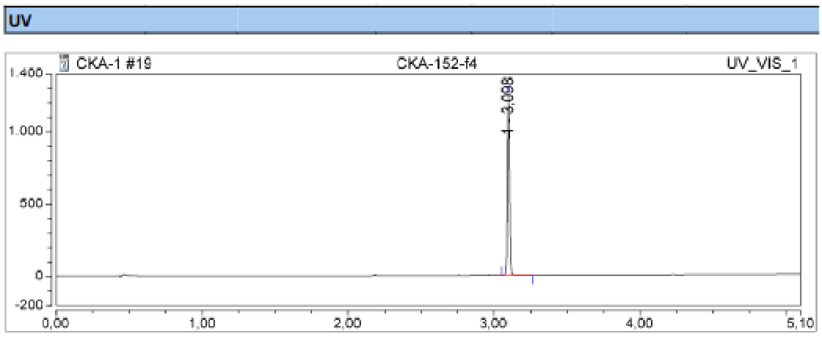

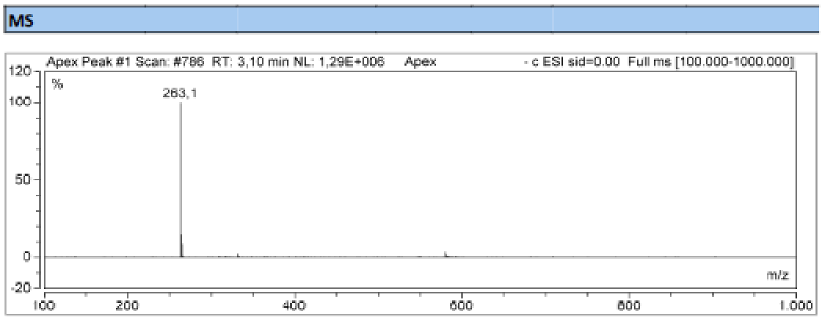

No.	Peak Name	Retention Time min	Area mAU*min	Height mAU	Relative Area %	Relative Height %
1		3,098	18,899	1244,923	100,00	100,00

Compound 19

UV

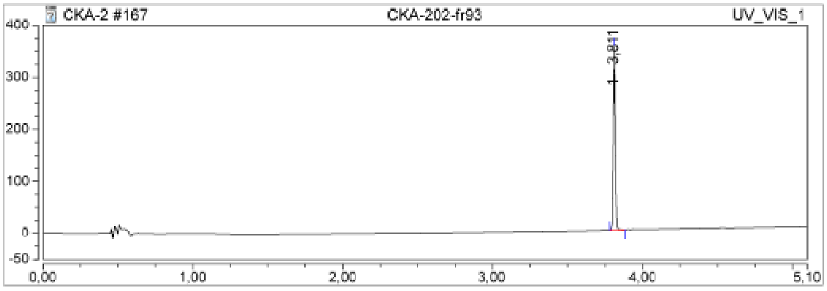

MS

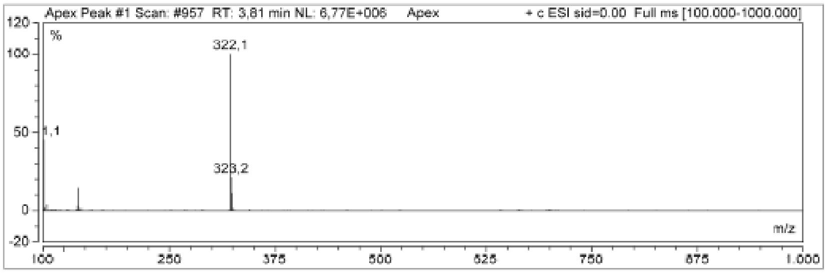

No.	Peak Name	Retention Time min	Area mAU*min	Height mAU	Relative Area %	Relative Height %
1		3,811	5,319	352,300	100,00	100,00

Compound 20

UV

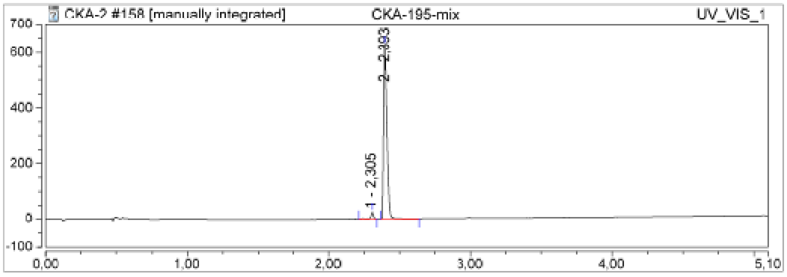

MS

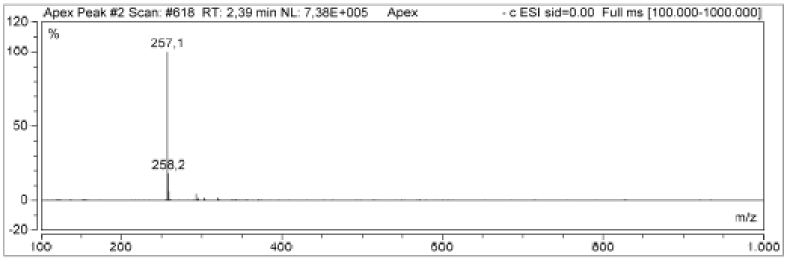

No.	Peak Name	Retention Time min	Area mAU*min	Height mAU	Relative Area %	Relative Height %
1		2,305	0,462	26,233	2,69	4,03
2		2,393	16,697	624,713	97,31	95,97

Compound 21

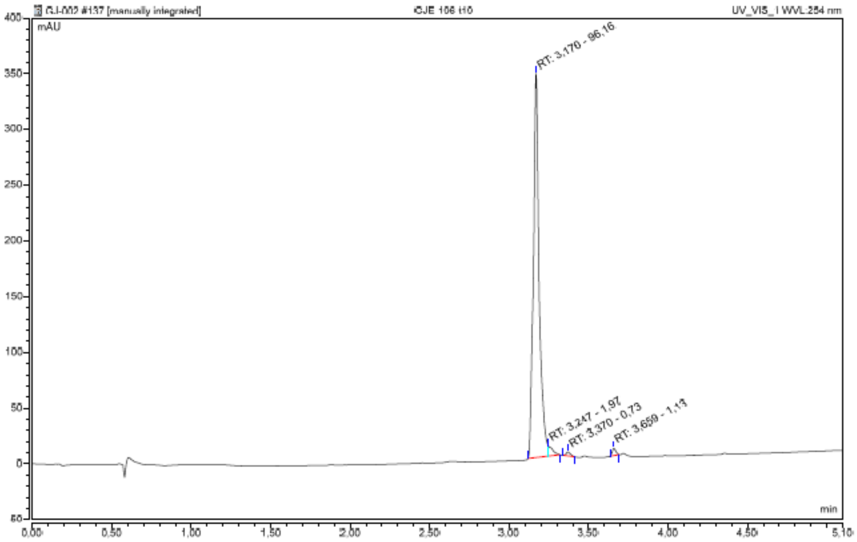

No.	Ret.Time<br>min	Height<br>mAU	Rel.Area<br>%
1	3,17	344,733	96,16
2	3,247	9,16	1,97
3	3,37	3,565	0,73
4	3,659	6,607	1,13

Compound 22

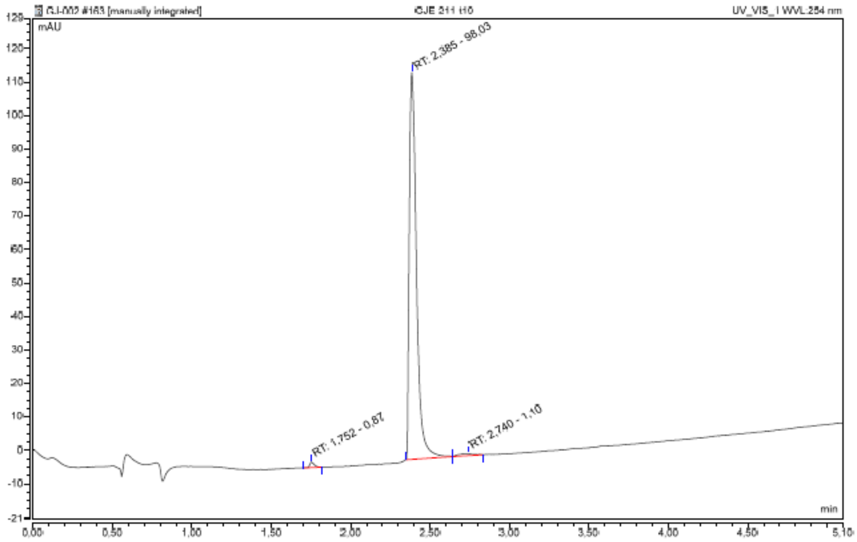

No.	Ret.Time<br>min	Height<br>mAU	Rel.Area<br>%
1	1,752	1,659	0,87
2	2,385	115,562	98,03
3	2,74	0,618	1,1

Compound 23

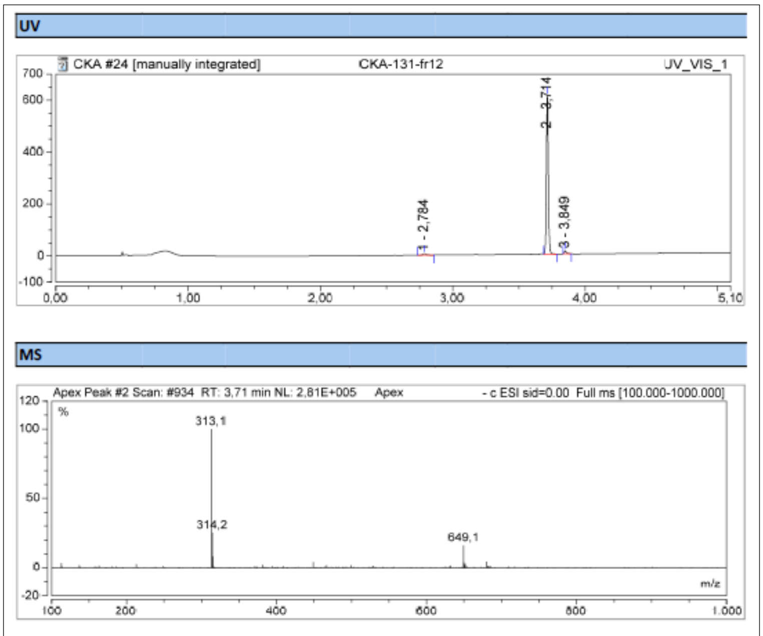

No.	Peak Name	Retention Time min	Area mAU*min	Height mAU	Relative Area %	Relative Height %
1		2,784	0,169	6,083	1,61	0,97
2		3,714	10,101	610,228	96,09	97,15
3		3,849	0,242	11,845	2,31	1,89

Compound 24

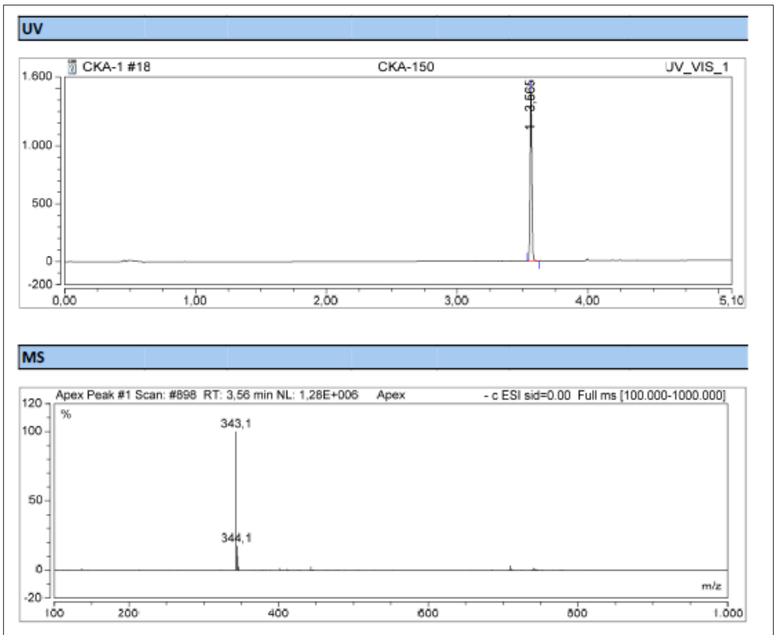

No.	Peak Name	Retention Time min	Area mAU*min	Height mAU	Relative Area %	Relative Height %
1		3,565	21,492	1479,383	100,00	100,00

Compound 25

UV

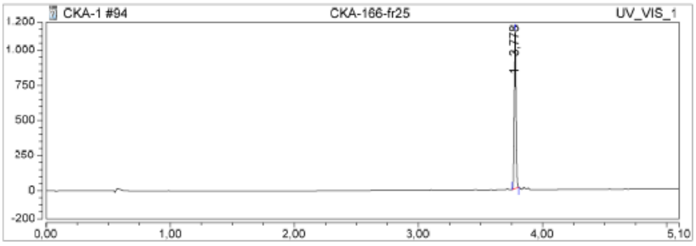

MS

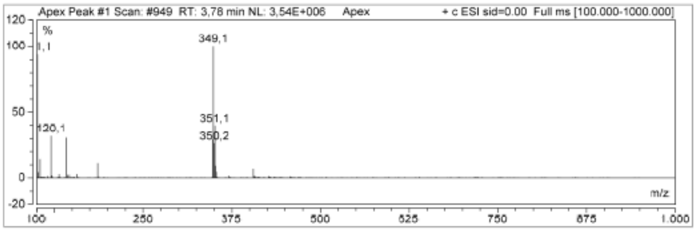

No.	Peak Name	Retention Time min	Area mAU*min	Height mAU	Relative Area %	Relative Height %
1		3,778	17,296	1116,019	100,00	100,00

Compound 26

UV

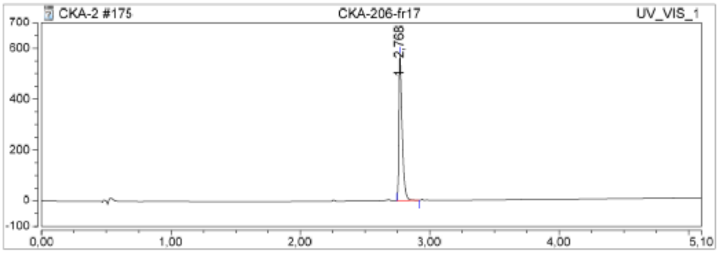

MS

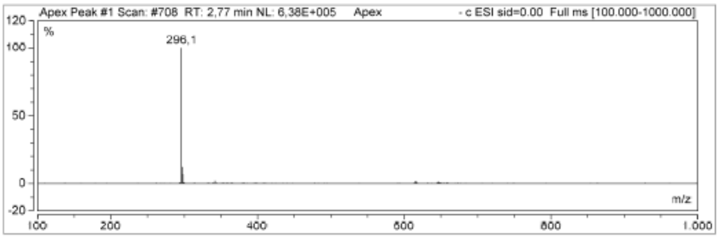

No.	Peak Name	Retention Time min	Area mAU*min	Height mAU	Relative Area %	Relative Height %
1		2,768	16,322	573,119	100,00	100,00

References

(1) Kany, A. M.; Sikandar, A.; Haupenthal, J.; Yahiaoui, S.; Maurer, C. K.; Proschak, E.; Köhnke, J.; Hartmann, R. W. Binding Mode Characterization and Early in Vivo Evaluation of Fragment-Like Thiols as Inhibitors of the Virulence Factor LasB from Pseudomonas Aeruginosa. *ACS Infect. Dis.* **2018**, *4* (6), 988–997. https://doi.org/10.1021/acsinfecdis.8b00010.

(2) Eckhard, U.; Schönauer, E.; Brandstetter, H. Structural Basis for Activity Regulation and Substrate Preference of Clostridial Collagenases G, H, and T. *J. Biol. Chem.* **2013**, *288* (28), 20184–20194. https://doi.org/10.1074/jbc.M112.448548.

(3) Schönauer, E.; Kany, A. M.; Haupenthal, J.; Hüsecken, K.; Hoppe, I. J.; Voos, K.; Yahiaoui, S.; Elsässer, B.; Ducho, C.; Brandstetter, H.; Hartmann, R. W. Discovery of a Potent Inhibitor Class with High Selectivity toward Clostridial Collagenases. *J. Am. Chem. Soc.* **2017**, *139* (36), 12696–12703. https://doi.org/10.1021/jacs.7b06935.

(4) Murphy, D. J. Determination of Accurate KI Values for Tight-Binding Enzyme Inhibitors: An in Silico Study of Experimental Error and Assay Design. *Anal. Biochem.* **2004**, *327* (1), 61–67. https://doi.org/10.1016/j.ab.2003.12.018.

(5) Morrison, J. F. Kinetics of the Reversible Inhibition of Enzyme-Catalysed Reactions by Tight-Binding Inhibitors. *Biochim. Biophys. Acta - Enzymol.* **1969**, *185* (2), 269–286. https://doi.org/10.1016/0005-2744(69)90420-3.

(6) Kuzmič, P.; Elrod, K. C.; Cregar, L. M.; Sideris, S.; Rai, R.; Janc, J. W. High-Throughput Screening of Enzyme Inhibitors: Simultaneous Determination of Tight-Binding Inhibition Constants and Enzyme Concentration. *Anal. Biochem.* **2000**, *286* (1), 45–50. https://doi.org/10.1006/abio.2000.4685.

(7) Kany, A. M.; Sikandar, A.; Yahiaoui, S.; Haupenthal, J.; Walter, I.; Empting, M.; Köhnke, J.; Hartmann, R. W. Tackling Pseudomonas Aeruginosa Virulence by a Hydroxamic Acid-Based LasB Inhibitor. *ACS Chem. Biol.* **2018**, *13* (9), 2449–2455. https://doi.org/10.1021/acschembio.8b00257.

(8) Haupenthal, J.; Baehr, C.; Zeuzem, S.; Piiper, A. RNAse A-like Enzymes in Serum Inhibit the Anti-Neoplastic Activity of SiRNA Targeting Polo-like Kinase 1. *Int. J. Cancer* **2007**, *121* (1), 206–210. https://doi.org/10.1002/ijc.22665.

(9) SeeSAR v.11.1, BioSolveIT GmbH, Sankt Augustin, G. 2021. Available from: Http://Www.Biosolveit.de/SeeSAR.

(10) Schrödinger, L., & DeLano, W. (2020). P. R. from http://www. pymol. org/pymo. The PyMOL Molecular Graphics System, Version 2.0 Schrödinger, LLC. 2021.

(11) Chakraborty, C.; Sharma, A. R.; Sharma, G.; Lee, S. S. Zebrafish: A Complete Animal Model to Enumerate the Nanoparticle Toxicity. *J. Nanobiotechnology* **2016**, *14* (1), 1–13. https://doi.org/10.1186/s12951-016-0217-6.

(12) Casilag, F.; Lorenz, A.; Krueger, J.; Klawonn, F.; Weiss, S.; Häussler, S. The LasB Elastase of Pseudomonas Aeruginosa Acts in Concert with Alkaline Protease AprA To Prevent Flagellin-Mediated Immune Recognition. *Infect. Immun.* **2016**, *84* (1), 162–171. https://doi.org/10.1128/IAI.00939-15.

(13) Tanasova, M.; Yang, Q.; Olmsted, C. C.; Vasileiou, C.; Li, X.; Anyika, M.; Borhan, B. An Unusual Conformation of α-Haloamides Due to Cooperative Binding with Zincated Porphyrins. *European J. Org. Chem.* **2009**, No. 25, 4242–4253. https://doi.org/10.1002/ejoc.200900089.

(14) Vijayakumar, V. N.; Jayaprakasam, R. Nanoliquid Crystals: A Study of Permeability and Optical Measurements in Nematic Phase. *Macromol. Symp.* **2016**, *361* (1), 112–116. https://doi.org/10.1002/masy.201400260.

(15) Zhuze, A. L.; Jošt, K.; Kasafírek, E.; Rudinger, J. Amino Acids and Peptides. XLV. Analogues of Oxytocin with O-Ethyltyrosine, p-Methylphenylalanine, and p-Ethylphenylalanine Replacing Tyrosine. *Collect. Czechoslov. Chem. Commun.* **1964**, *29* (11), 2648–2662. https://doi.org/10.1135/cccc19642648.

(16) Schmidt, R. R. Synthesis and Herbicidal Activity o f d (+) and 1(_) Methyl-2-Chloro-3-(4-Chlorophenyl)-Propionate. **1976**, *1*, 7–9.

(17) Hydrochloride, N.; Lee, W. W.; Tong, G. L.; Martinez, A. P.; Weinstein, B.; Schelstraete, M. G. M.; Baker, B. R.; Goodman, L.; Baker, S. Synthesis of N , N-Bis (2-Chloroethyl) -DL-Phenylalanine Hydrochloride1. **1963**, *275* (1953), 1954–1957.

(18) Vora, H. U.; Rovis, T. Nucleophilic Carbene and HOAt Relay Catalysis in an Amide Bond Coupling: An Orthogonal Peptide Bond Forming Reaction. *J. Am. Chem. Soc.* **2007**, *129* (45), 13796–13797. https://doi.org/10.1021/ja0764052.

(19) Liu, P. V. Extracellular Toxins of Pseudomonas Aeruginosa. *J. Infect. Dis.* **1974**, *130* (November), S94–S99. https://doi.org/10.1093/infdis/130.Supplement.S94.
